# First Demonstration of *In Vivo* PDE11A4 Target Engagement for Potential Treatment of Age-Related Memory Disorders

**DOI:** 10.1021/acs.jmedchem.4c01794

**Published:** 2024-09-25

**Authors:** Shams ul Mahmood, Jeremy Eberhard, Charles S. Hoffman, Dennis Colussi, John Gordon, Wayne Childers, Elvis Amurrio, Janvi Patel, Michy P. Kelly, David P. Rotella

**Affiliations:** † Department of Chemistry and Biochemistry, Montclair State University, 1 Normal Avenue, Montclair, New Jersey 07043, United States; ‡ Biology Department, Boston College, Chestnut Hill, Massachusetts 02467, United States; § Moulder Center for Drug Discovery Research, Temple University, Philadelphia, Pennsylvania 19140, United States; ∥ Department of Neurobiology, University of Maryland School of Medicine, Baltimore, Maryland 21201, United States; ⊥ Center for Research on Aging, University of Maryland, Baltimore School of Medicine, Baltimore, Maryland 21201, United States; # Sokol Institute for Pharmaceutical Life Sciences, Montclair State University, Montclair, New Jersey 07043, United States

## Abstract

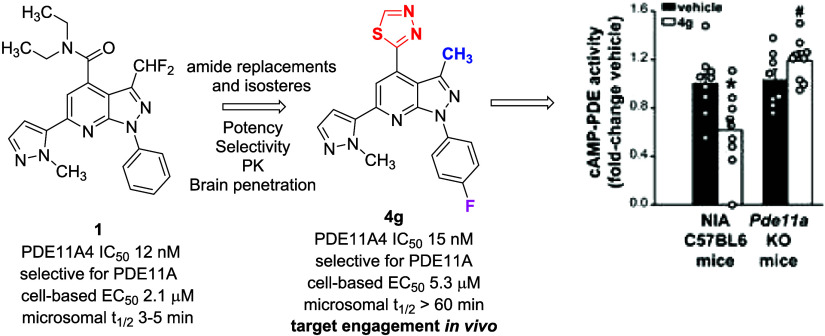

PDE11A4 is a target of interest for the treatment of age-related memory disorders. A previous report from our laboratories described an amide series of potent, selective PDE11A4 inhibitors that was metabolically unstable. Investigation of heterocyclic amide isosteres for the labile amide moiety revealed distinct structure–activity relationships and identified several compounds with potency comparable to the amide series. This manuscript describes the characterization of structure–activity and structure–property relationships in this set, leading to the identification of an orally bioavailable, brain-penetrant, selective and potent PDE11A4 inhibitor. Target engagement experiments demonstrated PDE11A4 inhibition in the hypothalamus of mice that was absent in PDE11A4 knock out animals.

## Introduction

PDE11A4 is a member of the family of mammalian cyclic nucleotide phosphodiesterases that regulates levels of cyclic AMP (cAMP) and cyclic GMP (cGMP), important intracellular second messengers essential for a wide variety of functions.^1−3^ This dual specificity phosphodiesterase is highly expressed in the hypothalamus, extended hippocampal formation, and to a lesser extent in the retina, spinal cord, and dorsal root ganglia.^1,4−7^ Pilarzyk and co-workers showed that hippocampal expression of PDE11A4 increased with age in rodents and humans,^6^ and that preventing or reversing this age-related increase in PDE11A4 expression reduced age-related deficits in associative long-term social memory.^6,8^

**Figure 1:**
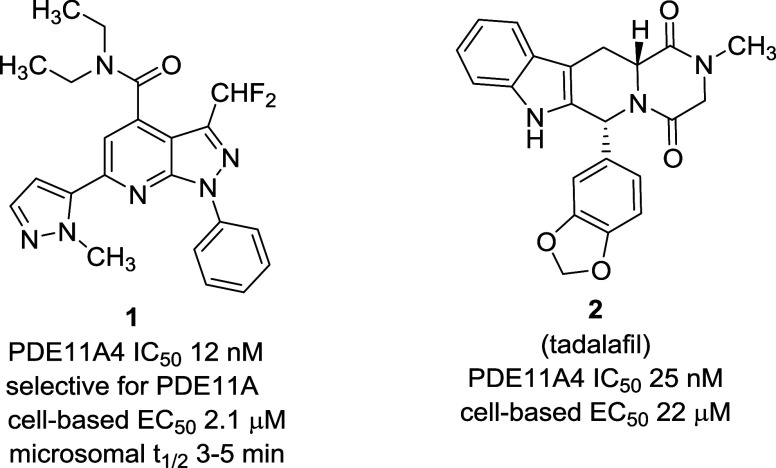
Known PDE11A4 inhibitors.

We are investigating PDE11A4 small molecule inhibitors for the potential treatment of age-related cognitive decline and recently reported the discovery and characterization of a novel series of pyrazolopyridine amides (*e.g*., **1**, Figure 1) that exhibited potent, selective PDE11A4 inhibition, with cellular activity superior to the known PDE11A inhibitor tadalafil (**2**).^9^ The most potent and selective example in this series, **1**, was rapidly metabolized at the diethyl amide in mouse and human liver microsomal preparations, limiting use in preclinical models of age-related memory decline. This stimulated the exploration of amide isosteres and replacements to solve this problem.^10^

We now report the identification of a metabolically stable, potent and selective PDE11A4 inhibitor that is brain penetrant, orally bioavailable and demonstrates target engagement in the hypothalamus as a prelude to investigation in animal models of age-related cognitive decline. The discovery of this candidate revealed distinct PDE11A4 structure–activity, potency and selectivity determinants in these new compounds compared to the amide series exemplified by **1**.

## Results

**Scheme 1:**
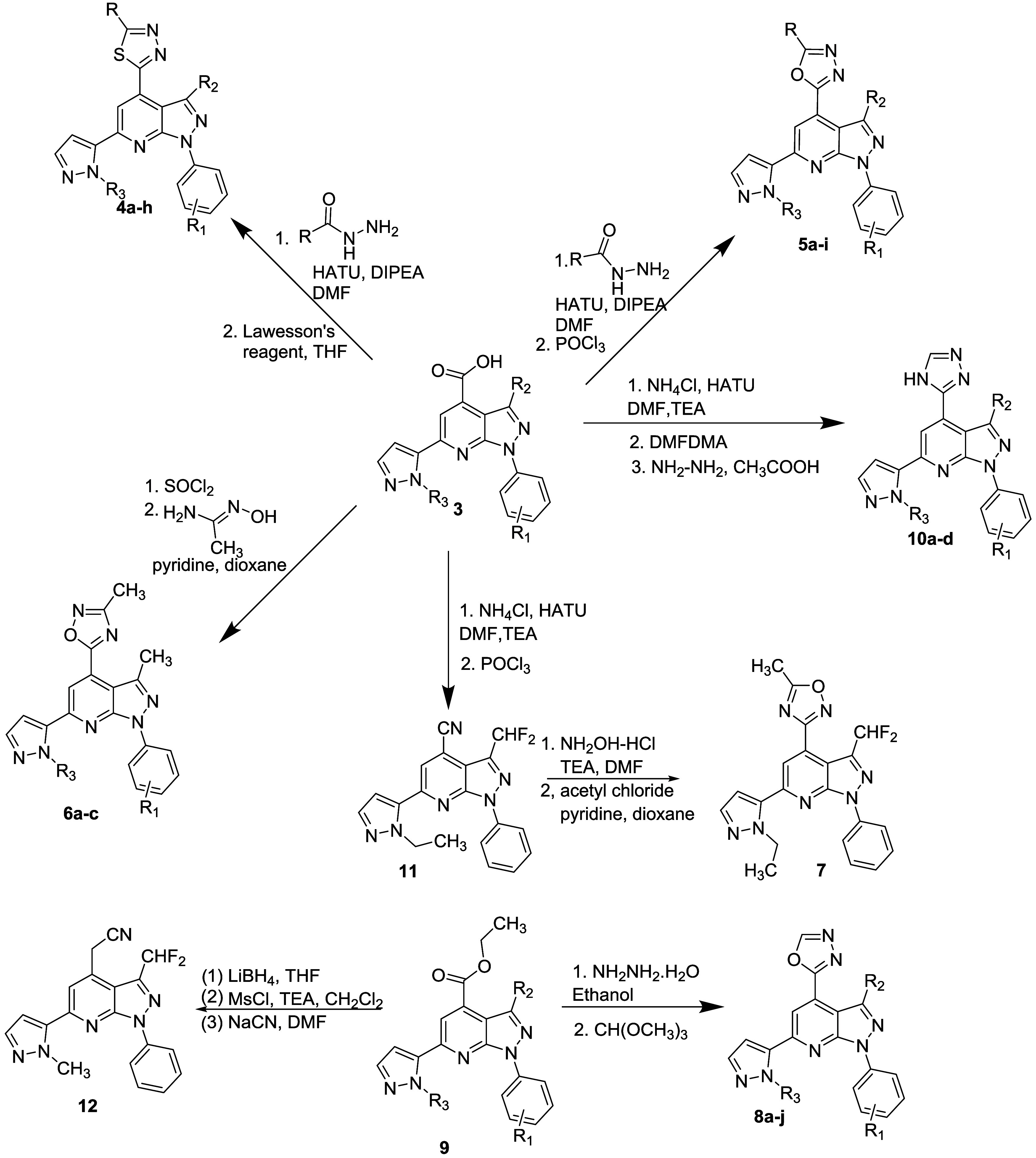
Synthesis of PDE11A4 Inhibitors

As noted previously,^9^ amide structure–activity information suggested the binding site for this moiety in PDE11A4 was sterically limited and nonpolar. *In vitro* experiments using human and mouse microsomes highlighted the amide alkyl groups as the predominant site for metabolism and for this reason we focused attention on replacements such as heterocyclic isosteres.^10^ The synthesis of 1,3,4-thiadiazole analogs **4a**–**h** proceeded in straightforward manner from carboxylic acids **3** by acyl hydrazide coupling followed by treatment with Lawesson’s reagent (Scheme 1).^11^ Similarly, 2-alkyl–1,3,4-oxadiazoles **5a**–**i** were prepared from the appropriate acyl hydrazides and phosphorus oxychloride.^12^ Regioisomeric 3-methyl–1,2,4-oxadiazoles **6a**–**c** were prepared in two steps from acids **3** by acid chloride formation followed by reaction with *N*-hydroxyacetamidine in pyridine/dioxane.^13^ 5-Methyl–1,2,4-oxadiazole isomer **7** was prepared from nitrile **11** that was obtained by POCl_3_-mediated dehydration of the primary amide obtained from **3**.^14^ Unsubstituted 1,3,4-oxadiazole analogs **8a**–**j** were prepared from esters **9** by treatment with hydrazine, followed by cyclization using trimethyl orthoformate.^15^ 1,3,4-Triazoles **10a**–**d** were prepared from acids **3** in three steps as shown in Scheme 1.^16^ Nitrile **12** was prepared in a straightforward manner from ester **9** by lithium borohydride reduction, mesylation of the intermediate primary alcohol, and cyanide displacement.

These inhibitors were evaluated for activity against human PDE11A4 using cAMP as substrate with **1** as a positive control in a screening mode at 50 and 500 nM. In general, analogs that displayed approximately 70% inhibition at 50 nM progressed for IC_50_ determination (Table 1). Analogs with promising PDE11A4 potency (IC_50_ < 50 nM) were evaluated for PDE selectivity versus PDEs 3, 4, 5, 6, and 10. These PDEs were selected based on structural similarity (PDEs 5, 6,10) to PDE11A4, as well as for adverse event risks (PDEs 3, 4). This was followed by *in vitro* ADME evaluation for liver microsome stability and aqueous solubility, cellular efficacy and pharmacokinetic exploration, brain penetration and brain tissue binding.

**Table 1:**
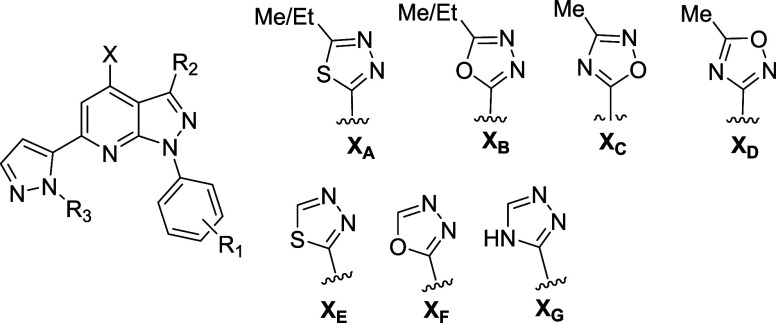
PDE11A4 Inhibition (IC_50_ Average of **3** Independent Determinations)

Cpd	X	R_1_	R_2_	R_3_	IC_50_ (nM)/% inhib@500 nM
**4a**	X_A_	H	CH_3_	Et	175
**4b**	X_A_ (Et)	H	CH_3_	Et	53%
**4c**	X_A_	4-F	H	Et	121
**4d**	X_A_ (Et)	4-F	H	Et	310
**4e**	X_A_	4-F	CH_3_	Et	117
**4f**	X_A_	H	CHF_2_	Me	107
**4g**	X_E_	4-F	CH_3_	Me	15
**4h**	X_E_	4-F	CH_3_	Et	34
**5a**	X_B_	H	CHF_2_	Et	93
**5b**	X_B_	H	CH_3_	Et	69
**5c**	X_B_ (Et)	H	CH_3_	Et	62%
**5d**	X_B_	4-F	H	Et	500
**5e**	X_B_	2-F	H	Et	55%
**5f**	X_B_	4-F	CH_3_	Et	75
**5g**	X_B_	2-Cl	CH_3_	Me	64%
**5h**	X_B_	H	CH_3_	Me	158
**5i**	X_B_	H	CHF_2_	Me	93
**6a**	X_C_	2-F	CH_3_	Et	69%
**6b**	X_C_	H	CH_3_	Me	514
**6c**	X_C_	H	CHF_2_	Me	69%
**7**	X_D_	H	CHF_2_	Me	50%
**8a**	X_F_	H	CHF_2_	Me	27
**8b**	X_F_	2-F	CH_3_	Et	30
**8c**	X_F_	4-F	CHF_2_	Et	41
**8d**	X_F_	2-F	H	Et	84
**8e**	X_F_	4-F	H	Et	59
**8f**	X_F_	4-F	CH_3_	Et	15
**8g**	X_F_	2,4-diF	CH_3_	Et	15
**8h**	X_F_	H	CH_3_	Et	51
**8i**	X_F_	4-F	CH_3_	Me	42
**8j**	X_F_	2,4-diF	CH_3_	Me	39
**10a**	X_G_	2-F	CH_3_	Et	47
**10b**	X_G_	H	CH_3_	Me	36
**10c**	X_G_	4-F	CH_3_	Me	44
**10d**	X_G_	H	CHF_2_	Me	55
**11**	CN	H	CHF_2_	Et	45
**12**	CH_2_CN	H	CHF_2_	Me	42
**1**					12

This survey began by evaluating a series of 2-alkyl–1,3,4-thiadiazoles **4a**–**f** (X_A_) (Table 1). Direct comparison to amide **1** with **4f** revealed nearly a 10-fold drop in potency. Fluorination at the 4-position of the phenyl ring (**4c**), like the amide series, had little or no measurable effect on activity, and interestingly, unlike previous observations, removal of the alkyl group from the pyrazolopyridine scaffold also had no effect (**4e***vs***4c**). Ethyl substitution on the thiadiazole isostere (**4b** and **4d**) resulted in decreased activity compared to methyl substitution (**4a** and **4c**), supporting the steric limitations observed previously. Difluoromethyl substitution on the scaffold (**4f***vs***4a**) provided a small improvement in potency.

Comparison of these sulfur-based isosteres with the corresponding and less lipophilic oxygen analogs (**5a-I**, X_B_) showed that the oxa–3,4-diazoles could be (**5b***vs***4a**), but were not always slightly more potent (**5i***vs***4f**). Unlike the thiadiazoles, removal of the pyrazolopyridine alkyl substituent (R_2_ = H) reduced potency (**5d**/**5e**). 4-Fluorination (**5f**) did not improve potency, compared to the unsubstituted derivative (**5b**). Interestingly, and unlike the amide series, both the 2-fluoro (**5e**) and 2-chloro (**5g**) derivatives were measurably less potent compared to either the unsubstituted or 4-fluorophenyl analogs. Steric effects once again were evident by the substantially decreased activity of ethyl-substituted analog **5c**. Both regioisomeric 1,2,4-oxadiazoles (**6a**–**c** and **7**) were substantially less potent. Results with **7**, compared to **5a**, are noteworthy because they suggest selective interactions with the isostere in the binding site. The dihedral angle between the pyrazolopyridine and oxadiazole is similar in both molecules, at 35 (**5a**) and 39° (**7**). Other torsional angles in these two molecules are essentially identical.

Removal of the methyl group in the 1,3,4-oxadiazole isostere (**8a**–**j**, X_F_) resulted in analogs with a small increase in potency in some instances relative to their 2-methyl-substituted analogs. For example, **8a** is approximately 3-fold more potent compared to **5a**, **8d** and **8e** are more potent than **5e** and **5d**. However, **8h** and **5b** are comparable, as are **8i** and **5f**. Unlike the amide series, there is little or no effect on potency associated with the pyrazolopyridine substituent (cf. **8c** (CHF_2_), **8e** (H), **8i** (CH_3_)). There is a small positive effect resulting from 4-fluorination (**8f***vs***8h**), and unlike the observation in the 2-methyl–1,3,4-oxadiazoles, 4-fluorination is comparable to the 2-postion (**8f***vs***8b**). Two analogs in this group, **8f** and **8g** were equipotent with diethylamide **1**. In the analogous small set of 1,3,4-thiadiazoles (**4g**/**h**, X_E_), removal of the methyl group results in a more substantial improvement in potency, and one analog, **4g**, is equipotent with its oxa analog **8f**.

The final isostere option investigated was a set of four 1,3,4-triazoles (X_G_, **10a**–**d**) where analogs were selected in part on the SAR established above. As the data in Table 1 show, all four compounds in this group show good PDE11A4 potency, and substituents on the pendant phenyl ring, including 2-fluoro substitution (**10a**) or scaffold have little or no effect on potency in any of these analogs. We also evaluated two nitrile intermediates, **11** and **12**, and both compounds provide analogs with IC_50_ values less than 50 nM, comparable to the unsubstituted oxa- and thia–1,3,4-diazoles.

Viewed collectively, these results illustrate that the most active diazole isosteres (X_A_, X_B_, X_E-G_) display distinct PDE11A4 potency structure–activity profiles in which modifications are not generally applicable, illustrated by the limited number of matched pairs prepared in these subsets. Each preferred isostere also has similarity and differences compared to the amide series. We explored the utility of modeling to help understand these observations. Two different and independently developed homology-based binding models were unable to provide an explanation to rationalize these results or to explain SAR within a series. One homology model was derived from a human PDE5A-based structure (PDB code 6IWI), and the other from human PDE10 (PDB code 5SIL).

In the set of 12 compounds with PDE11A4 activity less than 50 nM, notable differences were apparent when screened at 50 and 500 nM for selectivity against PDEs 3, 4, 5, 6, and 10 as shown in Table 2. This PDE subset was chosen based on a combination of adverse event risk and structural similarity to PDE11A.^9^ Nitriles **11** and **12** were potent PDE4D3 inhibitors at 500 nM (>95%), eliminating them from more detailed consideration. Unlike sulfur- or oxygen-based isosteres, triazoles **10a**–**d** display greater than 80% PDE6C inhibition appreciable inhibition (>50%) of PDE5A at 500 nM. In 1,3,4-oxadiazoles, inhibition of PDE4D3 depends on the position of the fluorine substituent, with less inhibition by a 4-fluoro analog (**8c**), compared to the unsubstituted (**8a**) 2-fluoro (**8b**) or the 2,4-difluoro compounds (**8h**). This set displays another aspect of selectivity that was not evident in the diethylamide series, a measurable difference in PDE4D3 selectivity associated with the pyrazolopyridine substituent where a difluoromethyl substituent (**8c**) confers improved selectivity compared to the methyl analog (**8g**), however both compounds retain more than 50% inhibition of PDE10. There is a notable difference between the oxadiazoles **8f** and **8g** and thiadiazoles **4g** and **4h** in PDE selectivity. Both **4g** and **4h** are weak inhibitors of PDEs 3, 4, and 5 with moderate PDE6 inhibition; **4h** is also a moderate PDE10 inhibitor. This data indicates that, with the possible exception of PDE6C, 1,3,4-thiadiazole **4g** has comparable selectivity to amide **1** and led to assessment against other PDEs to get a more complete view of activity against others in the superfamily. We included **4h** in this screen based on the similarity in the profile of these two PDE11A4 inhibitors and that data is shown in Table 3. Both thiadiazoles show good selectivity against this expanded panel. Both compounds show moderate inhibition of PDEs 2A and 8A compared to **1**; **4g** also has moderate inhibition of PDE9A. The PDE selectivity profile of **4g** remains suitable for use as a tool compound.

**Table 2:** Initial PDE Selectivity Screen (% Inhibition at 500 nM; Average of 2 Independent Determinations)

Cpd	PDE3A	PDE4D3	PDE5A	PDE6C	PDE10A
**1**(9)	2	8	20	7	8
**4g**	0	24	33	51	28
**4h**	0	25	34	47	51
**8a**	14	73	44	77	34
**8b**	15	68	45	85	50
**8c**	5	13	28	33	65
**8f**	11	61	38	63	31
**8g**	4	84	38	56	58
**10a**	20	17	65	80	35
**10b**	12	12	63	85	32
**10c**	10	10	55	83	41
**10d**	15	25	63	90	43
**11**	NT	97	NT	NT	NT
**12**	NT	95	NT	NT	NT

**Table 3:** Expanded PDE Selectivity (% Inhibition@1 μM; Average of 2 Independent Determinations)

Cpd	PDE1A	PDE2A	PDE7B	PDE8A	PDE9A
**1**(9)	3	12	2	1	1
**4g**	19	40	0	19	30
**4h**	20	40	0	19	0

This promising selectivity profile of **4g** and **4h** led to assessment of *in vitro* pharmaceutical properties as shown in Table 4. Both molecules had low aqueous solubility in pH 7.4 phosphate buffer (0.9 and 0.6 μM, respectively). Both demonstrated excellent stability in mouse liver microsomes with half-lives greater than 60 min, and good stability in human liver microsomes. Neither compound showed evidence of p–glycoprotein-mediated efflux in MDCK-MDR1 cell culture. Evaluation of the unbound fraction in a brain tissue binding assay shows **4g** is more than 99.9% bound and its plasma protein binding was not evaluated (Table 4).

**Table 4:** *In Vitro* Pharmaceutical Properties

Cpd	solubility (μM, pH 7.4 buffer)	half-life (min) mouse microsomes	half-life (min) human microsomes	rate B/A and A/B	efflux ratio (MDCK MDR1 cells)	brain tissue binding (fraction unbound)
**4g**	0.9	>60	57	6.13:7.65	0.8	<0.1
**4h**	0.6	>60	48	9.14:9.52	0.96	NT

**Figure 2:**
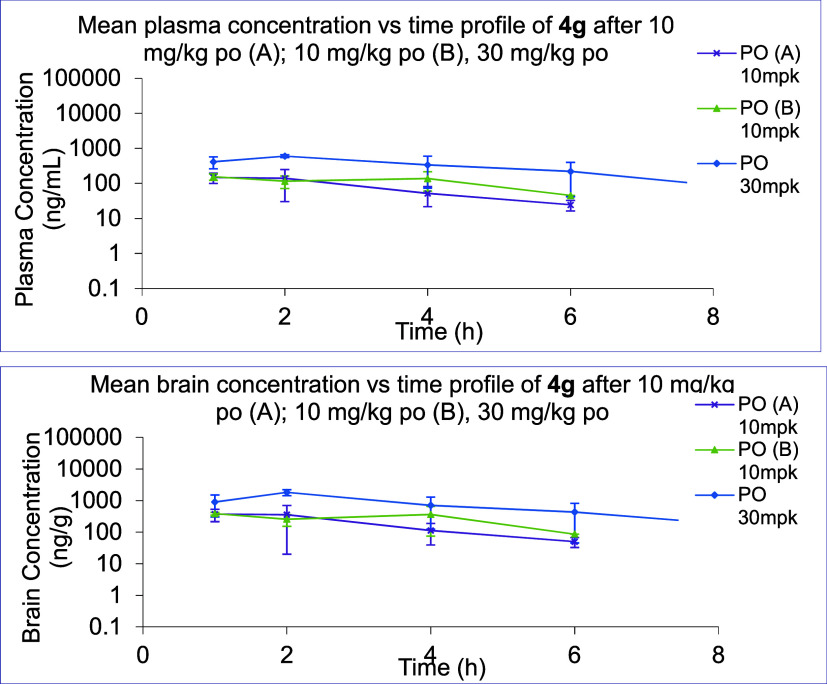
Plasma and brain pharmacokinetic profile of **4g**.

Given their low aqueous solubility, we elected to evaluate only **4g** for oral bioavailability and brain penetration in male C57BL/6 mice, the same strain employed in target engagement (*vide infra*). At a dose of 10 mg/kg **4g** has moderate oral bioavailability in mice (36%) and good brain penetration, with a brain-to-plasma ratio of 1.5:1 (Figure 2 and Table 5). In Figure 2, data is shown for two different experiments at a dose of 10 mg/kg to ensure that the low aqueous solubility of **4g** provided reproducible results. Similar bioavailability and brain penetration were obtained at a dose of 30 mg/kg, however at 100 mg/kg, the profile was poor, likely due to solubility-limited absorption (data not shown for 100 mg/kg).

**Table 5:** **4g** Peripheral Oral Pharmacokinetic Summary

	1 mg/kg IV	10 mg/kg	30 mg/kg
*T*_1/2_ (h)	0.68	1.38	1.81
*T*_max_ (h)		1.5	1
Cl_obs (mL/min/kg)	52.5		
plasma *C*_max_ (ng/mL)	524	289	679
AUC_last_ (h*ng/mL)	324	1019	3397
AUC_inf_ (h*ng/mL)	329	1043	2739
MRT_inf_ obs (h)	0.82	2.46	3.08
% *F*		32	36

**Figure 3:**
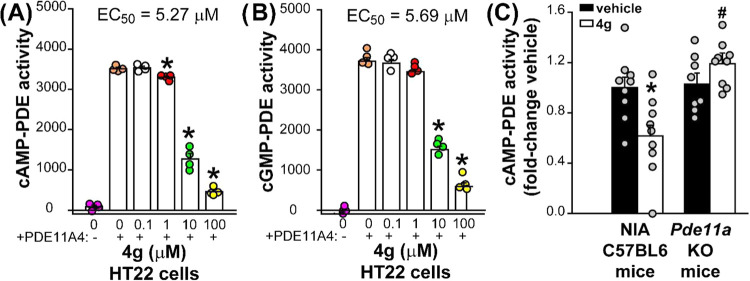
Cellular activity and *in vivo* target engagement at PDE11A4. In mouse HT22 cells transfected with PDE11A4 (+), **4g** reduced both (A) cAMP-PDE activity and (B) cGMP-PDE activity (*n* = 4 biological replicates/group) without changing PDE11A4 protein expression (Table 6). (C) In NIA C57BL6 mice, but not *Pde11a* KO mice, orally dosed **4g** significantly reduced cAMP-PDE activity in the hypothalamus relative to vehicle without affecting PDE11A4 protein expression (see Table 6). Post hoc: *vs PDE11A4+/NIA–vehicle, *p* = 0.003; #*vs* NIA C57BL6–**4g**, *p* < 0.05–0.0002.

Given its overall promising profile, we evaluated **4g** in a cell-based model that mimics age-related abnormalities in PDE11A4 function.^9^ Mouse HT22 cells that do not endogenously express PDE11A4 are transfected to express either green fluorescent protein (GFP) as a negative control or mouse PDE11A4, with the difference between vehicle-treated groups representing the PDE11A4-mediated catalytic activity (*n* = 4 biological replicates per treatment group). **4g** reduced both cAMP-PDE activity (Figure 3A; failed equal variance, analysis of variance (ANOVA) on Ranks: H(5) = 21.77, *p* = 0.0006) and cGMP-PDE activity (Figure 3B; F(5,23) = 451.33, *p* < 0.0001) in a concentration-dependent manner. This loss of PDE activity is not due to an inhibitor-induced loss of PDE11A4 protein expression (Table 6; failed normality, ANOVA on Ranks: H(4) = 0.96, *p* = 0.916). The EC_50_ value for **4g** is comparable to **1** (cAMP EC_50_ 2.5 μM) and demonstrates similar inhibition of both cyclic nucleotide substrates.^9^

To establish target engagement in the brain, **4g** was evaluated in an exploratory *in vivo* study. While there was concern regarding the low free fraction in brain, activity in the cell-based assay was favorable compared to existing standards.^9^ Based on the pharmacokinetic studies described above, we employed a dose of 30 mg/kg to achieve an expected total brain exposure of ∼5.4 μM, a value that approximates the cellular EC_50_. For oral dosing, we chose a peanut butter vehicle to minimize stress on animals in the administration process.^18,19^ Vehicle and compound-treated groups were composed of young and old female and male NIA C57BL6 mice or *Pde11a* knockout mice (*n* = 2–3/sex/age/treatment/genotype for a total of *n* = 8–9/treatment/genotype) where each compound-treated group received a single dose of **4g**. No changes in home cage behavior were noted, unlike emetic PDE4 inhibitors such as rolipram that cause illness-related behaviors such as laying in a prone position. This behavioral observation supported the PDE4D3 selectivity noted above. Two hours after dosing with **4g** or vehicle, the point at which *C*_max_ in the brain was achieved, tissue was collected and cAMP-PDE catalytic activity was measured. We focused on cAMP-PDE activity for this *in vivo* study because two strains of *Pde11a* KO mice previously showed a significant loss of cAMP-PDE activity in the brain relative to wild-type littermates, but not a loss of cGMP-PDE activity.^5,17^ In comparison to vehicle, **4g** significantly reduced hypothalamic cAMP-PDE activity in NIA C57BL6 mice but not *Pde11a* KO mice (Figure 3C, F(1,31) = 10.38, *p* = 0.003; Post hoc: NIA-vehicle *vs* NIA–4g *P* = 0.003, KO–4g *vs* NIA–4g *p* < 0.0001). This reduction of cAMP-PDE activity by **4g** in NIA C57BL6 mice was not related to reducing PDE11A4 protein expression (Table 6; t(16) = 1.43, *p* = 0.172). This shows that the **4g**-induced inhibition of cAMP-PDE activity measured in the NIA C57BL6 mice is mediated specifically by PDE11A4.

**Table 6:** PDE11A4 Protein Expression in Cell–Based and *In Vivo* Assays^a^

*in vitro*	vehicle 1.00 ± 0.25	0.1 μM 1.13 ± 0.22	1 μM 1.08 ± 0.32	10 μM 0.89 ± 0.56	100 μM 1.23 ± 0.66
*in vivo*	NIA C57BL6 vehicle 1.00 ± 0.02	NIA C57BL6 30 mg/kg 4g 0.94 ± 0.03			

a PDE11A4/Ponceau relative optical density expressed as fold–change vehicle (mean ± SEM).

This promising exploratory experiment suggests that in spite of the low free brain fraction of **4g**, there is sufficient compound in the hypothalamus to reduce PDE11A4 activity. It provides the basis for the development of a pharmacokinetic/pharmacodynamic relationship and an efficacy study for the treatment of age-related memory disorder.

## Discussion and Conclusions

This research evaluated five heterocyclic amide isosteres and a nitrile as replacements for the metabolically labile diethylamide in **1**. Our results uncovered distinct features in each of these with respect to substituent effects on potency and PDE selectivity when compared to amide **1**. The data show that three different 3,4–diazoles, **4g**, **8f**, and **8g** provide PDE11A4 inhibitors with potency comparable to diethylamide **1**. These three diazoles display notable differences in PDE selectivity, and only **4g** showed comparable PDE selectivity to **1** to merit further exploration. This PDE11A4 inhibitor has *in vitro* and cellular potency comparable to **1**. Pharmaceutical property evaluation enabled selection of **4g** as a tool compound to test PDE11A4 target engagement in the hypothalamus. Unlike **1**, **4g** has excellent microsomal stability that provided sufficient oral bioavailability and good brain penetration to be employed as a tool compound for *in vivo* PDE11A4 target engagement in the hypothalamus. In spite of the low free fraction in a brain protein binding model, a single 30 mg/kg dose of **4g** demonstrated significant inhibition of PDE11A4 in an appropriate target tissue using a cyclic nucleotide biomarker, which was apparent in female and male NIA C57BL6 mice but not *Pde11a* KO mice. This experiment provides a promising basis for ongoing optimization of PDE11A4 inhibitors to explore their use in the treatment of age–related memory disorders. Results of these investigations will be reported in due course.

## Experimental Section

### Compound Characterization

All reagents and solvents were used as received from commercial suppliers. All reactions were dried with sodium sulfate and carried out under a nitrogen atmosphere unless otherwise stated. Compounds were analyzed using a CEM mini LC system with a Restek–C18 5 μm column (150 mm × 4.6 mm, 80% acetonitrile/water isocratic gradient over 6 min with UV detection at 254 nm). Thin layer chromatography was done on silica gel G plates with UV detection. All of the reported yields are for isolated products and compounds were purified by automated flash chromatography (Teledyne Isco Rf200 +). Proton NMR spectra were obtained at 400 MHz in CDCl_3_ unless otherwise stated. All compounds in Table 1 were at least 95% pure by HPLC and had proton NMR spectra consistent with structure.

### General Procedure for Synthesis of 3,4–Thiadiazoles **4a**–**h**

Carboxylic acids **3** were synthesized as previously described.^9^

To a solution of pyrazolopyrimidine carboxylic acid **3** (1 equiv) in DMF was added HATU (1.2 equiv) and corresponding hydrazide (1.2 equiv) respectively. Diisopropylethylamine (DIPEA) (2 equiv) was added dropwise to the reaction mixture and stirred at room temperature overnight. The reaction was quenched by pouring it into brine and was extracted with three portions of methylene chloride. The organic extract was dried and evaporated. This crude material was used for the next step without purification.

Tetrahydrofuran was added to dissolve crude hydrazine amide (1 equiv) and to this mixture Lawesson’s reagent (2 equiv) was added and refluxed for 5 h. Upon completion, the reaction mixture was poured in NaHCO_3_ solution and extracted with three portions of ethyl acetate. The organic extract was washed with water and brine then dried and evaporated. The mixture was purified by flash chromatography eluting with hexane:ethyl acetate to provide the desired compound. 65–75% yield

**4a**^1^H NMR (400 MHz, CDCl_3_) δ 8.21–8.18 (d, *J* = 8 Hz, 2H), 7.67 (s, 1H), 7.63 (s, 1H), 7.59–7.55 (t, *J* = 11 Hz, 2H), 7.41–7.37 (t, *J* = 7 Hz, 1H), 6.79 (s, 1H), 4.87–4.81 (q, *J* = 7 Hz, 2H), 2.99 (s, 3H), 2.73 (s, 1H), 1.56–1.52 (t, *J* = 7 Hz, 3H).

**4b**^1^H NMR (400 MHz, CDCl_3_) δ 8.19–8.17 (d, *J* = 8 Hz, 2H), 7.66 (s, 1H), 7.62–7.61 (d, *J* = 1.8 Hz, 1H), 7.57–7.53 (t, *J* = 8 Hz, 2H), 7.39–7.38 (t, *J* = 7.8 Hz, 1H), 6.79–6.78 (d, *J* = 1.8 Hz, 1H), 4.85–4.80 (q, *J* = 7 Hz, 2H), 2.73 (s, 3H), 1.61–1.57 (t, *J* = 7.6 Hz, 3H), 1.55–1.51 (t, *J* = 7.2 Hz, 3H).

**4c**^1^H NMR (400 MHz, CDCl_3_) δ 8.84 (s, 1H), 8.20–8.16 (m, 2H), 7.91 (s, 1H), 7.65 (d, *J* = 1.9 Hz, 1H), 7.29–7.25 (m, 2H), 6.85–6.84 (d, *J* = 2.0 Hz, 1H), 4.82–4.77 (q, *J* = 7 Hz, 2H), 2.98 (s, 3H), 1.59–1.52 (t, *J* = 7 Hz, 3H).

**4d**^1^H NMR (400 MHz, CDCl_3_) δ 8.91 (s, 1H), 8.23–8.20 (m, 2H), 7.97 (s, 1H), 7.68–7.68 (d, *J* = 2 Hz, 1H), 7.31–7.28 (t, *J* = 8.4 Hz, 2H), 6.88–6.88 (d, *J* = 1.6 Hz, 1H), 4.84–4.82 (q, *J* = 7.2 Hz, 2H), 3.38–3.33 (q, *J* = 7.6 Hz, 2H), 1.63–1.54 (m, 6H).

**4e**^1^H NMR (400 MHz, CDCl_3_) δ 8.13–8.09 (m, 2H), 7.63 (s, 1H), 7.60–7.59 (d, *J* = 2 Hz, 1H), 7.24–7.20 (m, 2H), 6.76–6.75 (d, *J* = 2.0 Hz, 1H), 4.79–4.74 (q, *J* = 6.8 Hz, 2H), 2.96 (s, 3H), 2.69 (s, 3H), 1.52–1.48 (t, *J* = 7.2 Hz, 3H).

**4f**^1^H NMR (400 MHz, CDCl_3_) δ 8.23–8.20 (m, 2H), 7.90–7.76 (m, 2H), 7.65–7.60 (m, 3H), 7.50–7.47 (m, 1H), 6.88–6.88 (d, *J* = 2 Hz, 1H), 4.39 (s, 3H), 3.00 (s, 3H).

**4g**^1^H NMR (400 MHz, CDCl_3_) δ 9.43 (s, 1H), 8.21–8.18 (m, 2H), 7.32 (s, 1H), 7.65–7.65 (d, *J* = 2 Hz, 1H), 7.30–7.26 (m, 2H), 6.86–6.85 (d, *J* = 1.6 Hz, 1H), 4.40 (s, 3H), 2.70 (s, 3H).

**4h**^1^H NMR (400 MHz, CDCl_3_) δ 9.43 (s, 1H), 8.18–8.14 (m, 2H), 7.73 (s, 1H), 7.67–7.66 (d, *J* = 2 Hz, 1H), 7.31–7.26 (m, 2H), 6.83–6.83 (d, *J* = 2.0 Hz, 1H), 4.87–4.81 (q, *J* = 7.2 Hz, 2H), 2.71 (s, 3H), 1.56–1.53 (t, *J* = 7.2 Hz, 3H).

### General Procedure for Synthesis of **5a**–**i**

POCl_3_ (5 mL) was added to mixture of the appropriate carboxylic acid **3** (1 equiv) and acetyl– or propiono– hydrazide (5 equiv). The reaction mixture was refluxed for three hours, then poured into ice water. The solution was neutralized with aqueous 1N NaOH solution (pH > 9) and extracted with three portions of ethyl acetate. The combined organic extract was washed with NaHCO_3_ solution, water and dried. Purification by flash chromatography eluting with hexane:ethyl acetate provided pure products in 25–45% yield.

**5a**^1^H NMR (400 MHz, CDCl_3_) δ 8.18 (s, 1H), 8.10–8.09 (m, 2H), 8.00–7.73 (t, *J* = 53.6 Hz, 1H), 7.62–7.56 (m, 3H), 7.49–7.45 (m, 1H), 6.91–6.90 (d, *J* = 2 Hz, 1H), 4.81–4.75 (q, *J* = 7.2 Hz, 2H), 2.78 (s, 3H), 1.50–1.46 (t, *J* = 7.2 Hz, 3H).

**5b**^1^H NMR (400 MHz, CDCl_3_) δ 8.19–8.16 (m, 2H), 8.06 (s, 1H), 7.65–7.64 (d, *J* = 2 Hz, 1H), 7.59–7.55 (m, 2H), 7.42–7.39 (m, 1H), 6.88–6.88 (d, *J* = 2 Hz, 1H), 4.87–4.81 (q, *J* = 7.2 Hz, 2H), 2.96 (s, 3H), 2.79 (s, 3H), 1.55–1.51 (t, *J* = 7.2 Hz, 3H).

**5c**^1^H NMR (400 MHz, CDCl_3_) δ 8.17–8.15 (m, 2H), 8.06 (s, 1H), 7.63–7.63 (d, *J* = 2 Hz, 1H), 7.58–7.54 (m, 2H), 7.41–7.37 (m, 1H), 6.87–6.87 (d, *J* = 2 Hz, 1H), 4.85–4.79 (q, *J* = 7.2 Hz, 2H), 3.13–3.09 (t, *J* = 7.6 Hz, 3H), 2.94 (s, 3H), 1.59–1.50 (m, 6H).

**5d**^1^H NMR (400 MHz, CDCl_3_) δ 8.82 (s, 1H), 8.21–8.17 (m, 2H), 8.14 (s, 1H), 7.67–7.66 (d, *J* = 2 Hz, 1H), 7.31–7.27 (m, 2H), 6.91–6.91 (d, *J* = 1.2 Hz, 1H), 4.85–4.80 (q, *J* = 7.2 Hz, 2H), 2.81 (s, 3H), 1.57–1.53 (t, *J* = 6.8 Hz, 3H).

**5e**^1^H NMR (400 MHz, CDCl_3_) δ 8.81 (s, 1H), 8.27 (s, 1H), 8.74–7.72 (t, *J* = 6 Hz, 1H), 7.62–7.62 (d, *J* = 2 Hz, 1H), 7.58–7.52 (m, 1H), 7.42–7.36 (m, 2H), 6.89–6.88 (d, *J* = 2 Hz, 1H), 4.75–4.70 (q, *J* = 7.2 Hz, 2H), 2.81 (s, 3H), 1.40–1.36 (t, *J* = 7.2 Hz, 3H).

**5f**^1^H NMR (400 MHz, CDCl_3_) δ 8.14–8.10 (m, 2H), 8.05 (s, 1H), 7.65–7.64 (d, *J* = 2 Hz, 1H), 7.28–7.23 (m, 2H), 6.87–6.87 (d, *J* = 2.4 Hz, 1H), 4.83–4.77 (q, *J* = 7.2 Hz, 2H), 2.94 (s, 3H), 2.79 (s, 3H), 1.53–1.49 (t, *J* = 7.2 Hz, 3H).

**5g**^1^H NMR (400 MHz, CDCl_3_) δ 8.07 (s, 1H), 7.67–7.65 (m, 1H), 7.63–7.61 (m, 1H), 7.57–7.56 (d, *J* = 2 Hz, 1H), 7.54–7.50 (m, 2H), 6.88–6.87 (d, *J* = 2.0 Hz, 1H), 4.19 (s, 3H), 2.98 (s, 3H), 2.79 (s, 3H).

**5h**^1^H NMR (400 MHz, CDCl_3_) δ 8.23–8.21 (m, 2H), 8.07 (s, 1H), 7.65–7.65 (d, *J* = 2 Hz, 1H), 7.61–7.57 (m, 2H), 7.43–7.41 (m, 1H), 6.92–6.91 (d, *J* = 2 Hz, 1H), 4.41 (s, 3H), 2.97 (s, 3H), 2.80 (s, 3H).

**5i**^1^H NMR (400 MHz, CDCl_3_) δ 8.22–8.19 (m, 3H), 8.00–7.74 (t, *J* = 53.6 Hz, 1H), 7.65–7.61 (m, 3H), 7.52–7.50 (m, 1H), 6.90 (s, 1H), 4.39 (s, 3H), 2.80 (s, 3H).

### General Procedure for Synthesis of **6a**–**c**

SOCl_2_ (2 mL) was added to respective carboxylic acid **3** (1 equiv) and stirred at 80 °C for four hours at which time excess SOCl_2_ was removed by rotary evaporation. The crude acid chloride was dissolved in dioxane then, N–hydroxyacetamidine (1.3 equiv) and pyridine (5 equiv) were added. A white precipitate formed which dissolved upon heating. The reaction mixture was stirred at 90 °C overnight and poured into ice cold water. The resulting precipitate was washed with water, and dried. Purification by flash chromatography eluting with hexane:ethyl acetate provided pure products in 55–60% yield.

**6a**^1^H NMR (400 MHz, CDCl_3_) δ 8.20 (s, 1H), 7.72–7.68 (m, 1H), 7.60–7.59 (d, *J* = 5.2 Hz, 1H), 7.55–7.50 (m, 1H), 7.40–7.34 (m, 2H), 6.88 (s, 1H), 4.72–4.67 (q, *J* = 6.8 Hz, 2H), 2.95 (s, 3H), 2.63 (s, 3H), 1.36–1.33 (t, *J* = 7.2 Hz, 3H).

**6b**^1^H NMR (400 MHz, CDCl_3_) δ 8.18–8.16 (m, 2H), 8.10 (s, 1H), 7.59–7.53 (m, 3H), 7.40–7.36 (m, 1H), 6.87–6.86 (d, *J* = 2 Hz, 1H), 4.35 (s, 3H), 2.88 (s, 3H), 2.61 (s, 3H).

**6c**^1^H NMR (400 MHz, CDCl_3_) δ 8.34 (s, 1H), 8.22–8.20 (m, 2H), 8.05–7.78 (t, *J* = 54 Hz, 1H), 7.67–7.62 (m, 3H), 7.53–7.49 (m, 1H), 7.0 (s, 1H), 4.41 (s, 3H), 2.67 (s, 3H).

### Synthesis of Nitrile **11**

The appropriate carboxylic acid **3** (1 equiv) was dissolved in DMF at room temperature, followed by addition of 1.5 equiv each of ammonium chloride, HATU and triethylamine. The reaction was stirred at room temperature overnight, then poured into ice water. The mixture was extracted with two portions of ethyl acetate and the collected organic extracts were washed with aqueous 1 N HCl, 1N NaOH and brine. The organic extract was dried and concentrated to provide a crude amide that was used without further purification. This material was dissolved in POCl_3_ and refluxed for three hours. The reaction mixture was poured into ice water and extracted with three portions of ethyl acetate. The combined organic extracts were washed with brine, dried and concentrated. Purification by flash chromatography eluting with hexane:ethyl acetate provided the product as a solid in 56% yield.

^1^H NMR (400 MHz, CDCl_3_) δ 8.12–8.09 (m, 2H), 8.01 (s, 1H), 7.68–7.62 (m, 3H), 7.55–7.51 (m, 1H), 7.29–7.03 (t, *J* = 53.2 Hz, 1H), 6.90–6.90 (d, *J* = 2.0 Hz, 1H), 4.83–4.77 (q, *J* = 7.2 Hz, 2H), 1.53–1.49 (t, *J* = 7.2 Hz, 3H).

### Synthesis of **7**

To a solution of nitrile **11** (1 equiv) in DMF, NH_2_OH·HCl (1.3 equiv) and triethylamine (2 equiv) were added dropwise and stirred at 90 °C for overnight. The reaction was poured into ice cold water and the precipitate that formed was collected by filtration, and washed with water and dried. This crude intermediate was dissolved in dioxane and acetyl chloride (1.2 equiv), pyridine (1.2 equiv) were added dropwise. The reaction was stirred at 90 °C overnight. The cooled reaction mixture was poured into ice water and the precipitate was collected by filtration, washed with water and dried. Purification by flash chromatography eluting with hexane/ethyl acetate provided the desired compound in 30% net yield.

^1^H NMR (400 MHz, CDCl_3_) δ 8.31 (s, 1H), 8.23–8.21 (m, 2H), 8.01–7.74 (t, *J* = 54 Hz, 1H), 7.65–7.60 (m, 3H), 7.51–7.47 (m, 1H), 6.97–6.97 (d, *J* = 2 Hz, 1H), 4.40 (s, 3H), 2.83 (s, 3H).

### General Procedure for the Synthesis of 3,4 Oxadiazoles **8a**–**j**

Dry ethanol was added to a solution of the corresponding pyrazolopyridine ester **9** (1 equiv), followed by hydrazine hydrate (5 equiv) and the solution was refluxed for three hours. Solvent was removed by rotary evaporation to afford a hydrazide that was used without further purification. Triethyl orthoformate (3 mL) was added and the mixture was refluxed overnight then poured into ice water. The mixture was extracted with three portions of ethyl acetate, washed with water, brine and dried. Purification was achieved by flash column by using hexane and ethyl acetate as solvent. 25–45% Yield

**8a**^1^H NMR (400 MHz, CDCl_3_) δ 8.77 (s, 1H), 8.27 (s, 1H), 8.22–8.19 (m, 2H), 8.00–7.73 (t, *J* = 53.6 Hz, 1H), 7.67–7.62 (m, 3H), 7.53–7.51 (m, 1H), 6.97–6.96 (d, *J* = 2 Hz, 1H), 4.40 (s, 3H).

**8b**^1^H NMR (400 MHz, CDCl_3_) δ 8.76 (s, 1H), 8.13 (s, 1H), 7.72–7.70 (m, 1H), 7.68–7.68 (d, *J* = 2 Hz, 1H), 7.62–7.52 (m, 1H), 7.41–7.34 (m, 2H), 6.86–6.86 (d, *J* = 2 Hz, 1H), 4.72–4.70 (q, *J* = 6.8 Hz, 2H), 2.99 (s, 3H), 1.37–1.34 (t, *J* = 7.2 Hz, 3H).

**8c**^1^H NMR (400 MHz, CDCl_3_) δ 8.77 (s, 1H), 8.28 (s, 1H), 8.15–8.12 (m, 2H), 8.03–7.76 (t, *J* = 54 Hz, 1H), 7.11–7.10 (d, *J* = 2.0 Hz, 1H), 7.35–7.31 (m, 2H), 6.96–6.95 (d, *J* = 2.0 Hz, 1H), 4.86–4.81 (q, *J* = 7.2 Hz, 2H), 1.54–1.50 (t, *J* = 7.2 Hz, 3H).

**8d**^1^H NMR (400 MHz, CDCl_3_) δ 8.89 (s, 1H), 8.78 (s, 1H), 8.21 (s, 1H), 7.74–7.72 (m, 1H), 7.63–7.62 (d, *J* = 2 Hz, 1H), 7.57–7.53 (m, 1H), 7.43–7.36 (m, 2H), 6.89–6.88 (d, *J* = 2 Hz, 1H), 4.75–4.71 (q, *J* = 7.2 Hz, 2H), 1.40–1.36 (t, *J* = 7.2 Hz, 3H).

**8e**^1^H NMR (400 MHz, CDCl_3_) δ 8.82 (s, 1H), 8.76 (s, 1H), 8.20 (s, 1H), 8.19–8.15 (m, 2H), 7.66–7.65 (d, *J* = 2 Hz, 1H), 7.30–7.26 (m, 2H), 6.90–6.89 (d, *J* = 2 Hz, 1H), 4.84–4.78 (q, *J* = 7.2 Hz, 2H), 1.55–1.52 (t, *J* = 7.2 Hz, 3H).

**8f**^1^H NMR (400 MHz, CDCl_3_) δ 8.75 (s, 1H), 8.15–8.11 (m, 3H), 7.66–7.66 (d, *J* = 2 Hz, 1H), 7.29–7.25 (m, 2H), 6.88–6.88 (d, *J* = 2 Hz, 1H), 4.84–4.79 (q, *J* = 7.2 Hz, 2H), 2.96 (s, 3H), 1.54–1.51 (t, *J* = 7.2 Hz, 3H).

**8g**^1^H NMR (400 MHz, CDCl_3_) δ 8.76 (s, 1H), 8.12 (s, 1H), 7.68–7.65 (m, 1H), 7.60–7.59 (d, *J* = 2 Hz, 1H), 7.15–7.09 (m, 2H), 6.85–6.84 (d, *J* = 2 Hz, 1H), 4.70–4.65 (q, *J* = 6.8 Hz, 2H), 2.96 (s, 3H), 1.37–1.34 (t, *J* = 7.2 Hz, 3H).

**8h**^1^H NMR (400 MHz, CDCl_3_) δ 8.74 (s, 1H), 8.17–8.15 (m, 2H), 8.10 (s, 1H), 7.65–7.64 (d, *J* = 2 Hz, 1H), 7.59–7.55 (m, 2H), 7.42–7.40 (m, 1H), 6.87–6.87 (d, *J* = 2 Hz, 1H), 4.86–4.81 (q, *J* = 7.2 Hz, 2H), 2.95 (s, 3H), 1.55–1.30 (t, *J* = 7.2 Hz, 3H).

**8i**^1^H NMR (400 MHz, CDCl_3_) δ 8.76 (s, 1H), 8.20–8.16 (m, 2H), 8.14 (s, 1H), 7.69–7.68 (d, *J* = 2 Hz, 1H), 7.29–7.27 (m, 2H), 6.93–6.93 (d, *J* = 2 Hz, 1H), 4.42 (s, 3H), 2.98 (s, 3H).

**8j**^1^H NMR (400 MHz, CDCl_3_) δ 8.76 (s, 1H), 8.15 (s, 1H), 7.73–7.67 (m, 2H), 7.14–7.12 (m, 2H), 6.91–6.90 (d, *J* = 2 Hz, 1H), 4.30 (s, 3H), 2.99 (s, 3H).

### General Procedure for the Synthesis of 1,2,4 Triazoles **10a**–**d**

To a mixture of the appropriate pyrazolopyridine carboxylic acid **3** (1 equiv), HATU (1.2 equiv), and NH_4_Cl (2 equiv) in DMF, triethylamine (5 equiv) were added. The reaction stirred at room temperature overnight, then poured into ice cold water. The precipitate that formed was collected by filtration and washed with water then dried to furnish the corresponding primary amide as solid in 60–65% yield that was used without further purification.

These primary amides (1 equiv) were dissolved in 5 mL *N*,*N*–dimethylformamide dimethyl acetal (DMFDMA) and stirred at 90 °C overnight. Excess solvent was evaporated and dried under vacuum. To an acetic acid solution of this crude intermediate, hydrazine hydrate (5 equiv) was added and stirred at 95 °C for one hour. The reaction mixture was poured into ice cold water and extracted with three portions of ethyl acetate. The combined organic extracts were washed with water and brine then dried and concentrated. Purification was achieved by flash column by using hexane and ethyl acetate as solvent. 56–62% yield.

**10a**^1^H NMR (400 MHz, *d*_6_–DMSO) δ 14.73 (br s 1H), 8.89 (s, 1H), 8.13 (s, 1H), 7.81–7.77 (m, 1H), 7.65–7.58 (m, 3H), 7.50–7.46 (m, 1H), 7.04–7.03 (d, *J* = 2.0 Hz, 1H), 4.60–4.54 (q, *J* = 7.2 Hz, 2H), 2.81 (s, 3H), 1.24–1.20 (t, *J* = 7.2 Hz, 3H).

**10b**^1^H NMR (400 MHz, *d*_6_–DMSO) δ 14.72 (br s, 1H), 8.87 (s, 1H), 8.26–8.23 (m, 2H) 8.13 (s, 1H), 7.65–7.60 (m, 3H), 7.42–7.40 (m, 1H), 7.09–7.08 (d, *J* = 2.0 Hz, 1H), 4.13 (s, 3H), 2.81 (s, 3H).

**10c**^1^H NMR (400 MHz, *d*_6_–DMSO) δ 14.73 (br s, 1H), 8.87 (s, 1H), 8.27–8.24 (m, 2H), 8.14 (s, 1H), 7.61–7.60 (d, *J* = 2 Hz, 1H), 7.50–7.46 (t, *J* = 8.8 Hz, 2H), 7.09–7.08 (d, *J* = 2.0 Hz, 1H), 4.30 (s, 3H), 2.81 (s, 3H).

**10d**^1^H NMR (400 MHz, *d*_6_–DMSO) δ 14.86 (br, 1H), 8.95 (s, 1H), 8.41–8.32 (m, 2H), 8.22–8.20 (m, 2H), 7.72–7.70 (m, 2H), 7.68 (s, 1H), 7.63–7.53 (m, 1H), 7.16–7.16 (d, *J* = 2.4 Hz, 1H), 4.30 (s, 3H).

### Synthesis of **12**

Ester **9**(9) (1 equiv) was dissolved in dry THF, cooled in an ice bath, and LiBH_4_ (1 M in dry THF, 3 equiv) was added dropwise at 0 °C. The reaction mixture warmed to room temperature and was stirred overnight. It was quenched by addition to cold sodium bicarbonate solution and extracted with three portions of ethyl acetate, washed with water and brine, dried then evaporated. Purification by flash chromatography eluting with hexane:ethyl acetate provided the primary alcohol as a solid in 40% yield.

Triethylamine (1.6 equiv) and methanesulfonyl chloride (1.2 equiv) were added to a solution of this alcohol in dichloromethane at 0 °C. The reaction mixture was stirred at 0 °C for two hours, then poured into cold sodium bicarbonate solution. The biphasic mixture was diluted with additional dichloromethane then washed with water, brine and dried. The organic extract was concentrated to a white solid which was used for next step without purification.

The crude mesylate (1 equiv) was dissolved in DMF at room temperature and NaCN (2 equiv) was added. The reaction stirred at 50 °C overnight then was poured into ice water and extracted with three portions of ethyl acetate. The collected organic extract was washed with water, brine, dried then concentrated. Purification by flash chromatography eluting with hexane:ethyl acetate provided the desired product as a solid in 55% yield.

^1^H NMR (400 MHz, CDCl_3_) δ 8.20–8.18 (m, 2H), 7.92 (s, 1H), 7.65–7.61 (m, 3H), 7.51–7.47 (m, 1H), 7.31–7.09 (t, *J* = 53.6 Hz, 1H), 6.96–6.90 (d, *J* = 2.0 Hz, 1H), 4.3 (s, 5H).

### PDE Enzymatic Assays

*In vitro* PDE11A4, PDEs 3A, 4D3, 5A, 6C and 10A were carried out with the appropriate cyclic nucleotide as previously described.^9^ IC_50_ values were determined from 10 point curves and values reported are the mean of at least 3 independent experiments.

### Expanded PDE Selectivity Assays

*In vitro* PDE1A, 2A, 7B, 8A, 9A selectivity assays were carried out using the human enzymes using fluorescence polarization detection of the nucleotide product. Assays were conducted in pH 7.5 buffer (10 mM Tris), 5 mM MgCl_2_, 0.01% Brij 35, 1 mM DTT. PDE1A buffer had added 0.2 mM CaCl_2_ and 0.36 μM calmodulin. Substrate concentrations: 1A, 2A– 1 μM cGMP, 7B 0.2 μM cAMP, 9A, 0.2 μM cGMP. Reactions were carried out at room temperature for 1 h using 1 and 10 μM of **4g** and **4h**. Data (% inhibition) is reported as the average of two independent determinations. IBMX was employed as a standard inhibitor of PDEs 1A and 2A. BRL–50481 was a standard inhibitor of PDE7B.TCE 5005 was a standard PDE8A inhibitor and zaprinast was a standard inhibitor of PDE9A.

### *In Vitro* ADME Assays

#### Metabolic Stability

Microsomal stability was measured by incubating compounds at 37 °C in the presence of human or mouse liver microsomes and NADPH according to standard procedures.^19^ Aliquots were removed at 5 time points, quenched and analyzed for remaining test compound. Microsomal protein content was adjusted to give accurate rates of substrate consumption. Data was reported as compound half–life in minutes (*t*_1/2_). Analysis was performed by LC/MS/MS, and MSMS analyses use positive or negative electrospray or APCI ionization. Assay acceptance criteria are 20% for all standards and 25% for the LLOQ.

#### Aqueous Solubility

Thermodynamic aqueous solubility was measured by adding 2 mg of solid test compound to 200 μL of a pH 7.4 1 M phosphate buffer in a filter plate. The plate was incubated for 72 h at room temperature, followed by vacuum filtration and analysis of the filtrate by UV analysis or by LCMSMS following established protocols. Data was reported in micromolar concentration.

#### MDCK–MDR1

MDCK cell monolayers (Absorption Systems, Malvern, PA) were grown to confluence on collagen–coated microporous membranes in 12-well assay plates. Assay buffer consists of Hanks’ balanced salt solution containing 10 mM HEPES and 15 mM glucose at pH 7.4. The buffer in the receiver chamber contained 1% bovine serum albumin. Compounds were tested at a final concentration of 5 μM in the assay buffer. Cell monolayers were dosed on the apical side (A-B) or basolateral side (B-A) and incubated at 37 °C with 5% CO_2_ in a humidified incubator. Samples were taken from the donor and receiver chambers at 120 min. Each determination was performed in duplicate. The flux of Lucifer yellow was also measured postexperimentally for each monolayer to ensure no damage was inflicted to the monolayer during flux period. Samples were assayed on a Waters TQ LC/MS/MS using positive or negative electrospray ionization.

#### Mouse Brain Tissue Binding Assay

Brain membranes were made on the day of use from frozen C57 male mice brains (Bioreclamation; MSE-Brain-C57-M). Brains were thawed and minced in 3 volumes of Dulbeccos’s PBS (pH 7.4) at 4 °C and then polytroned with two 10 s bursts on ice. A 2 μM solution of test compound or reference compound (verapamil) was prepared in PBS from an original 10 mM compound solution in DMSO (final concentration 0.02% DMSO). Equilibrium dialysis was performed using a 96-well equilibrium dialyzer with a MW cutoff of 10K (Harvard Apparatus, Holliston, MA) and placed in dual-plate rotator set to maximum speed (Harvard Apparatus, Holliston, MA) located in a 37 °C incubator with normal atmospheric conditions. 200 μL of compound/membrane samples and receiving buffer (PBS) were added to the respective sides of the 96-well dialysis plate (in triplicate), wells were capped, and the plate was placed on the rotator in the incubator and allowed to dialyze for 17 h. 100 μL of the compound/membrane samples, in a sealed microfuge tube, were also placed in the incubator for 17 h to assess compound stability and recovery. Following dialysis, samples from both sides were recovered, 75 μL aliquots transferred to a 96 well polypropylene plate containing 225 μL of acetonitrile (with 0.1 μL propafenone as an internal MS standard)/well, centrifuged for 10 min at 2000*g*, and then 150 μL of the supernatants were assessed for compound concentration by LC/MS/MS. The fraction of the unbound compound (fu) was corrected for dilution of the brain tissue by the equation: fu_Brain_ = [1/D]/[(1/fu_measured_ – 1) + 1/D], where *D* = a dilution factor of 4.

### Mouse Pharmacokinetic Procedures

#### Animals

Male C57BL/6 mice were purchased from Si Bei Fu Laboratory Animal Technology Co., Ltd. (China). The animals were 6–8 weeks old with body weights of 20–30 g on the dosing date. The animals were housed in a 12-h light/12-h dark cycle environment. This study was approved by the Pharmaron Institutional Animal Care and Use Committee (IACUC).

#### Formulation Preparation

The IV (1 mg/kg) formulation was prepared by dissolving **4g** in an appropriate volume of 20%DMSO/40%PEG400/40% water (v/v/v) to achieve nominal concentration. The oral (10, 30, 100 mg/kg) formulation was prepared by dissolving test compounds in appropriate volume of PEG400 to achieve nominal concentration. The 1, 10, and 30 mg/kg formulations were solutions, and the 100 mg/kg formulation was a homogeneous suspension.

#### Dose Administration

All mice had *ad libitum* access to food and water. Group sizes were 3 per group. IV and 10 mg/kg administration volume: 5; 30, and 100 mg/kg, 10 mL/kg.

#### Sample Collection

Blood samples were taken at the following time points postdose: IV: 1, 2, 4, 6 h. PO: 1, 2, 4, 6 h. Brain samples were taken at the following time points postdose: 1, 2, 4, 6 h. Blood samples (∼0.03 mL/time point) were collected *via* dorsal metatarsal vein and then transferred into tubes containing EDTA-K2 as anticoagulant and then maintained on ice. Blood samples were centrifuged at 4000*g* (force) for 5 min at 4 °C to obtain plasma. The plasma samples were stored frozen at −75 ± 15 °C until analysis.

The animals were anesthetized with isoflurane by inhalation. Following blood collection, animals were perfused with phosphate buffered saline (PBS) to flush remaining blood from the circulatory system to avoid blood contamination for brain collection.

#### Brain Isolation

The skull was split into two halves by cutting upward along the sagittal suture beginning from the brain stem. The two halves of the skull were peeled away to the side and brain tissue was dissected out. All brain samples were weighted and homogenized with water by brain weight (g) to water volume (mL) ratio 1:3 before analysis. The actual concentration was the detected value multiplied by 4.

#### PK Sample Analyses

Concentrations of compound in the plasma and brain samples were analyzed using a LC-MS/MS method. WinNonlin (PhoenixTM, version 8.3) was used for pharmacokinetic calculations.

### *In Vivo* Target Engagement

Young C57BL6 mice were imported from the National Institute of Aging (NIA) colony and were then bred onsite at the University of Maryland School of Medicine (UMSOM). *Pde11a* KO mice were also bred onsite at UMSOM. All mice were group-housed 4–5/cage, with young mice tested at ∼3 months old and old mice tested at ∼18 months old. Equal numbers of female and male mice were used.

As described by others,^20^ we used peanut butter as a vehicle for oral delivery of our **4g**. Briefly, Jif brand creamy peanut butter was melted to a liquid state in a sterile beaker by stirring it on a warming plate. The liquid peanut butter was either used plain or had a weight-appropriate amount of **4g** added to yield a 30 mg/kg dose to mice based on their body weight. The plain peanut butter was then slowly poured into molds using a 3 mL syringe without a needle so as to avoid bubbles or voids. The rectangular cavities of the mold hold 100 μL of liquid and a sterile razor blade was used to scrape off any excess peanut butter that escaped the cavity. The **4g**-laden peanut butter was poured similarly into separate **4g**-designated molds. Molds were then placed onto dry ice for ∼20 min to freeze and then were stored in an ultralow freezer until time of use. On the day of testing, pellets were removed from the freezer and immediately placed on dry ice where they remained until the precise time at which a mouse was dosed. Mice were food restricted for 1 night and the next morning (Day 1), all mice were transported to a quiet room designated for *in vivo* studies. Mice were then singly housed in clean plastic cages with no bedding, allowed to habituate for 1 h, and then provided a plain peanut butter pellet for habituation. Typically mice took ∼10–60 min to eat the first pellet. At this point, ad lib food was returned to the home cage. On day 2, mice were again habituated to a plain peanut butter pellet, taking ∼7–8 min to consume the pellet. On day 3, half of the mice were provided a plain peanut butter pellet (vehicle) and the other half were given a peanut butter pellet containing a 30 mg/kg dose of **4g**. Generally, mice ate their pellets in ∼2–3 min.

Two hours after consuming their pellet, mice were moved to a second room and were killed by cervical dislocation. Tissue was harvested fresh on wet ice and then immediately frozen on dry ice and stored long-term at −80 °C. At the time of processing, tissue was homogenized in 20 mM Tris-HCl/10 mM MgCl_2_ using a tissue sonicator (output control: 7.5, duty cycle: 70, continuous). Samples were stored at −80 °C until processed.

### PDE Activity in Mammalian Cells and Tissue

HT22 cells were treated with compound and processed as described previously.^9^ Mouse brain tissue was obtained as described above. Total protein levels were quantified using the DC Protein Assay Kit (Bio-Rad, Hercules, CA) according to the manufacturer’s directions. Three μg of each sample was then processed for cGMP- and/or cAMP-PDE activity using a radiotracer assay as previously described.^9^ Briefly, samples were incubated with 35,000–45,000 disintegrations/min of [^3^H]-cAMP or [^3^H]-cGMP for 10 min. The reaction was then quenched with 0.1 M HCl and neutralized using 0.1 M Tris. Snake venom was then added to the sample and incubated for 10 min at 37 °C. Samples were then run down DEAE A–25 Sephadex columns previously equilibrated in high salt buffer (20 mM Tris-HCl, 0.1% sodium azide, and 0.5 M NaCl) and low salt buffer (20 mM Tris-HCl and 0.1% sodium azide). After washing the columns four times with 0.5 mL of low salt buffer, the eluate was mixed with 4 mL of scintillation cocktail, and then CPMs were read on a Beckman-Coulter liquid scintillation counter. Two reactions not containing any sample lysate were also taken through the assay to assess background, which was subtracted from the sample CPMs.

### Statistical Analyses for Cellular and Brain PDE11A4 Activity

All between-group analyses were performed using Sigmaplot v11.2. EC_50_ calculations were performed using the The Quest Graph online calculator (https://www.aatbio.com/tools/ic50-calculator; accessed 09/23/23). This calculator models an experimental set using a four-parameter logistic regression, using the following formula
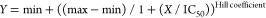
Treatment effects of the inhibitor were analyzed by ANOVA (F) or ANOVA on ranks (H) when normality and/or equal variance failed. *Post hoc* tests were conducted using Student–Newman–Keuls method and significance was defined as *P* < 0.05. Please note that Sigmaplot provides exact *P*-values for post hoc tests following a significant parametric ANOVA, but only yes or no to “*P* < 0.05” for post hoc tests following a significant nonparametric ANOVA. Data are graphed mean ± standard error of the mean (SEM).

## Supporting Information Available

400 MHz proton NMR, HPLC analysis (PDF)
SMILES strings (CSV)

## Abbreviations Used

ADME: absorption, distribution, metabolism, elimination
cAMP: cyclic adenosine monophosphate
cGMP: cyclic guanosine monophosphate
DIPEA: diisopropylethylamine
HATU: hexafluorophosphate azabenzotriazole tetramethyl uronium
MDCK1: Madin-Darby Canine kidney cells
PDE11A4: phosphodiesterase type 11A isoform 4
SAR: structure–activity relationships
