# Supplementary material for: First Demonstration of In Vivo PDE11A4 Target Engagement for Potential Treatment of Age-Related Memory Disorders
Source: J Med Chem. 2024 Sep 25;67(19):17774–84. doi: 10.1021/acs.jmedchem.4c01794 (PMC11472338; doi:10.1021/acs.jmedchem.4c01794)
Supplement: Supplementary file 1 — jm4c01794_si_001.pdf [file jm4c01794_si_001.pdf]

Supplementary Information

First Demonstration of *in vivo* PDE11A4 Target Engagement for Potential Treatment of Age-Related Memory Disorders

Shams ul Mahmood[1,6], Jeremy Eberhard[2], Charles S. Hoffman[2], Dennis Colussi[3], John Gordon[3], Wayne Childers[3], Elvis Amurrio[4], Janvi Patel[4], Michy P. Kelly[*4,5], David P. Rotella[*1,6]

[1]Department of Chemistry and Biochemistry, Montclair State University, 1 Normal Avenue, Montclair NJ 07043; [2]Biology Department, Boston College, Chestnut Hill MA, 02467; [3]Moulder Center for Drug Discovery Research, Temple University, Philadelphia PA, 19140; [4]Department of Neurobiology, University of Maryland School of Medicine, Baltimore MD, 21201; [5]Center for Research on Aging, University of Maryland, Baltimore School of Medicine, Baltimore MD, 21201; [6]Sokol Institute for Pharmaceutical Life Sciences, Montclair State University, Montclair NJ 07043

Contents | Pages

1. 400 MHz proton NMR spectra — S2-S24
2. Analytical HPLC traces — S25-S61

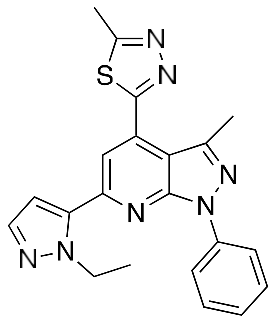

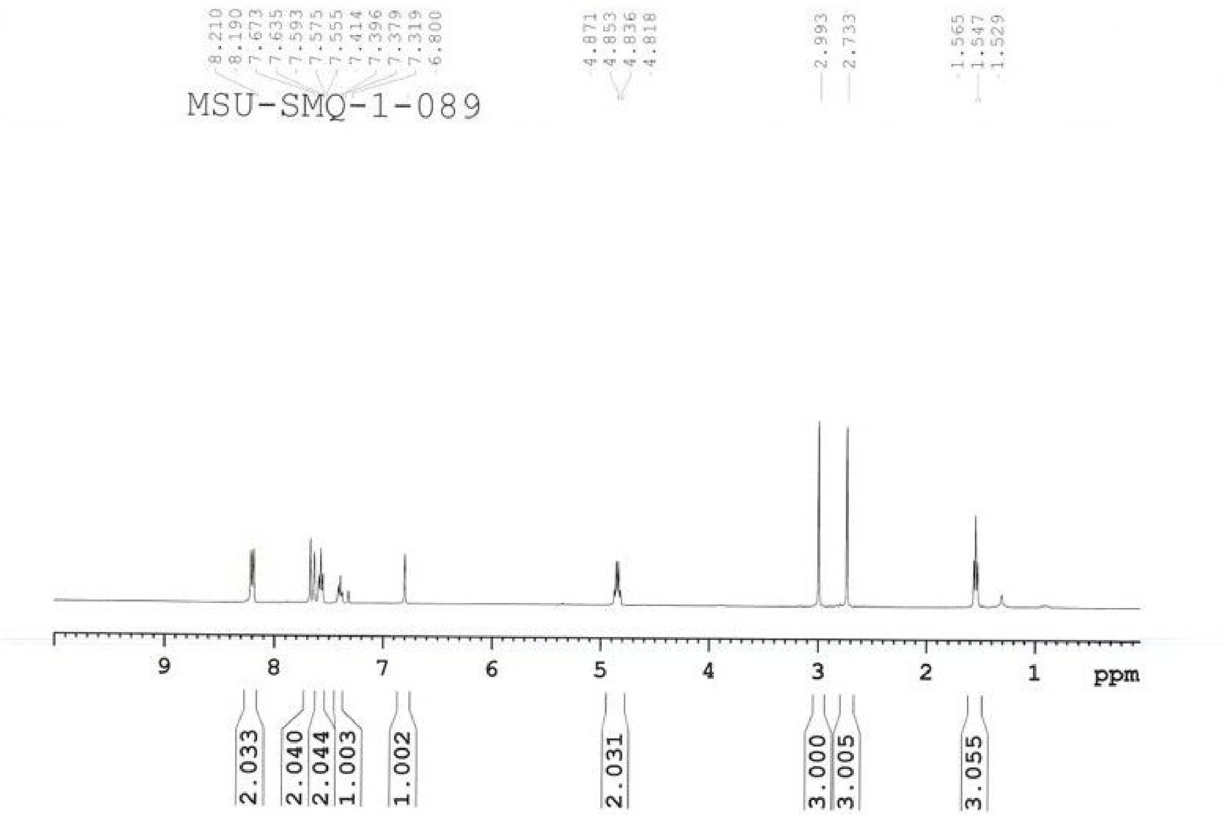
MSU-SMQ-1-089
8.210
8.190
7.673
7.635
7.593
7.575
7.555
7.414
7.396
7.379
7.319
6.800
4.871
4.853
4.836
4.818
2.993
2.733
1.565
1.547
1.529
9
8
7
6
5
4
3
2
1
ppm
2.033
2.040
2.044
1.003
1.002
2.031
3.000
3.005
3.055

4a

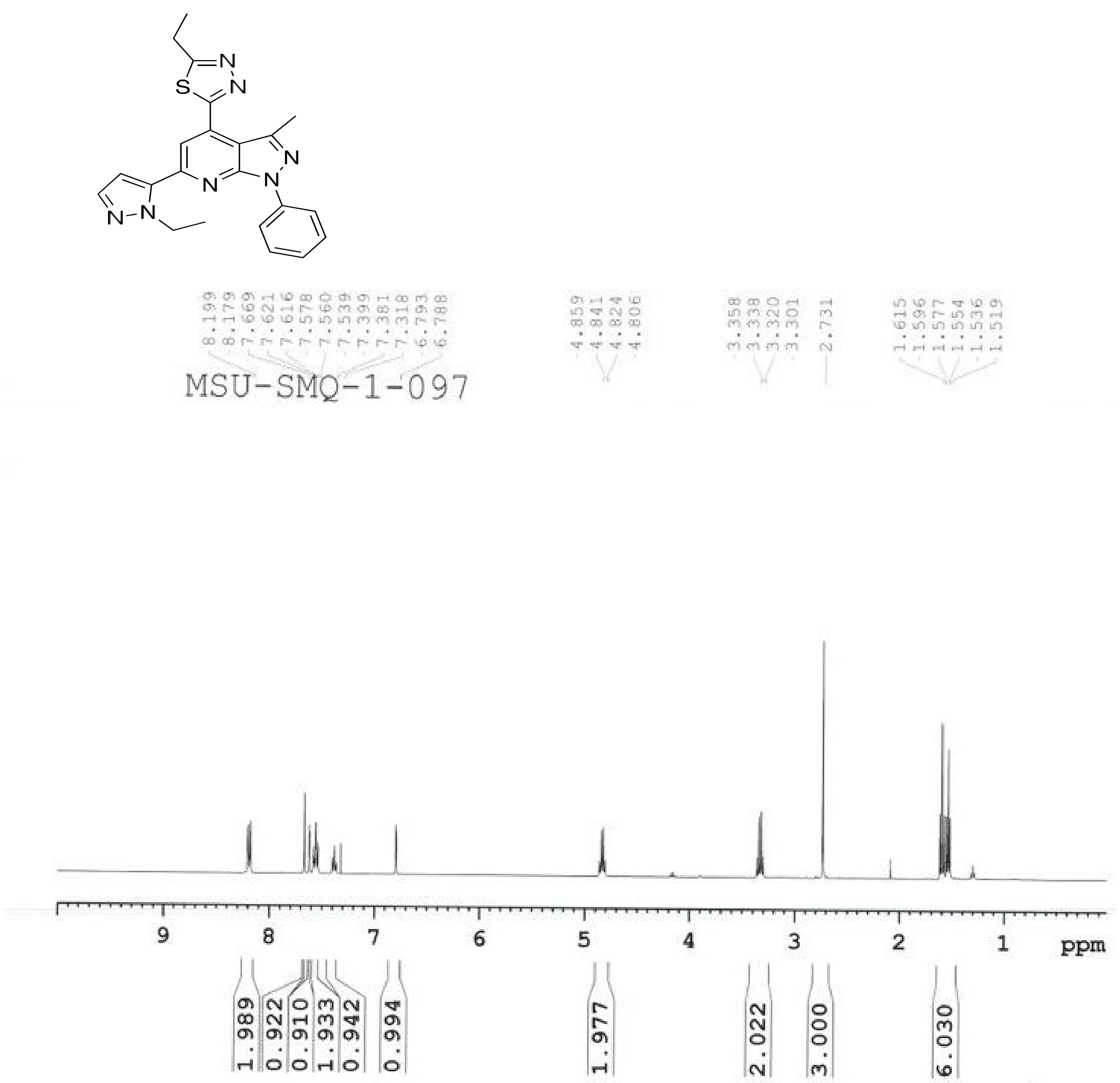

S
N
N
N
N
N
N
N
8.199
8.179
7.669
7.621
7.616
7.578
7.560
7.539
7.399
7.381
7.318
6.793
6.788
4.859
4.841
4.824
4.806
3.358
3.338
3.320
3.301
2.731
1.615
1.596
1.577
1.554
1.536
1.519
MSU-SMQ-1-097
9
8
7
6
5
4
3
2
1
ppm
1.989
0.922
0.910
1.933
0.942
0.994
1.977
2.022
3.000
6.030

4b

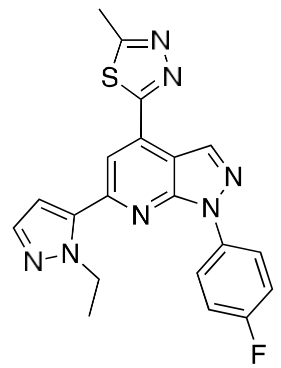
S
N
N
N
N
N
N
N
F

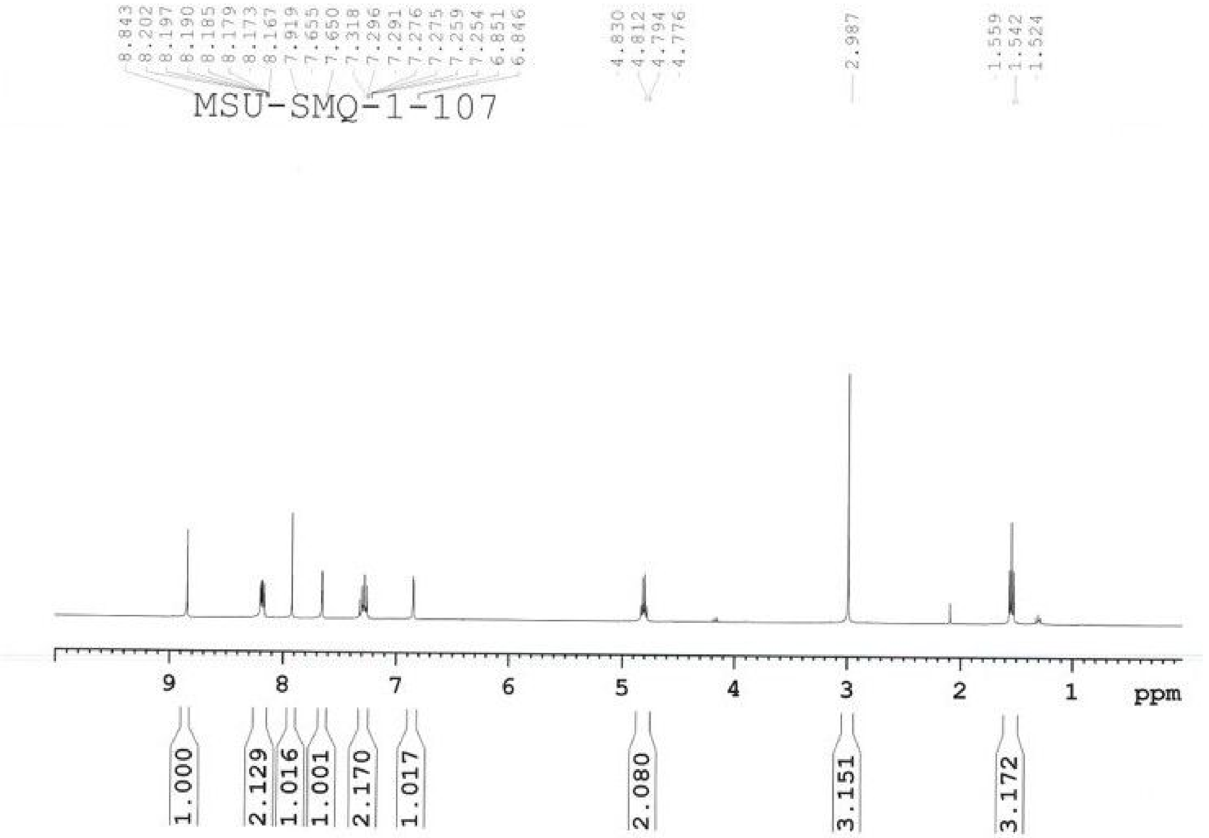
8.843
8.202
8.197
8.190
8.185
8.179
8.173
8.167
7.919
7.655
7.650
7.318
7.296
7.291
7.276
7.275
7.259
7.254
6.851
6.846
4.830
4.812
4.794
4.776
2.987
1.559
1.542
1.524
MSU-SMQ-1-107
9
8
7
6
5
4
3
2
1
ppm
1.000
2.129
1.016
1.001
2.170
1.017
2.080
3.151
3.172

4c

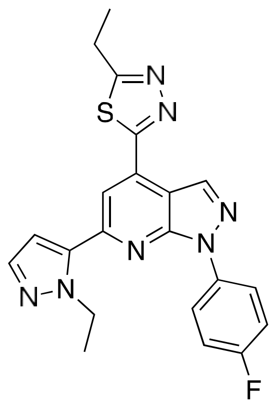
N
S
N
N
N
N
N
N
F

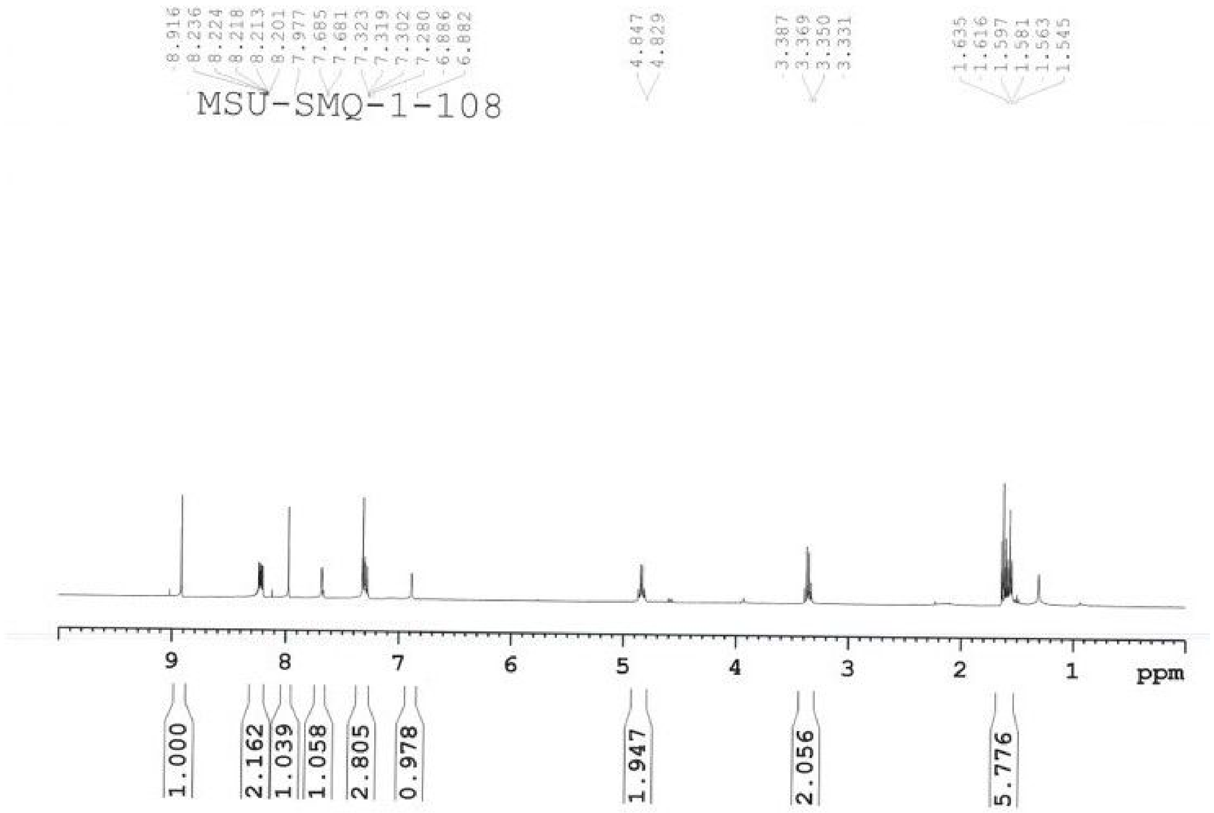
8.916
8.236
8.224
8.218
8.213
8.201
7.977
7.685
7.681
7.323
7.319
7.302
7.280
6.886
6.882
4.847
4.829
3.387
3.369
3.350
3.331
1.635
1.616
1.597
1.581
1.563
1.545
MSU-SMQ-1-108
9
8
7
6
5
4
3
2
1
ppm
1.000
2.162
1.039
1.058
2.805
0.978
1.947
2.056
5.776

4d

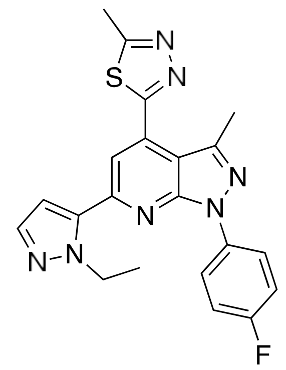

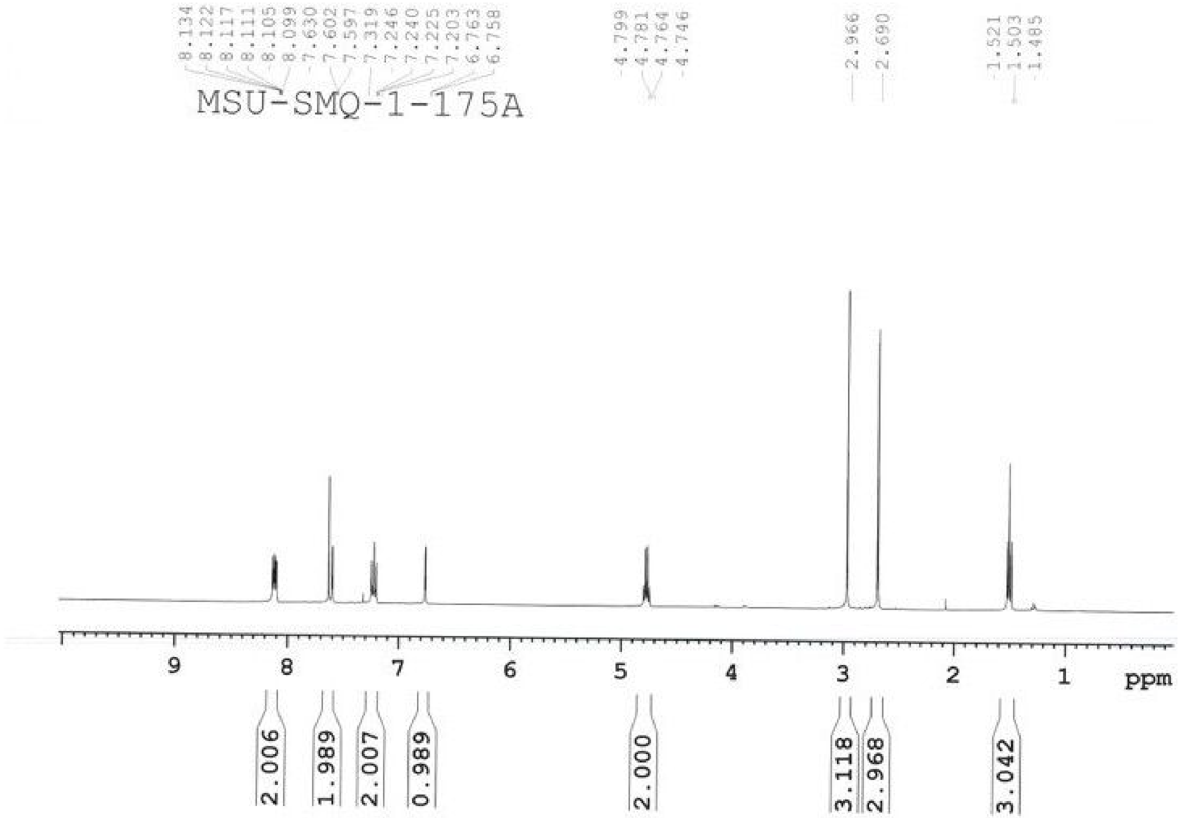

8.134
8.122
8.117
8.111
8.105
8.099
7.630
7.602
7.597
7.319
7.246
7.240
7.225
7.203
6.763
6.758
4.799
4.781
4.764
4.746
2.966
2.690
1.521
1.503
1.485
MSU-SMQ-1-175A
9
8
7
6
5
4
3
2
1
ppm
2.006
1.989
2.007
0.989
2.000
3.118
2.968
3.042

4e

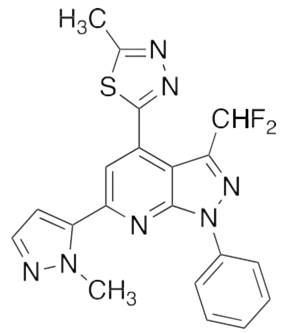

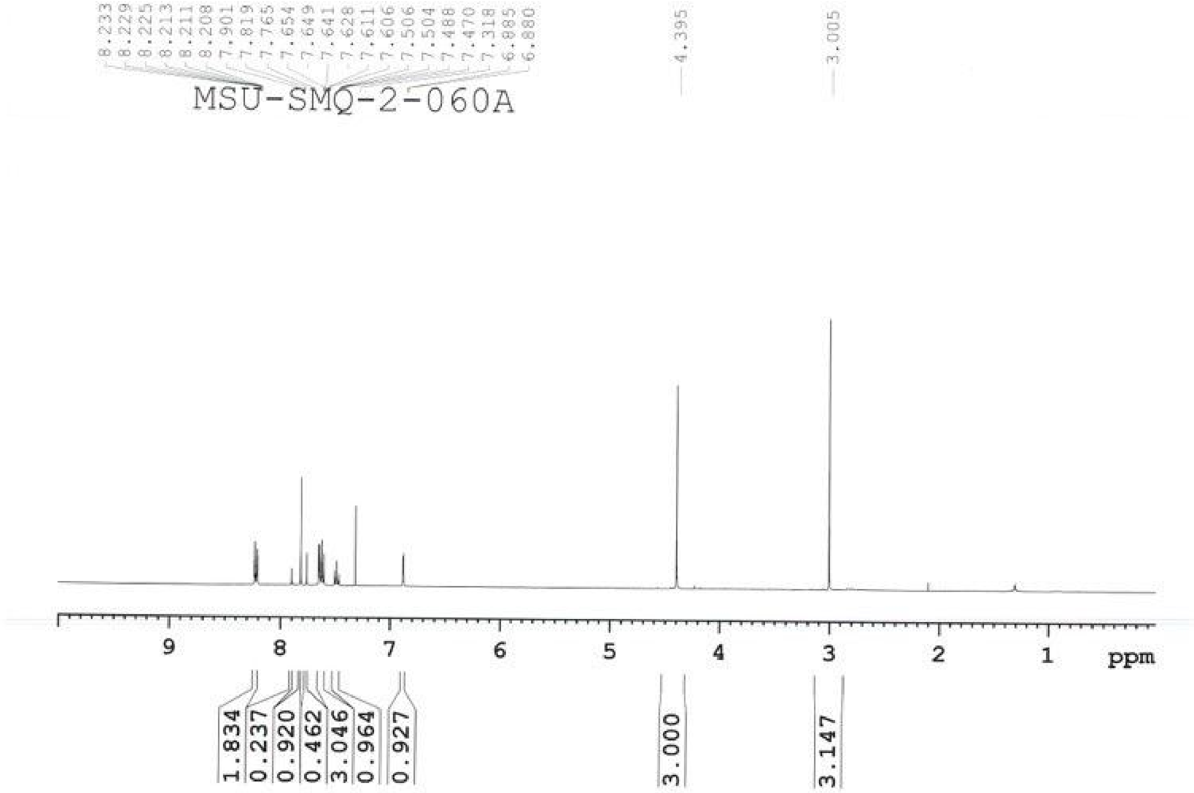

MSU-SMQ-2-060A
8.233
8.229
8.225
8.213
8.211
8.208
7.901
7.819
7.765
7.654
7.649
7.641
7.628
7.611
7.606
7.506
7.504
7.488
7.470
7.318
6.885
6.880
4.395
3.005
9
8
7
6
5
4
3
2
1
ppm
1.834
0.237
0.920
0.462
3.046
0.964
0.927
3.000
3.147

4f

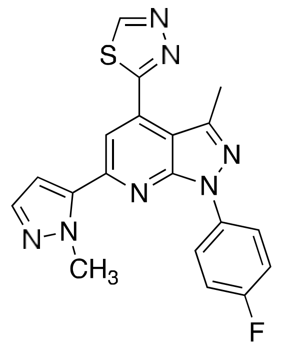
S
N
N
N
N
N
N
N
CH3
F

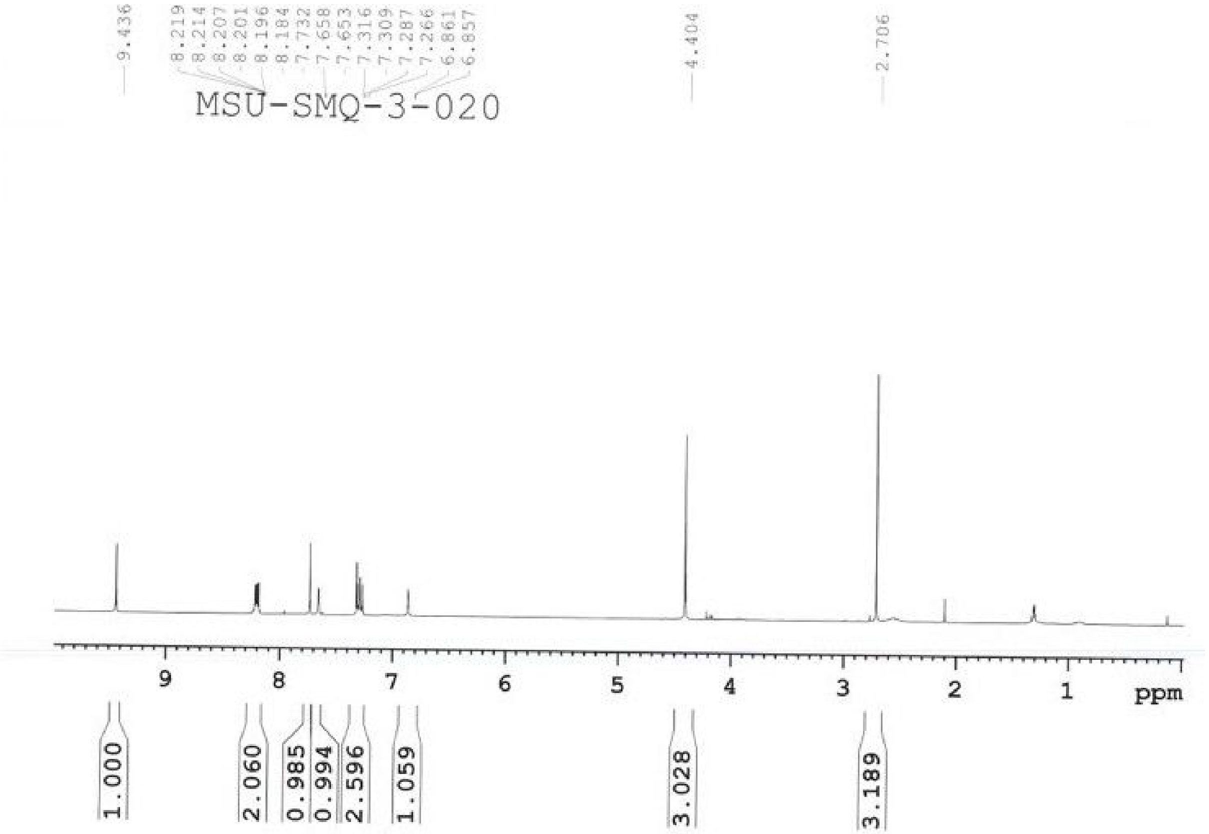
9.436
8.219
8.214
8.207
8.201
8.196
8.184
7.732
7.658
7.653
7.316
7.309
7.287
7.266
6.861
6.857
4.404
2.706
MSU-SMQ-3-020
9
8
7
6
5
4
3
2
1
ppm
1.000
2.060
0.985
0.994
2.596
1.059
3.028
3.189

4g

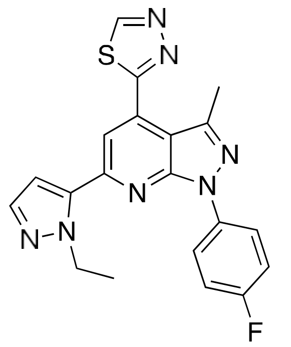

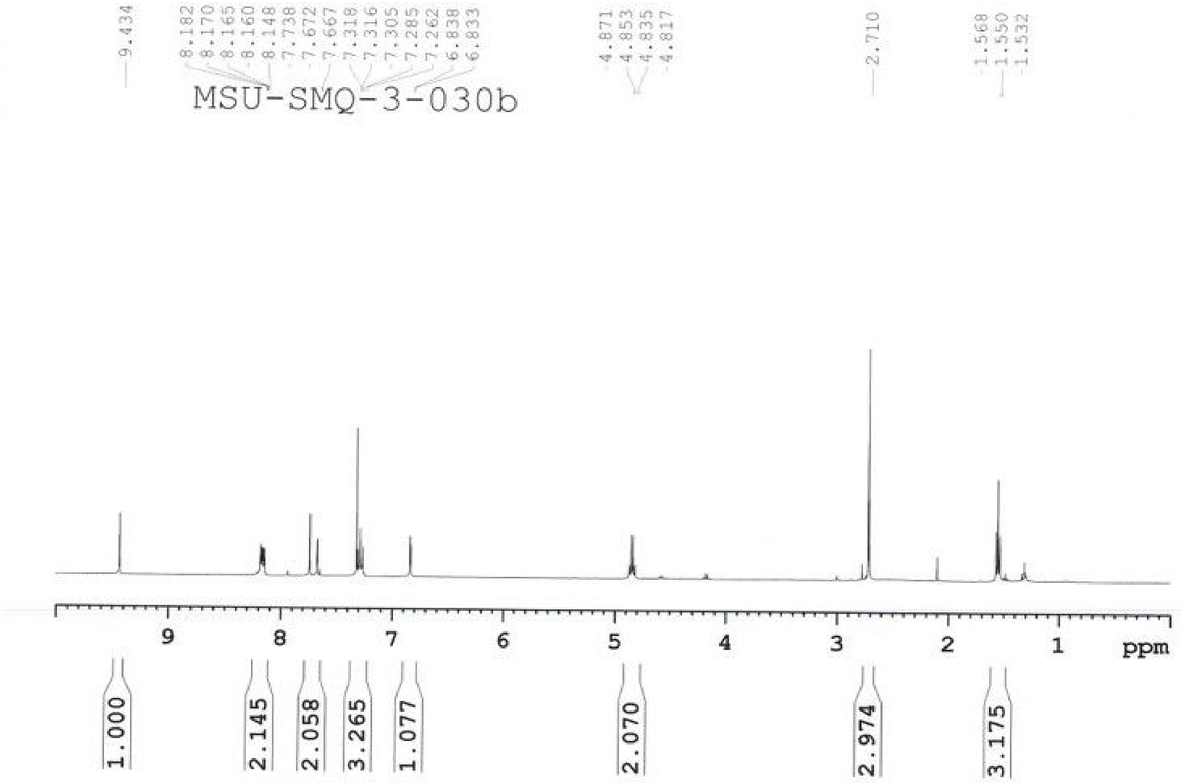
9.434
8.182
8.170
8.165
8.160
8.148
7.738
7.672
7.667
7.318
7.316
7.305
7.285
7.262
6.838
6.833
4.871
4.853
4.835
4.817
2.710
1.568
1.550
1.532
MSU-SMQ-3-030b
9 8 7 6 5 4 3 2 1 ppm
1.000
2.145
2.058
3.265
1.077
2.070
2.974
3.175

4h

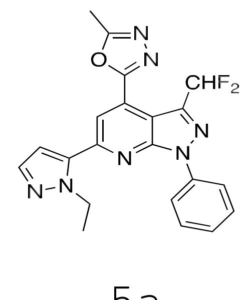
N
O
N
CHF2
N
N
N
N
N

5a

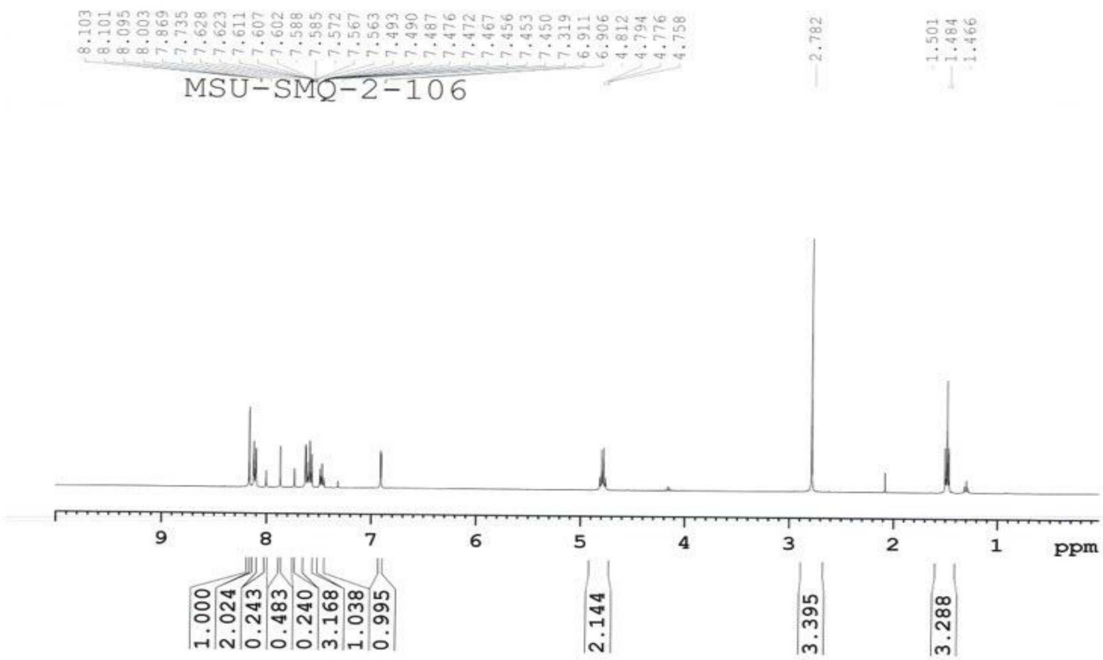
8.103
8.101
8.095
8.003
7.869
7.735
7.628
7.623
7.611
7.607
7.602
7.588
7.585
7.572
7.567
7.563
7.493
7.490
7.487
7.476
7.472
7.467
7.456
7.453
7.450
7.319
6.911
6.906
4.812
4.794
4.776
4.758
2.782
1.501
1.484
1.466
MSU-SMQ-2-106
9
8
7
6
5
4
3
2
1
ppm
1.000
2.024
0.243
0.483
0.240
3.168
1.038
0.995
2.144
3.395
3.288

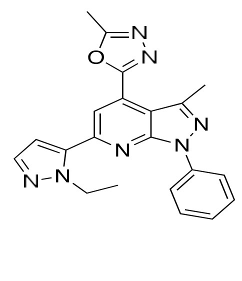
N
O
N
N
N
N
N
N

5b

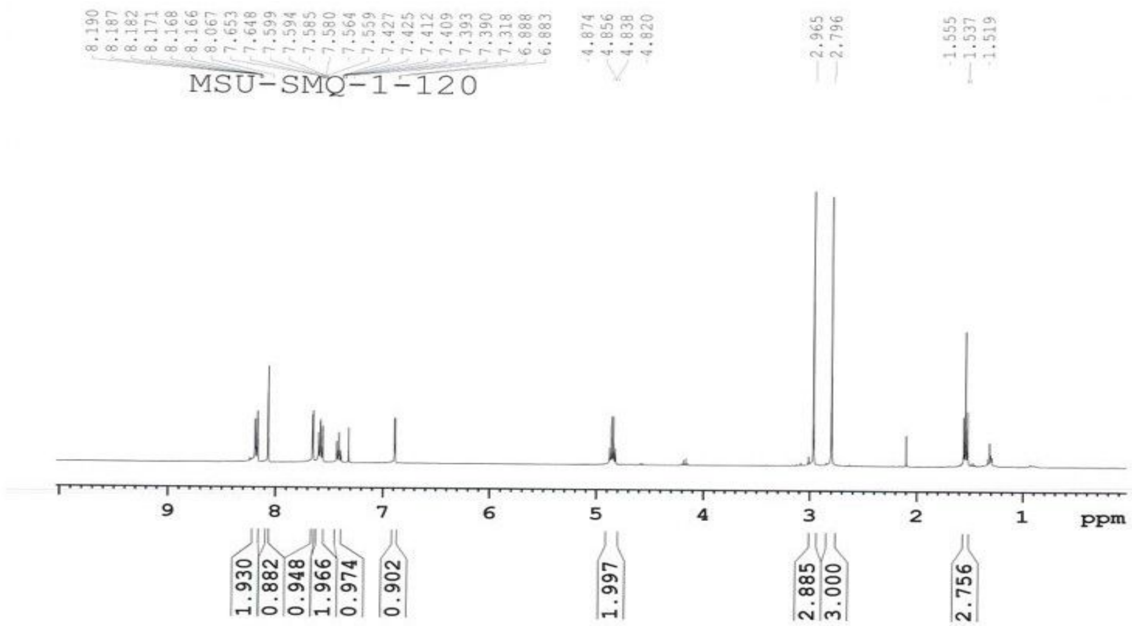
8.190
8.187
8.182
8.171
8.168
8.166
8.067
7.653
7.648
7.599
7.594
7.585
7.580
7.564
7.559
7.427
7.425
7.412
7.409
7.393
7.390
7.318
6.888
6.883
4.874
4.856
4.838
4.820
2.965
2.796
1.555
1.537
1.519
MSU-SMQ-1-120
9
8
7
6
5
4
3
2
1
ppm
1.930
0.882
0.948
1.966
0.974
0.902
1.997
2.885
3.000
2.756

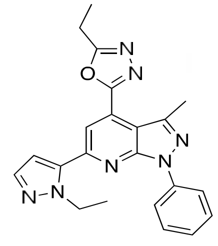

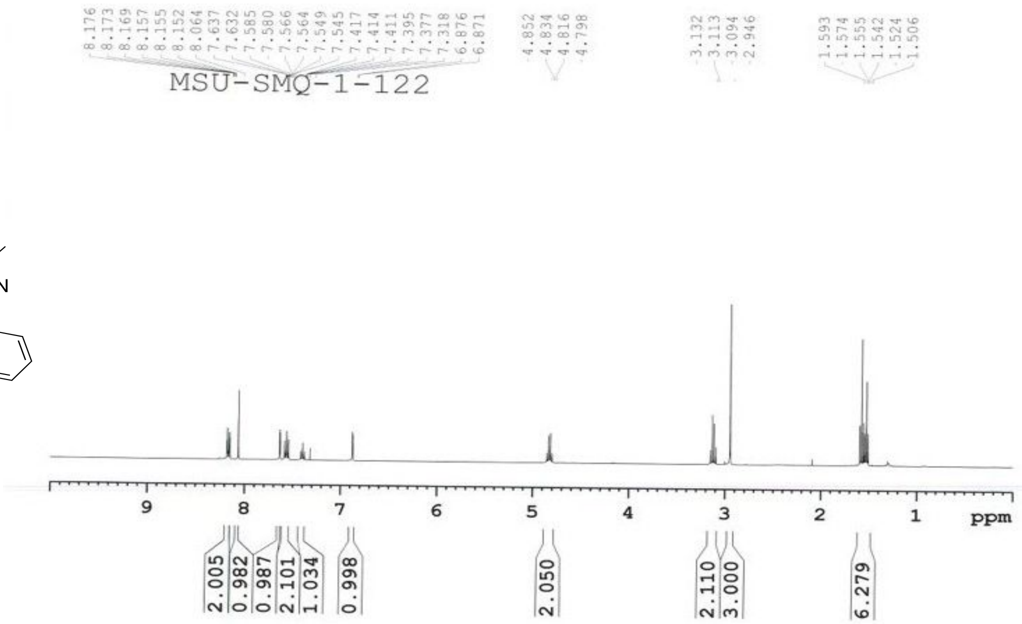
8.176 8.173 8.169 8.157 8.155 8.152 8.064 7.637 7.632 7.585 7.580 7.566 7.564 7.549 7.545 7.417 7.414 7.411 7.395 7.377 7.318 6.876 6.871
4.852 4.834 4.816 4.798
3.132 3.113 3.094 2.946
1.593 1.574 1.555 1.542 1.524 1.506
MSU-SMQ-1-122
9 8 7 6 5 4 3 2 1 ppm
2.005 0.982 0.987 2.101 1.034 0.998 2.050 2.110 3.000 6.279

5c

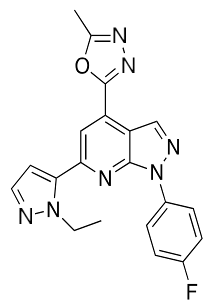

5d

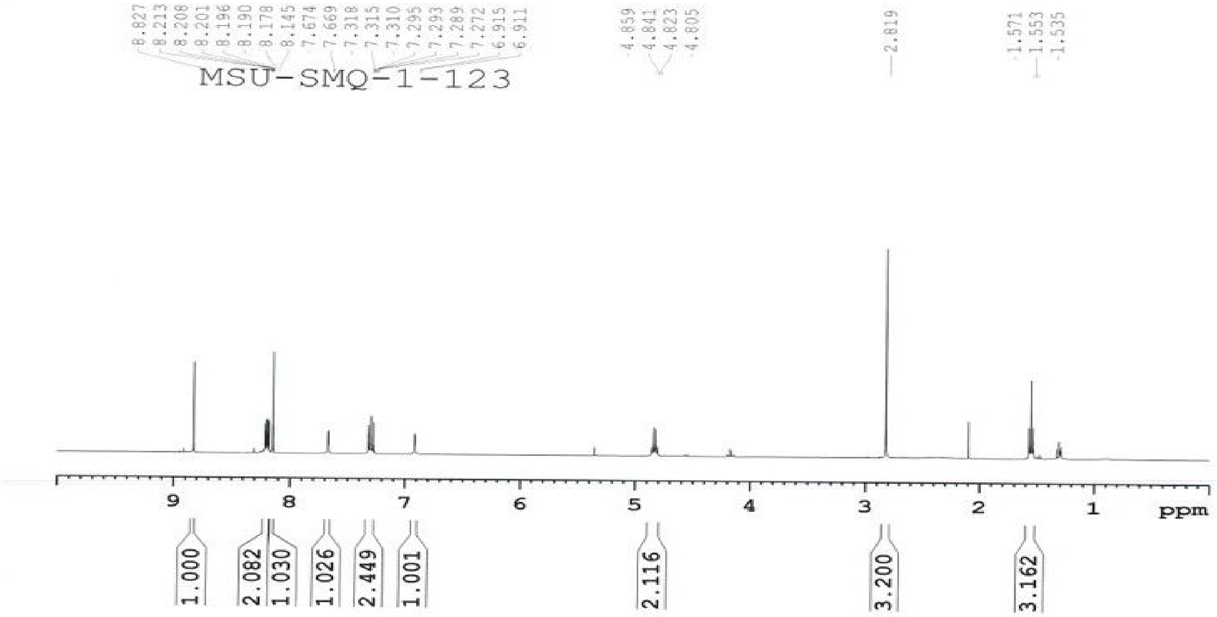
8.827 8.213 8.208 8.201 8.196 8.190 8.178 8.145 7.674 7.669 7.318 7.315 7.310 7.295 7.293 7.289 7.272 6.915 6.911
4.859 4.841 4.823 4.805
2.819
1.571 1.553 1.535
MSU-SMQ-1-123
9 8 7 6 5 4 3 2 1 ppm
1.000 2.082 1.030 1.026 2.449 1.001 2.116 3.200 3.162

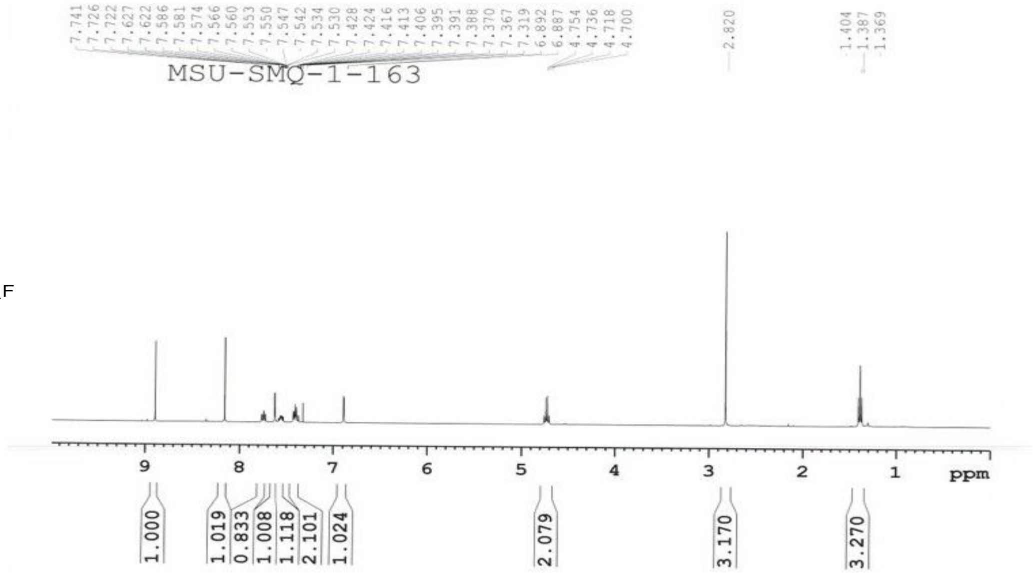

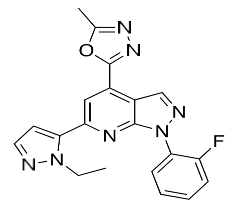

5e

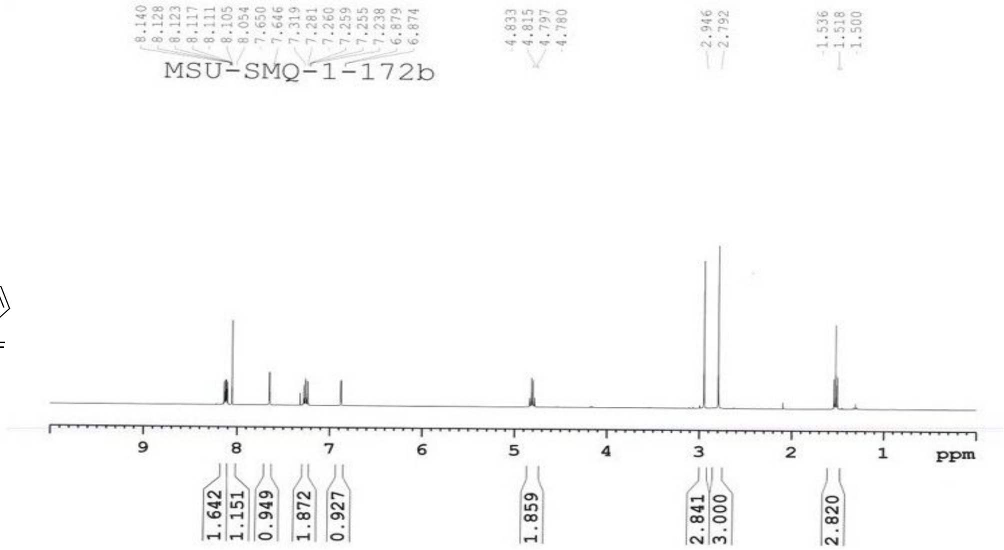

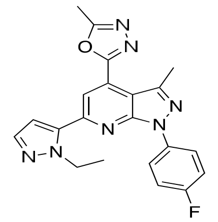

5f

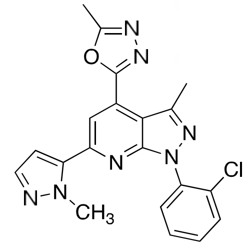

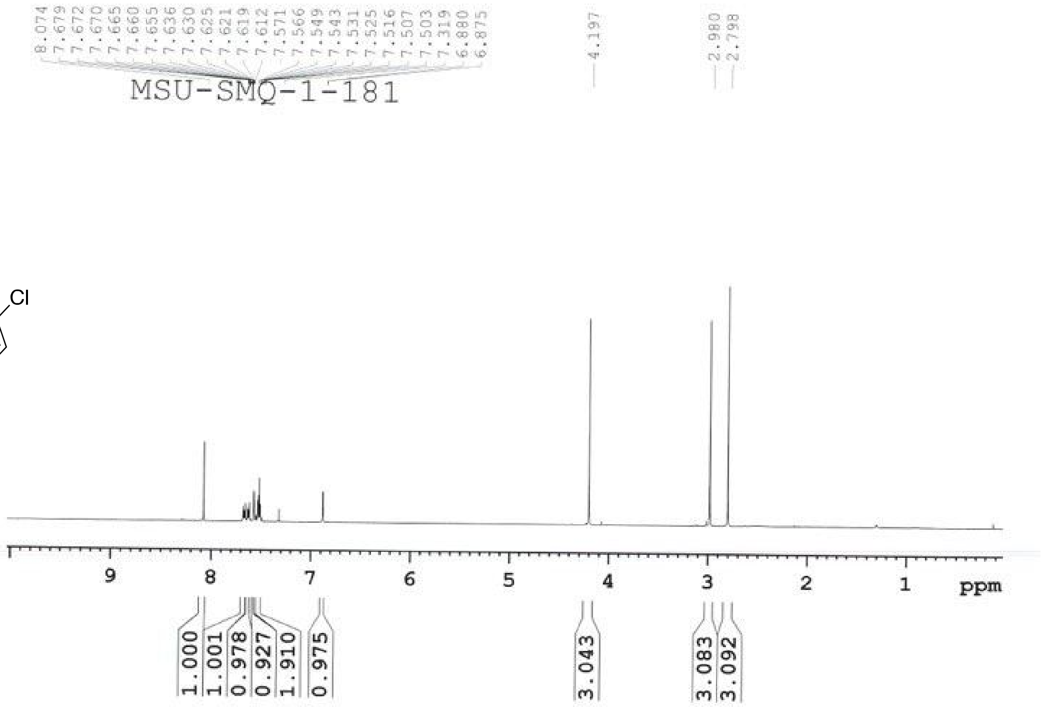
8.074 7.679 7.672 7.670 7.665 7.660 7.655 7.636 7.630 7.625 7.621 7.619 7.612 7.571 7.566 7.549 7.543 7.531 7.525 7.516 7.507 7.503 7.319 6.880 6.875
4.197
2.980 2.798
MSU-SMQ-1-181
9 8 7 6 5 4 3 2 1 ppm
1.000 1.001 0.978 0.927 1.910 0.975
3.043
3.083 3.092

5g

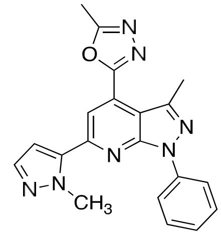

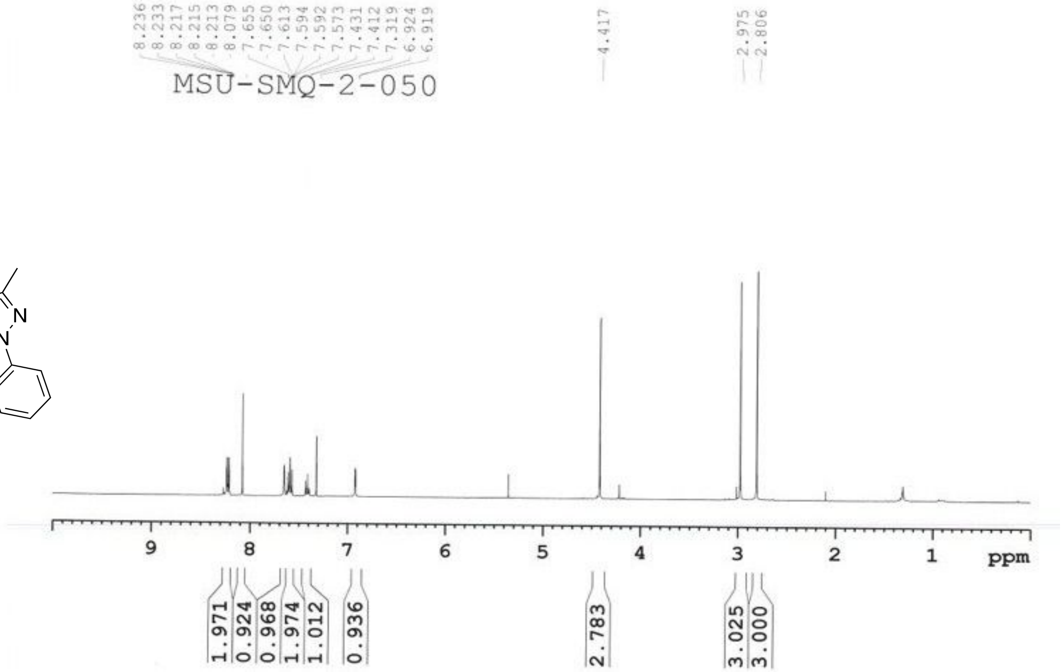
8.236 8.233 8.217 8.215 8.213 8.079 7.655 7.650 7.613 7.594 7.592 7.573 7.431 7.412 7.319 6.924 6.919
4.417
2.975 2.806
MSU-SMQ-2-050
9 8 7 6 5 4 3 2 1 ppm
1.971 0.924 0.968 1.974 1.012 0.936
2.783
3.025 3.000

5h

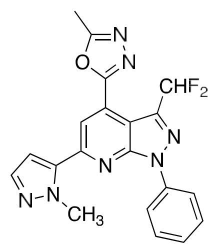
CHF2
N
O
N
N
CH3

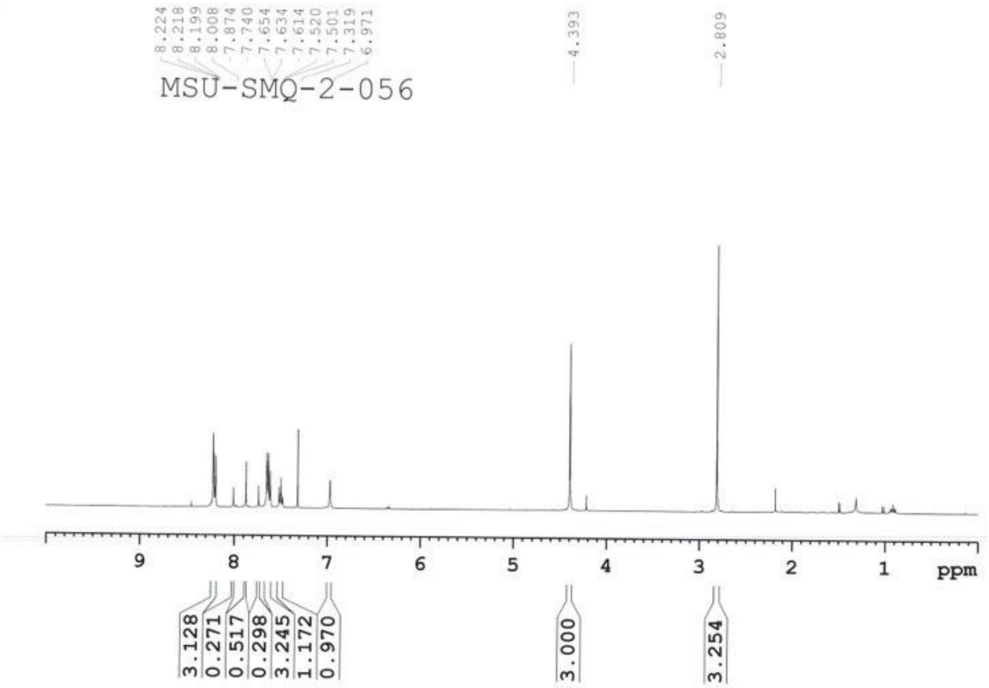
8.224
8.218
8.199
8.008
7.874
7.740
7.654
7.634
7.614
7.520
7.501
7.319
6.971
4.393
2.809
MSU-SMQ-2-056
ppm
3.128
0.271
0.517
0.298
3.245
1.172
0.970
3.000
3.254

5i

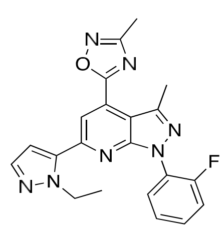
O
N
F

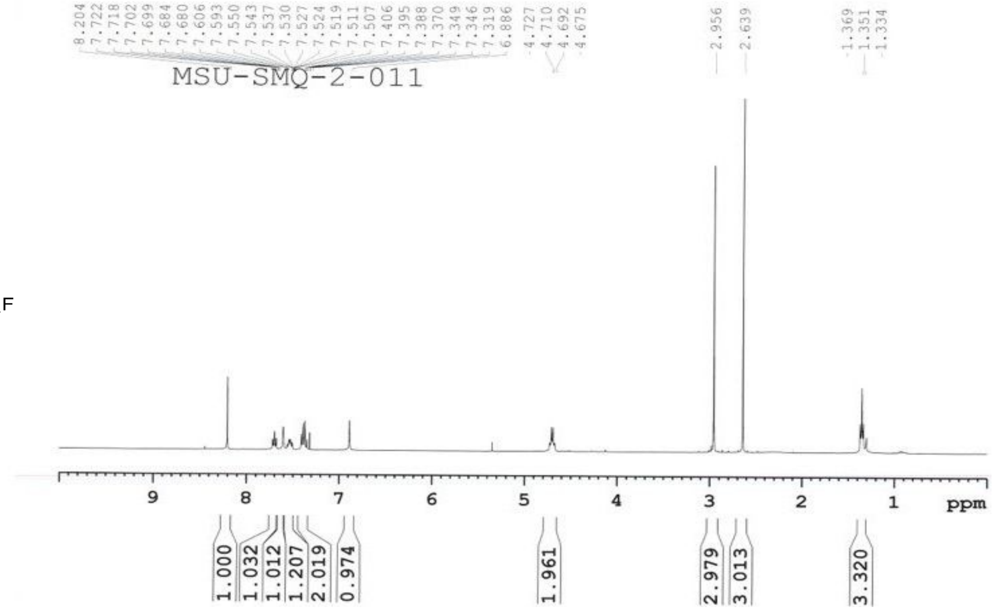
8.204
7.722
7.718
7.702
7.699
7.684
7.680
7.606
7.593
7.550
7.543
7.537
7.530
7.527
7.524
7.519
7.511
7.507
7.406
7.395
7.388
7.370
7.349
7.346
7.319
6.886
4.727
4.710
4.692
4.675
2.956
2.639
1.369
1.351
1.334
MSU-SMQ-2-011
ppm
1.000
1.032
1.012
1.207
2.019
0.974
1.961
2.979
3.013
3.320

6a

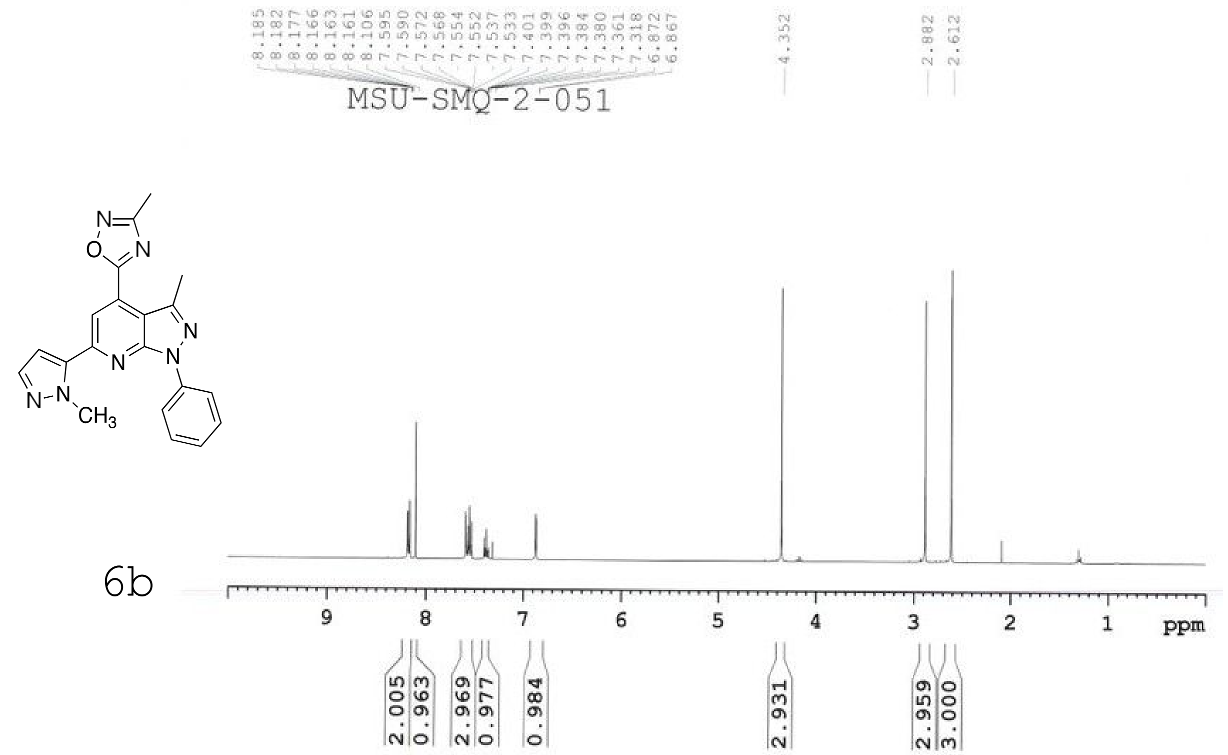
8.185
8.182
8.177
8.166
8.163
8.161
8.106
7.595
7.590
7.572
7.568
7.554
7.552
7.537
7.533
7.401
7.399
7.396
7.384
7.380
7.361
7.318
6.872
6.867
4.352
2.882
2.612
MSU-SMQ-2-051
N
O
N
N
N
N
N
N
CH_3
9
8
7
6
5
4
3
2
1
ppm
2.005
0.963
2.969
0.977
0.984
2.931
2.959
3.000

6b

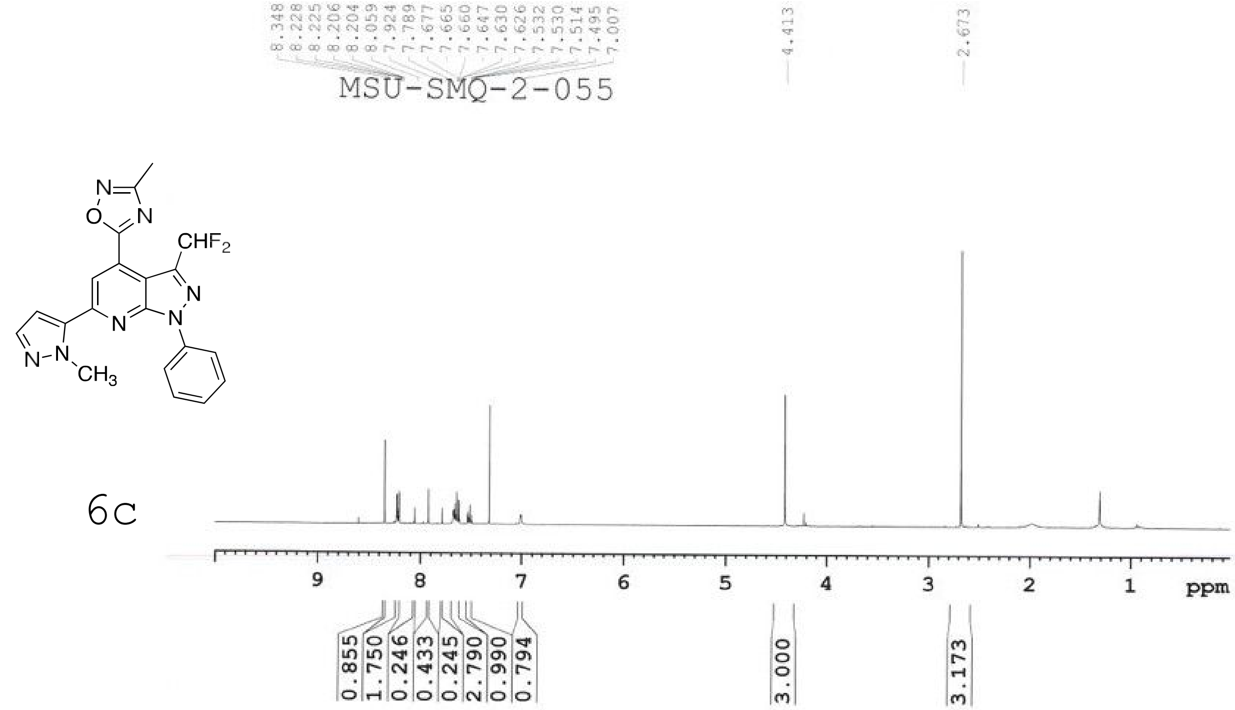
8.348
8.228
8.225
8.206
8.204
8.059
7.924
7.789
7.677
7.665
7.660
7.647
7.630
7.626
7.532
7.530
7.514
7.495
7.007
4.413
2.673
MSU-SMQ-2-055
N
O
N
CHF_2
N
N
N
N
N
CH_3
9
8
7
6
5
4
3
2
1
ppm
0.855
1.750
0.246
0.433
0.245
2.790
0.990
0.794
3.000
3.173

6c

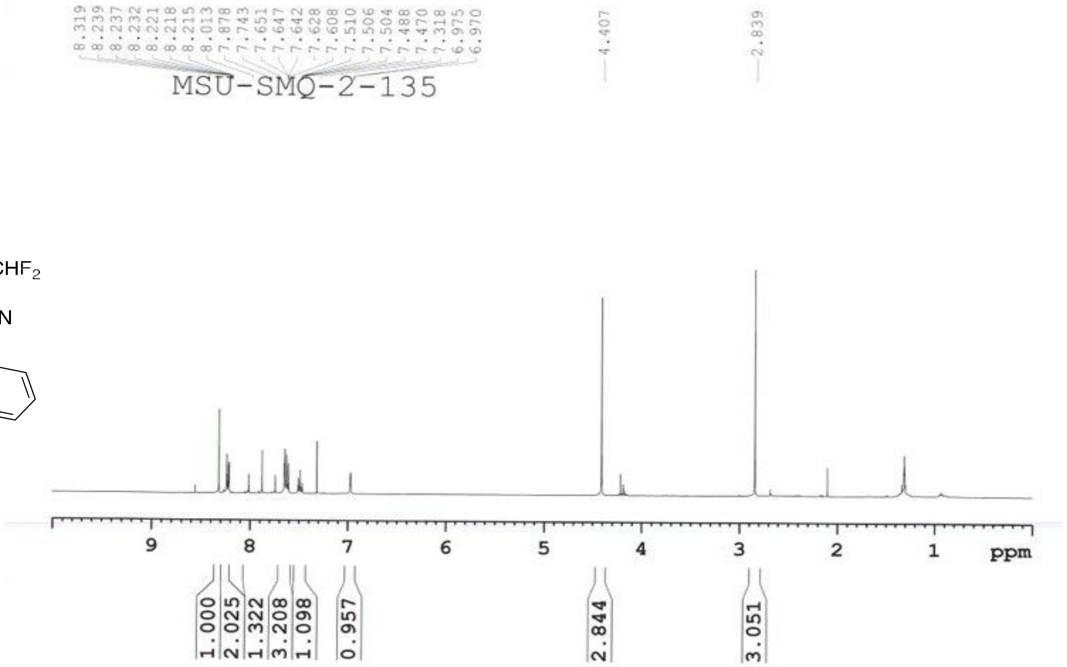
8.319
8.239
8.237
8.232
8.221
8.218
8.215
8.013
7.878
7.743
7.651
7.647
7.642
7.628
7.608
7.510
7.506
7.504
7.488
7.470
7.318
6.975
6.970
4.407
2.839
MSU-SMQ-2-135
9 8 7 6 5 4 3 2 1 ppm
1.000
2.025
1.322
3.208
1.098
0.957
2.844
3.051

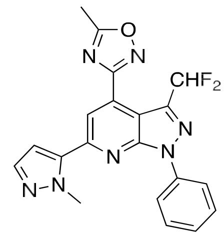
O
N
N
CHF2
N
N
N
N
N

7

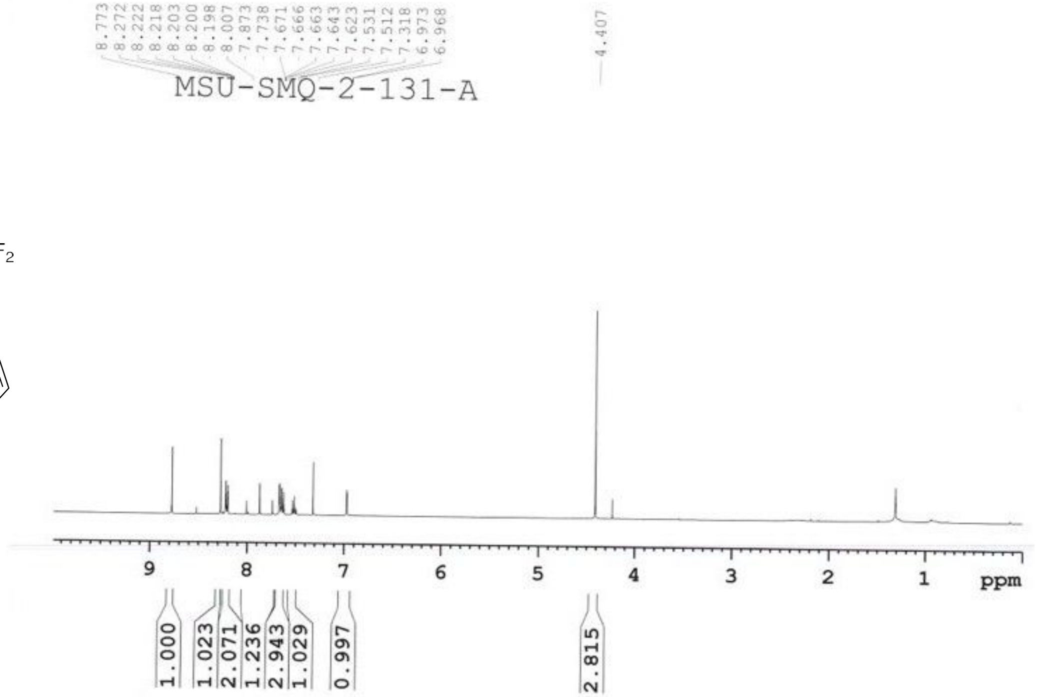
8.773
8.272
8.222
8.218
8.203
8.200
8.198
8.007
7.873
7.738
7.671
7.666
7.663
7.643
7.623
7.531
7.512
7.318
6.973
6.968
4.407
MSU-SMQ-2-131-A
9 8 7 6 5 4 3 2 1 ppm
1.000
1.023
2.071
1.236
2.943
1.029
0.997
2.815

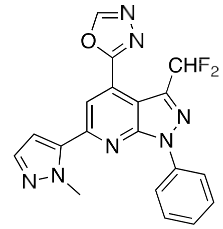
N
N
O
CHF2
N
N
N
N
N

8a

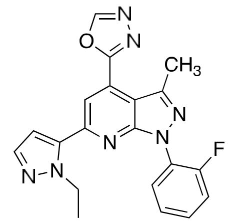

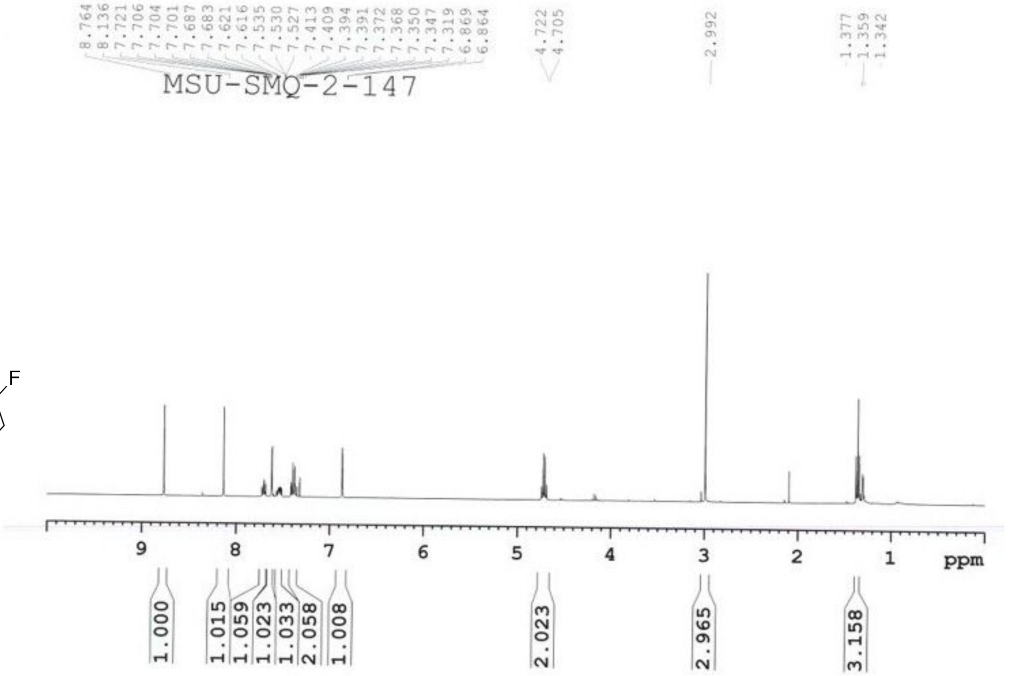

MSU-SMQ-2-147
8.764 8.136 7.721 7.706 7.704 7.701 7.687 7.683 7.621 7.616 7.535 7.530 7.527 7.413 7.409 7.394 7.391 7.372 7.368 7.350 7.347 7.319 6.869 6.864
4.722 4.705
2.992
1.377 1.359 1.342
9 8 7 6 5 4 3 2 1 ppm
1.000 1.015 1.059 1.023 1.033 2.058 1.008 2.023 2.965 3.158

8b

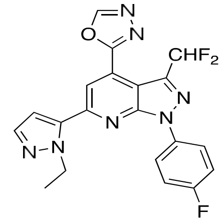

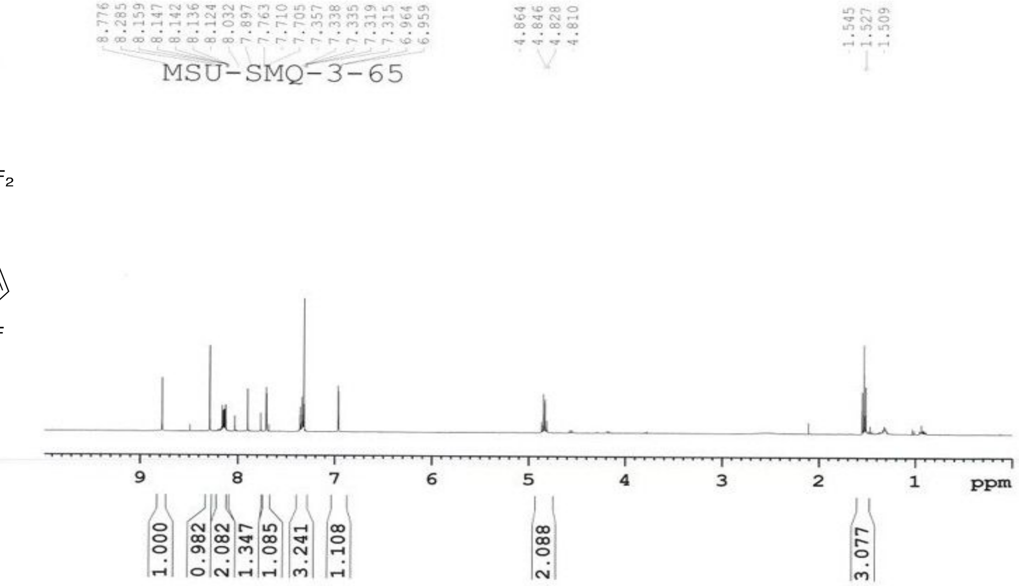

MSU-SMQ-3-65
8.776 8.285 8.159 8.147 8.142 8.136 8.124 8.032 7.897 7.763 7.710 7.705 7.357 7.338 7.335 7.319 7.315 6.964 6.959
4.864 4.846 4.828 4.810
1.545 1.527 1.509
9 8 7 6 5 4 3 2 1 ppm
1.000 0.982 2.082 1.347 1.085 3.241 1.108 2.088 3.077

8c

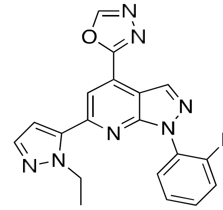
O
N
N
N
N
N
N
N
F

8d

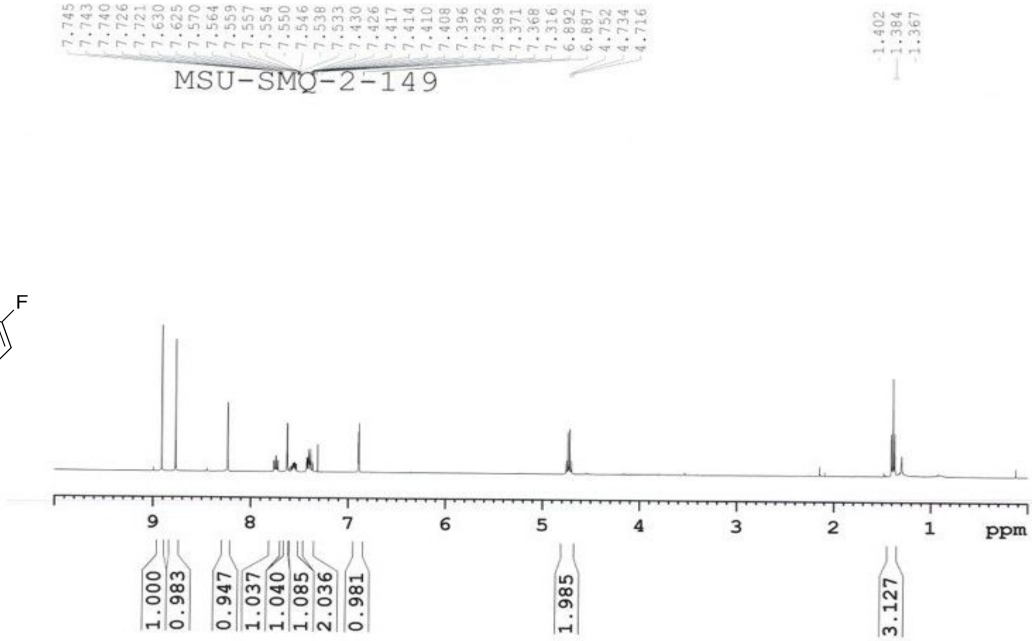
7.745
7.743
7.740
7.726
7.721
7.630
7.625
7.570
7.564
7.559
7.557
7.554
7.550
7.546
7.538
7.533
7.430
7.426
7.417
7.414
7.410
7.408
7.396
7.392
7.389
7.371
7.368
7.316
6.892
6.887
4.752
4.734
4.716
1.402
1.384
1.367
MSU-SMQ-2-149
9
8
7
6
5
4
3
2
1
ppm
1.000
0.983
0.947
1.037
1.040
1.085
2.036
0.981
1.985
3.127

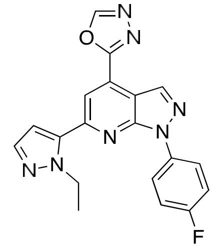
O
N
N
N
N
N
N
N
F

8e

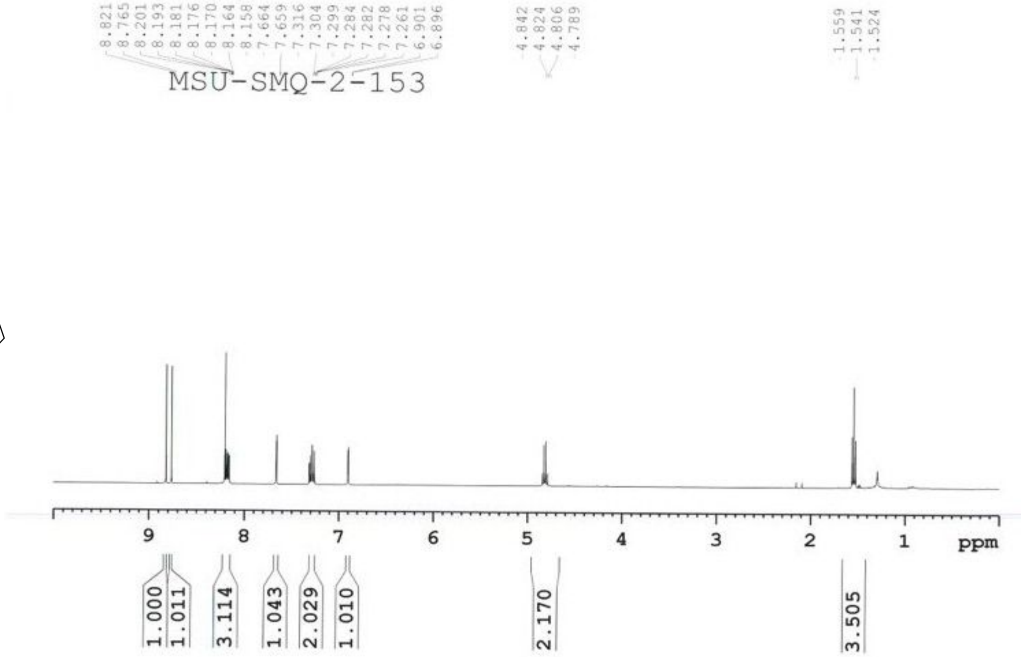
8.821
8.765
8.201
8.193
8.181
8.176
8.170
8.164
8.158
7.664
7.659
7.316
7.304
7.299
7.284
7.282
7.278
7.261
6.901
6.896
4.842
4.824
4.806
4.789
1.559
1.541
1.524
MSU-SMQ-2-153
9
8
7
6
5
4
3
2
1
ppm
1.000
1.011
3.114
1.043
2.029
1.010
2.170
3.505

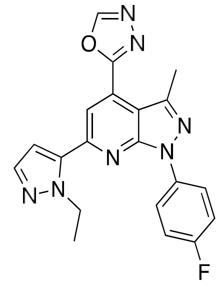

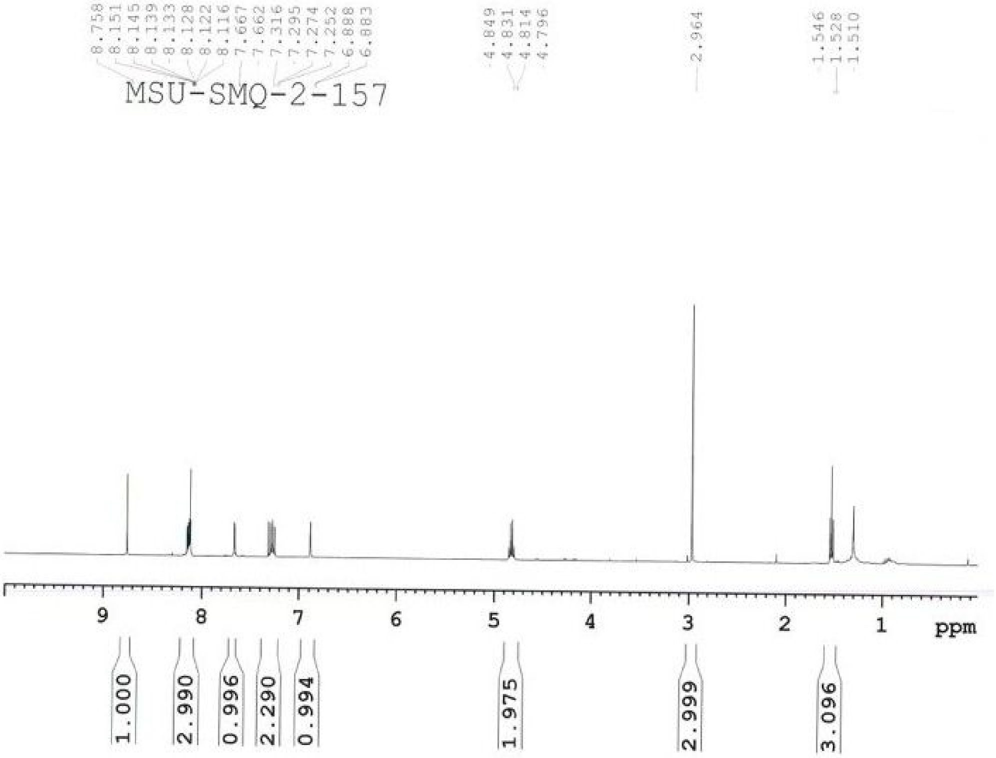
8.758
8.151
8.145
8.139
8.133
8.128
8.122
8.116
7.667
7.662
7.316
7.295
7.274
7.252
6.888
6.883
4.849
4.831
4.814
4.796
2.964
1.546
1.528
1.510
MSU-SMQ-2-157
9
8
7
6
5
4
3
2
1
ppm
1.000
2.990
0.996
2.290
0.994
1.975
2.999
3.096

8f

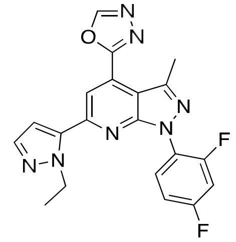

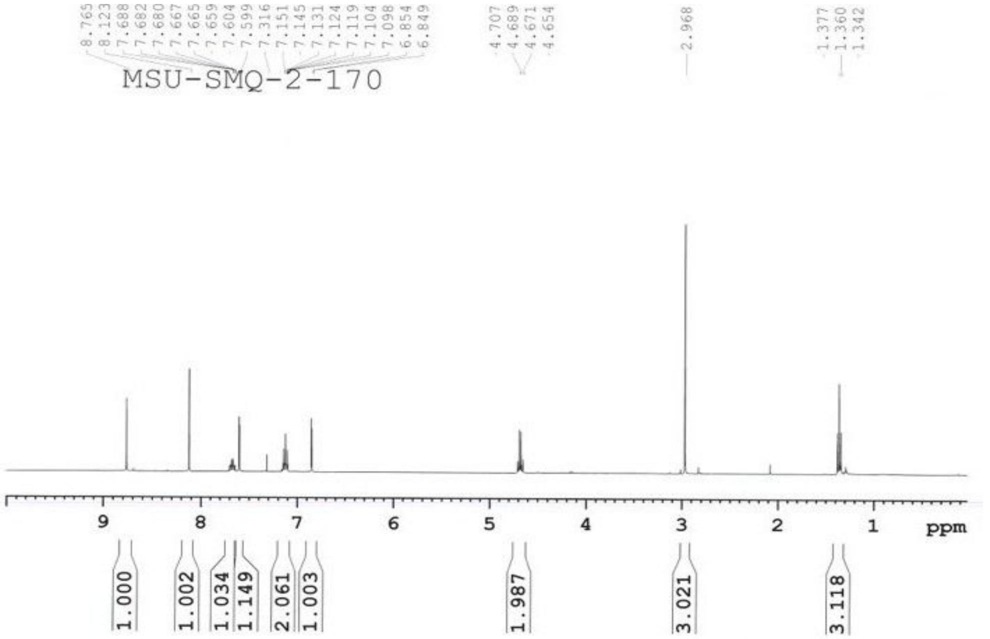
8.765
8.123
7.688
7.682
7.680
7.667
7.665
7.659
7.604
7.599
7.316
7.151
7.145
7.131
7.124
7.119
7.104
7.098
6.854
6.849
4.707
4.689
4.671
4.654
2.968
1.377
1.360
1.342
MSU-SMQ-2-170
9
8
7
6
5
4
3
2
1
ppm
1.000
1.002
1.034
1.149
2.061
1.003
1.987
3.021
3.118

8g

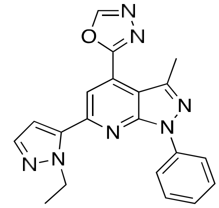

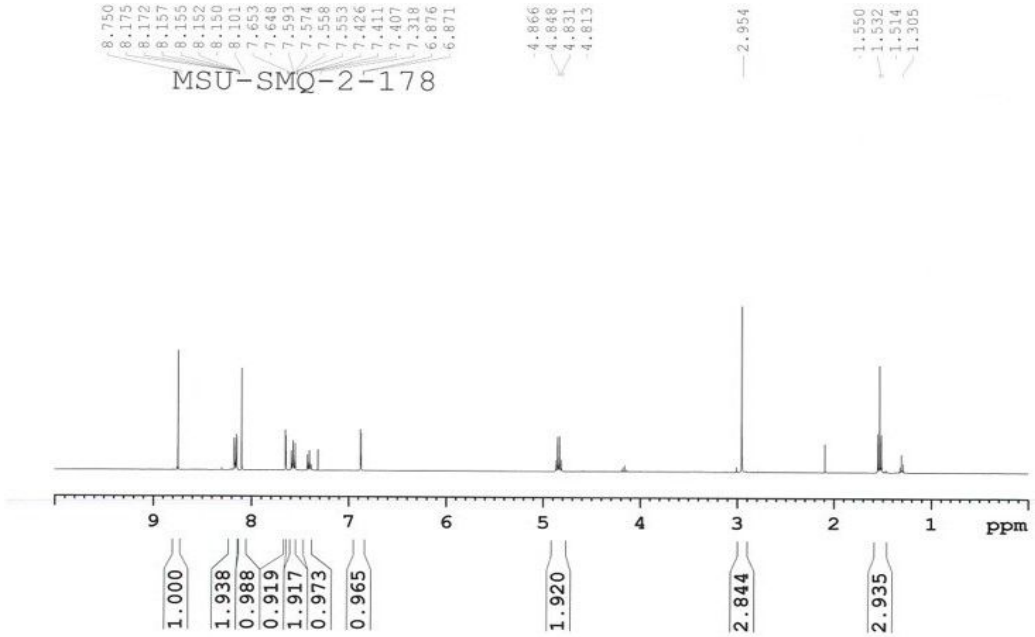
8.750
8.175
8.172
8.157
8.155
8.152
8.150
8.101
7.653
7.648
7.593
7.574
7.558
7.553
7.426
7.411
7.407
7.318
6.876
6.871
4.866
4.848
4.831
4.813
2.954
1.550
1.532
1.514
1.305
MSU-SMQ-2-178
9 8 7 6 5 4 3 2 1 ppm
1.000
1.938
0.988
0.919
1.917
0.973
0.965
1.920
2.844
2.935

8h

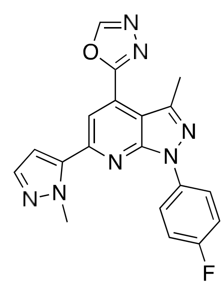
F

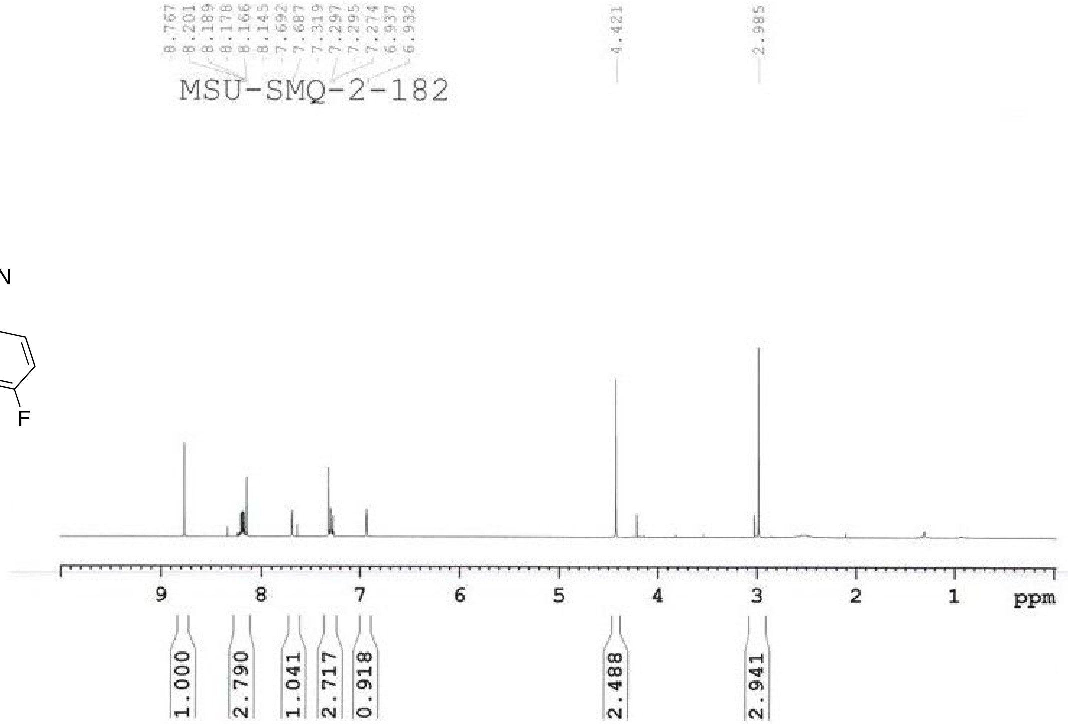
8.767
8.201
8.189
8.178
8.166
8.145
7.692
7.687
7.319
7.297
7.295
7.274
6.937
6.932
4.421
2.985
MSU-SMQ-2-182
9 8 7 6 5 4 3 2 1 ppm
1.000
2.790
1.041
2.717
0.918
2.488
2.941

8i

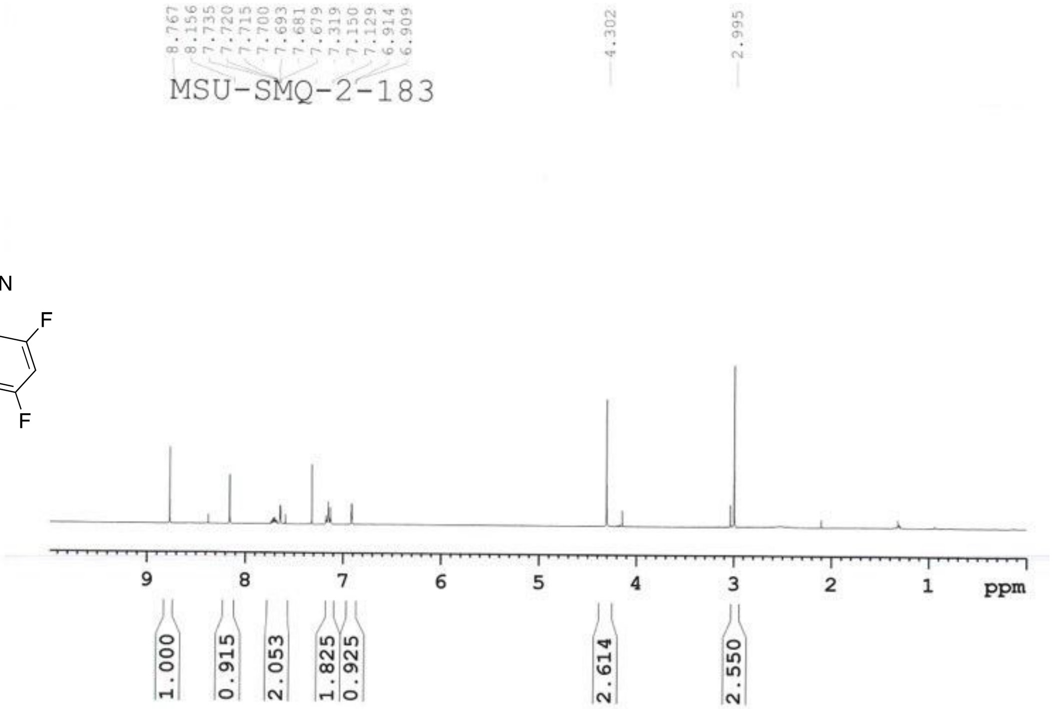
8.767
8.156
7.735
7.720
7.715
7.700
7.693
7.681
7.679
7.319
7.150
7.129
6.914
6.909
4.302
2.995
MSU-SMQ-2-183
9
8
7
6
5
4
3
2
1
ppm
1.000
0.915
2.053
1.825
0.925
2.614
2.550

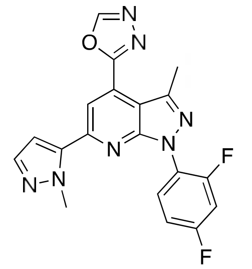

8j

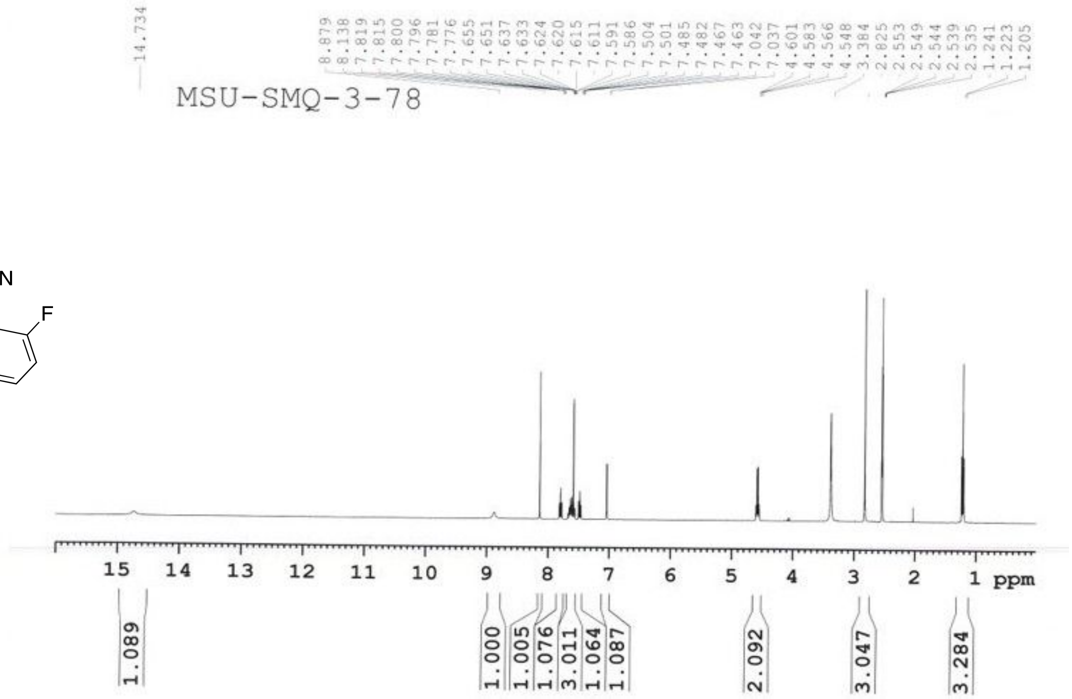
14.734
8.879
8.138
7.819
7.815
7.800
7.796
7.781
7.776
7.655
7.651
7.637
7.633
7.624
7.620
7.615
7.611
7.591
7.586
7.504
7.501
7.485
7.482
7.467
7.463
7.042
7.037
4.601
4.583
4.566
4.548
3.384
2.825
2.553
2.549
2.544
2.539
2.535
1.241
1.223
1.205
MSU-SMQ-3-78
15
14
13
12
11
10
9
8
7
6
5
4
3
2
1
ppm
1.089
1.000
1.005
1.076
3.011
1.064
1.087
2.092
3.047
3.284

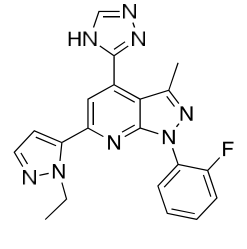

10a

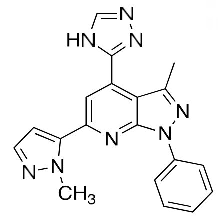
HN
N
N
N
N
N
N
N
CH3

10b

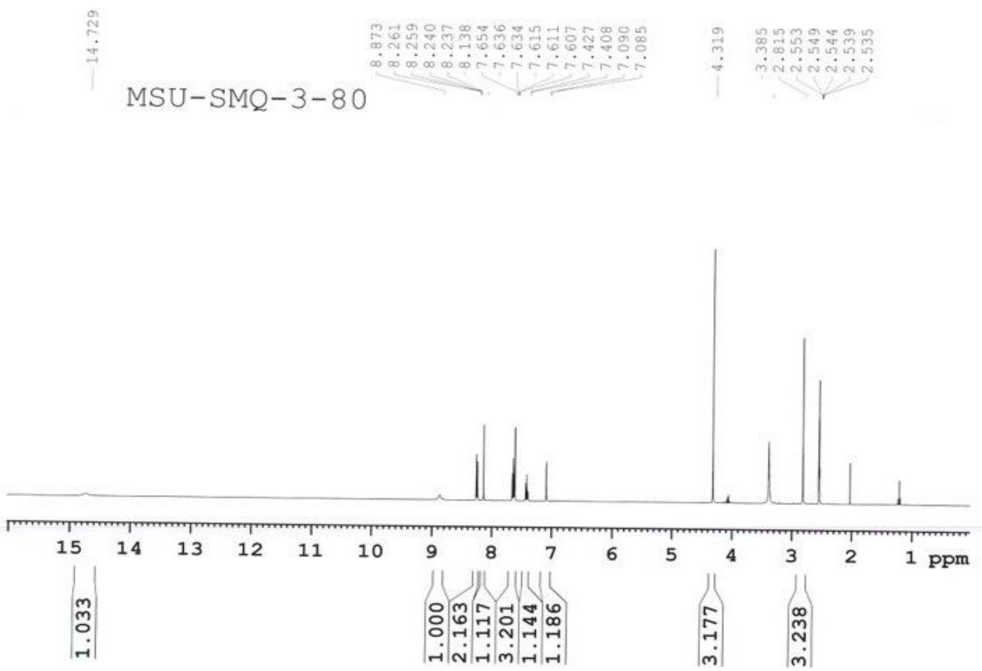
14.729
8.873
8.261
8.259
8.240
8.237
8.138
7.654
7.636
7.634
7.615
7.611
7.607
7.427
7.408
7.090
7.085
4.319
3.385
2.815
2.553
2.549
2.544
2.539
2.535
MSU-SMQ-3-80
15 14 13 12 11 10 9 8 7 6 5 4 3 2 1 ppm
1.033
1.000
2.163
1.117
3.201
1.144
1.186
3.177
3.238

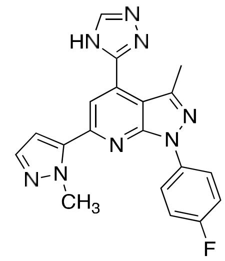
HN
N
N
N
N
N
N
N
CH3
F

10c

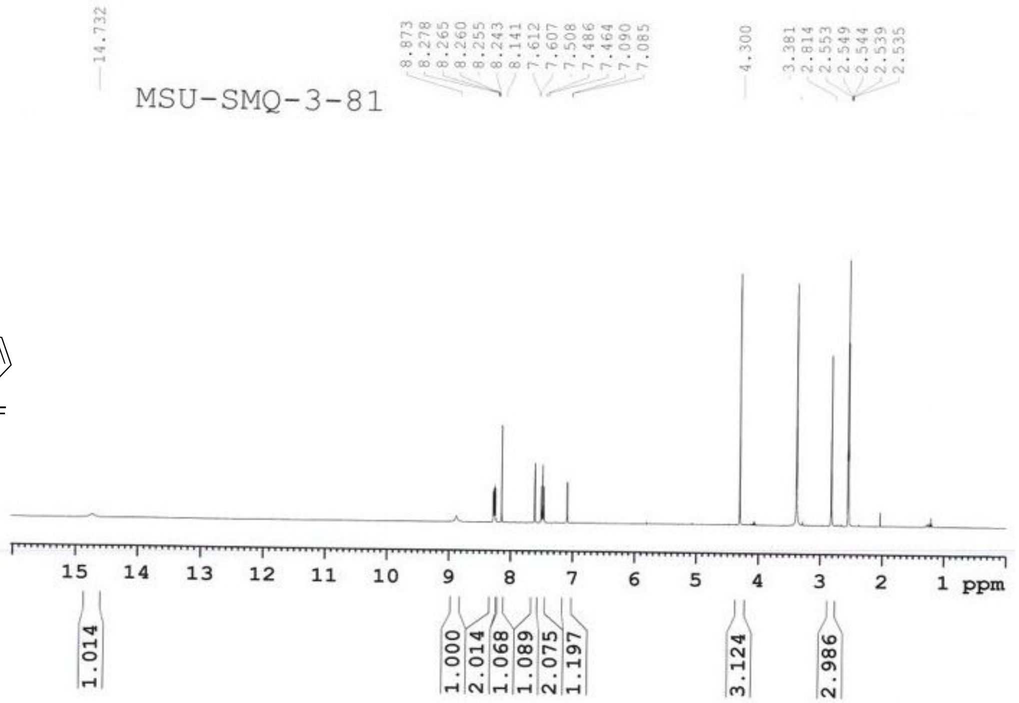
14.732
8.873
8.278
8.265
8.260
8.255
8.243
8.141
7.612
7.607
7.508
7.486
7.464
7.090
7.085
4.300
3.381
2.814
2.553
2.549
2.544
2.539
2.535
MSU-SMQ-3-81
15 14 13 12 11 10 9 8 7 6 5 4 3 2 1 ppm
1.014
1.000
2.014
1.068
1.089
2.075
1.197
3.124
2.986

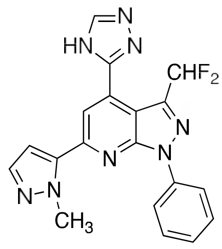

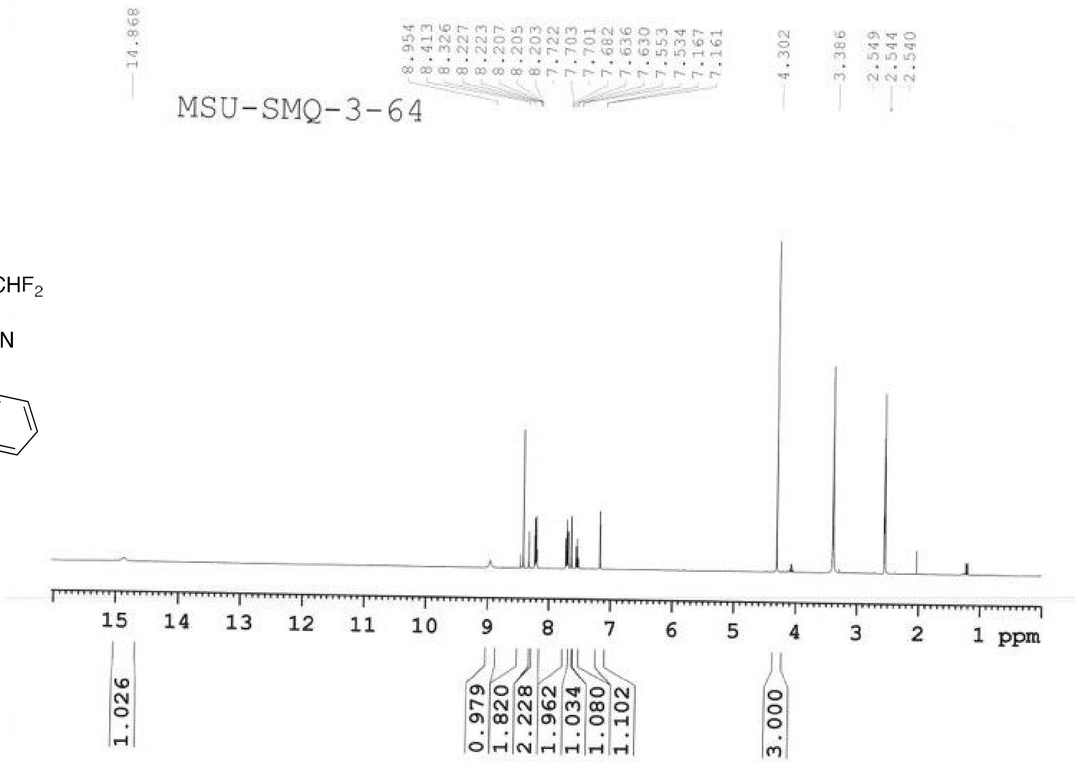
14.868
8.954
8.413
8.326
8.227
8.223
8.207
8.205
8.203
7.722
7.703
7.701
7.682
7.636
7.630
7.553
7.534
7.167
7.161
4.302
3.386
2.549
2.544
2.540
MSU-SMQ-3-64
15 14 13 12 11 10 9 8 7 6 5 4 3 2 1 ppm
1.026
0.979
1.820
2.228
1.962
1.034
1.080
1.102
3.000

10d

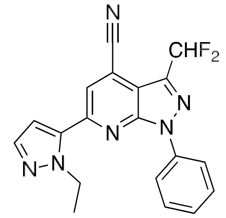

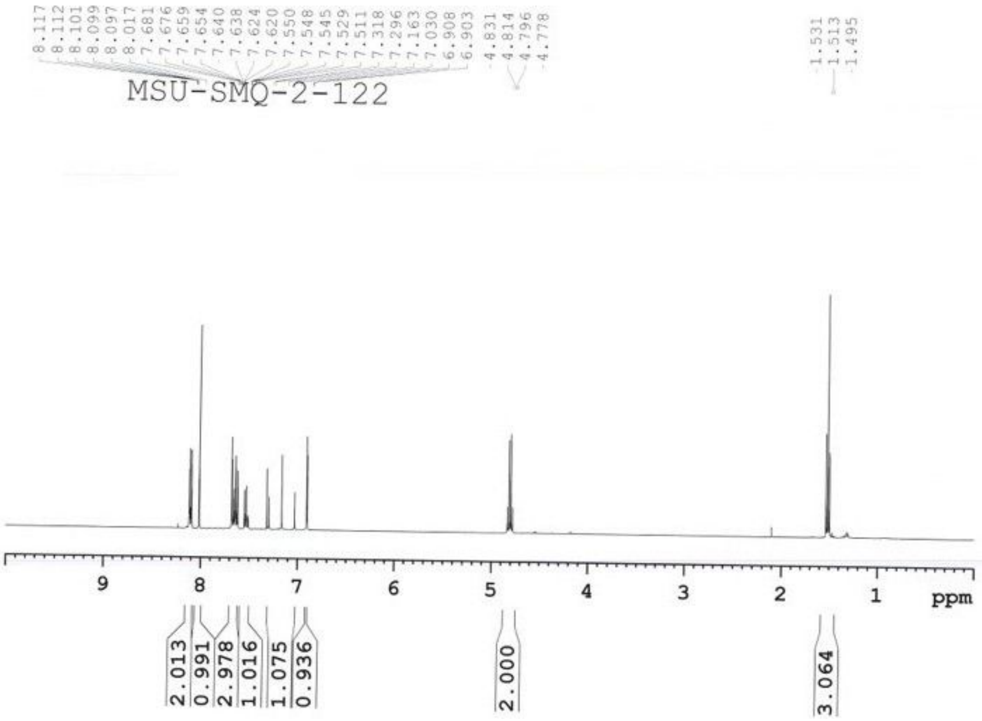
8.117
8.112
8.101
8.099
8.097
8.017
7.681
7.676
7.659
7.654
7.640
7.638
7.624
7.620
7.550
7.548
7.545
7.529
7.511
7.318
7.296
7.163
7.030
6.908
6.903
4.831
4.814
4.796
4.778
1.531
1.513
1.495
MSU-SMQ-2-122
9 8 7 6 5 4 3 2 1 ppm
2.013
0.991
2.978
1.016
1.075
0.936
2.000
3.064

11

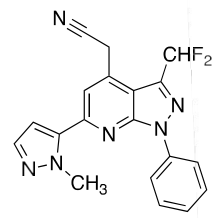
N
CHF2
N
N
N
N
N
CH3

12

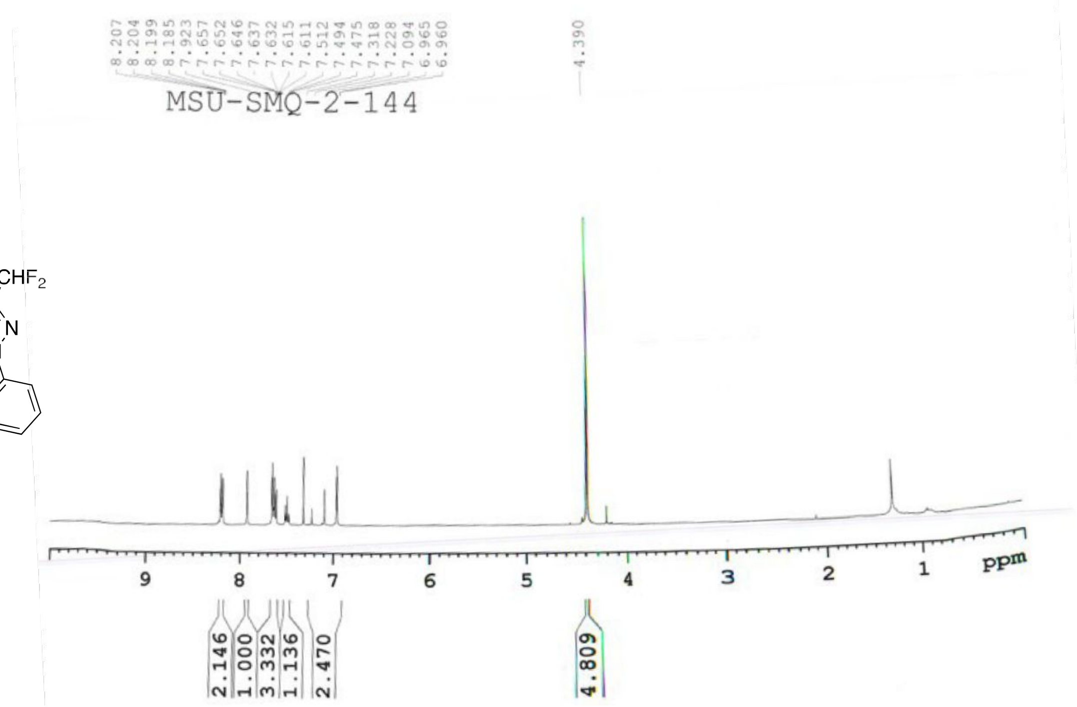
8.207
8.204
8.199
8.185
7.923
7.657
7.652
7.646
7.637
7.632
7.615
7.611
7.512
7.494
7.475
7.318
7.228
7.094
6.965
6.960
4.390
MSU-SMQ-2-144
9
8
7
6
5
4
3
2
1
ppm
2.146
1.000
3.332
1.136
2.470
4.809

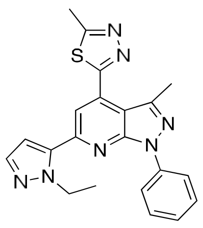

4a

miniLC

Method: 6 MINUTE ISOCRATIC 80% acn
Batch: A
Sample: MSU-SMQ-1-089
User: Guest
S/N: 0118000000
Date: 11/08/2022 11:48 PM

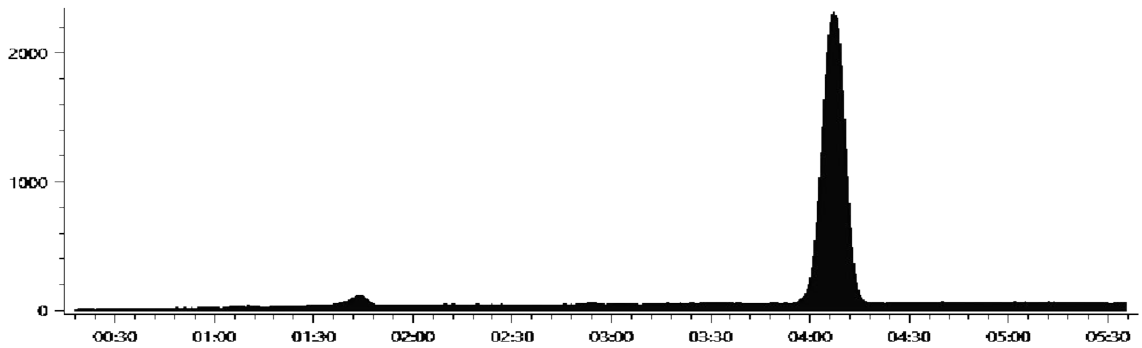

#	Compound	Retention Time	Area	% Area	Height
1		01:43.5	477.9	2.7	73.71
2		04:07.5	17411.0	97.3	2259.46

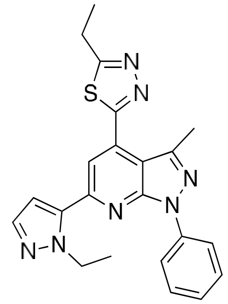

4b

miniLC

Method: 6 MINUTE ISOCRATIC 80% acn
Batch: BN
Sample: MSU-SMQ-1-97
User: Guest
S/N: 0118000000
Date: 11/11/2022 6:02 AM

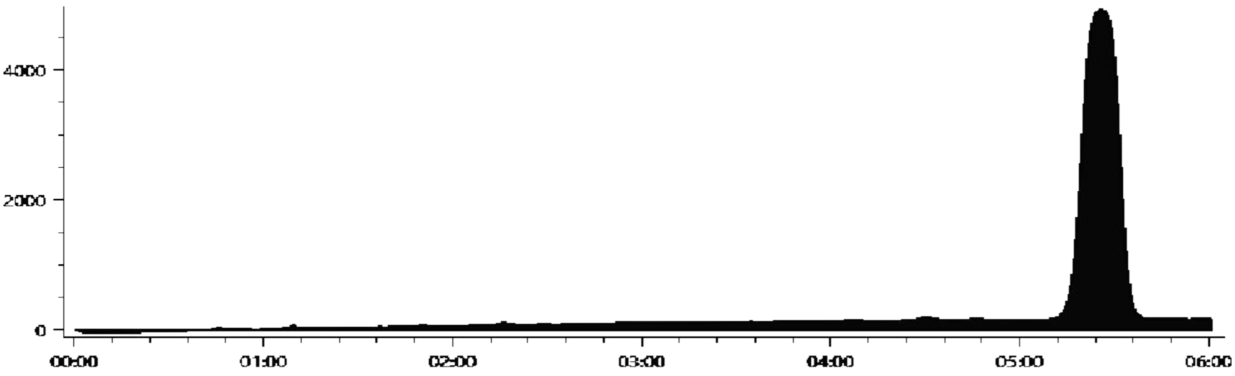

#	Compound	Retention Time	Area	% Area	Height
1		05:26.0	67310.1	100.0	4853.73

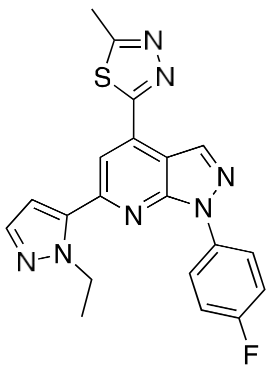

4c

miniLC

Method: 6 MINUTE ISOCRATIC 80% acn
Batch: BN
Sample: MSU-SMQ-1-107
User: Guest
S/N: 0118000000
Date: 11/11/2022 6:10 AM

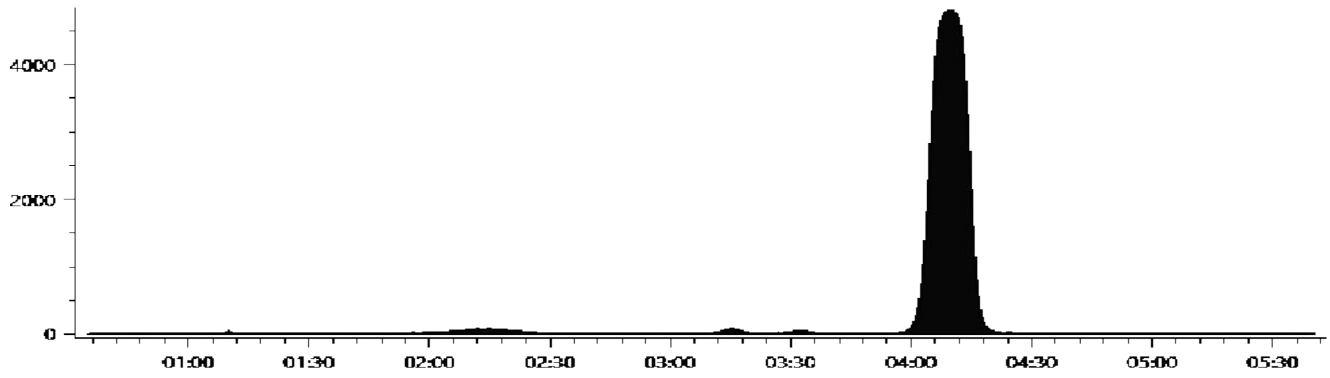

#	Compound	Retention Time	Area	% Area	Height
1		02:14.5	1092.3	2.2	66.92
2		03:15.0	336.5	0.7	67.72
3		04:10.0	49190.2	97.2	4788.79

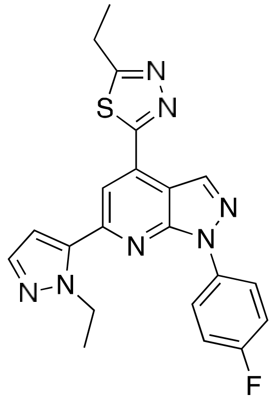

miniLC

Method: 6 MINUTE ISOCRATIC 80% acn
Batch: h
Sample: MSU-SMQ-1-108
User: Administrator
S/N: 0118000000
Date: 02/21/2024 5:09 PM

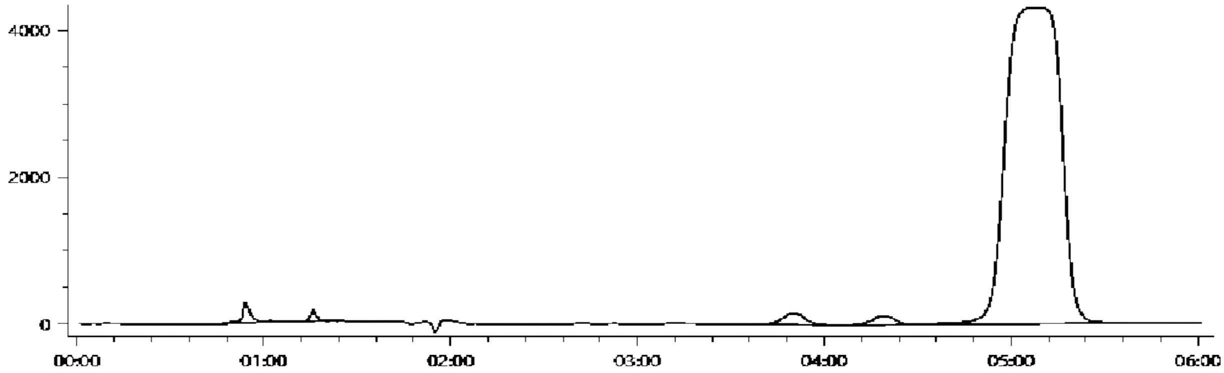

#	Compound	Retention Time	Area	% Area	Height
1		00:54.5	854.2	1.0	368.49
2		01:16.0	241.3	0.3	318.02
3		03:50.5	1319.3	1.5	234.59
4		04:19.0	1038.9	1.2	195.37
5		05:08.0	85756.1	96.1	4387.50

4d

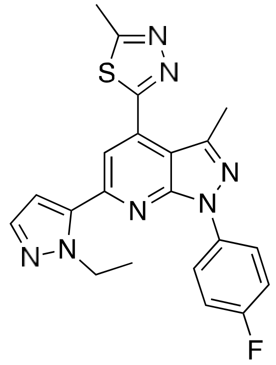

miniLC

Method: 6 MINUTE ISOCRATIC 80% acn
Batch: BN
Sample: MSU-SMQ-1-175
User: Guest
S/N: 0118000000
Date: 11/11/2022 6:17 AM

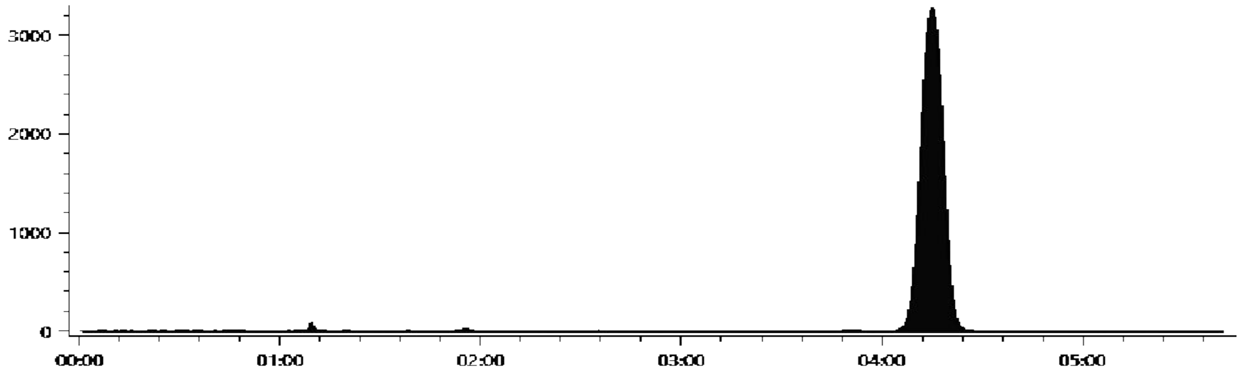

#	Compound	Retention Time	Area	% Area	Height
1		01:09.5	188.5	0.7	96.09
2		04:15.0	24998.8	99.3	3279.85

4e

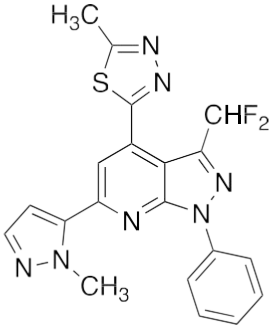

miniLC

Method: 6 MINUTE ISOCRATIC 80% acn
Batch: m
Sample: MSU-SMQ-2-60
User: Administrator
S/N: 0118000000
Date: 02/21/2024 6:46 PM

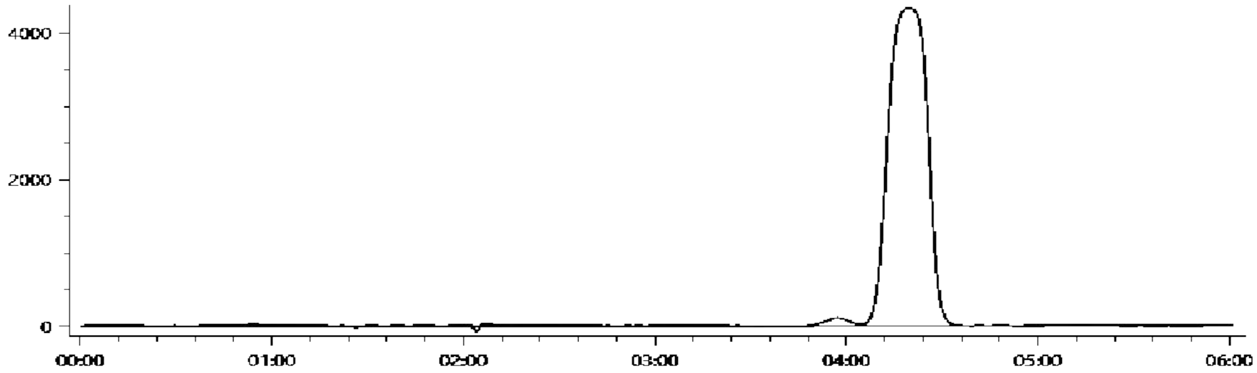

#	Compound	Retention Time	Area	% Area	Height
1		04:19.5	63029.5	100.0	4402.32

4f

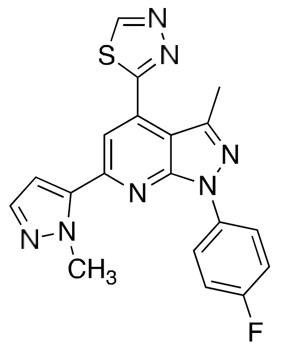

miniLC

Method: 6 MINUTE ISOCRATIC 80% acn
Batch: I
Sample: MSU-SMQ-3-20
User: Admin
S/N: 0118000000
Date: 08/07/2023 4:25 PM

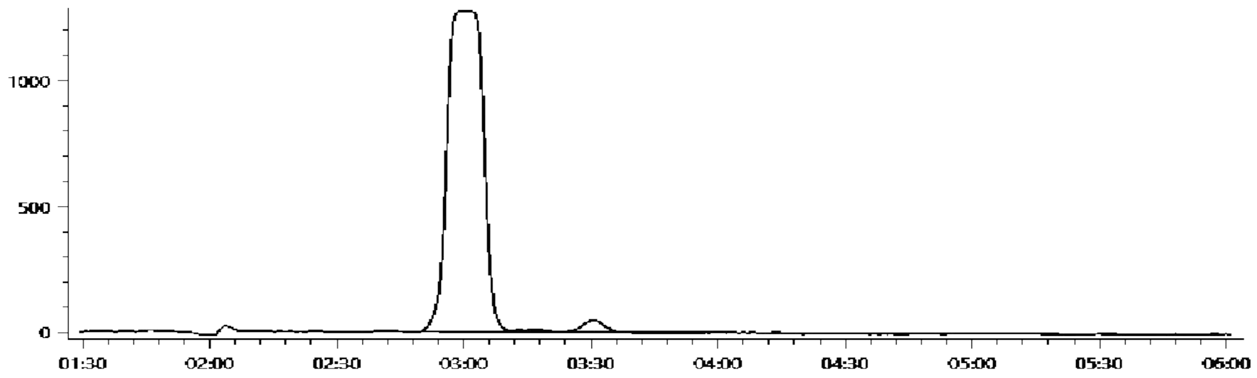

#	Compound	Retention Time	Area	% Area	Height
1		03:00.5	12491.8	100.0	1278.73

4g

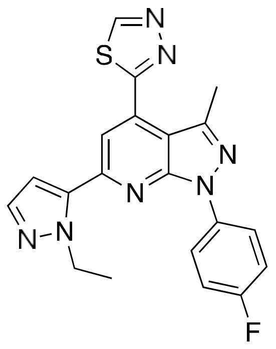

miniLC

Method: 6 MINUTE ISOCRATIC 80% acn
Batch: O
Sample: MSU-SMQ-3-30
User: Admin
S/N: 0118000000
Date: 08/15/2023 10:49 AM

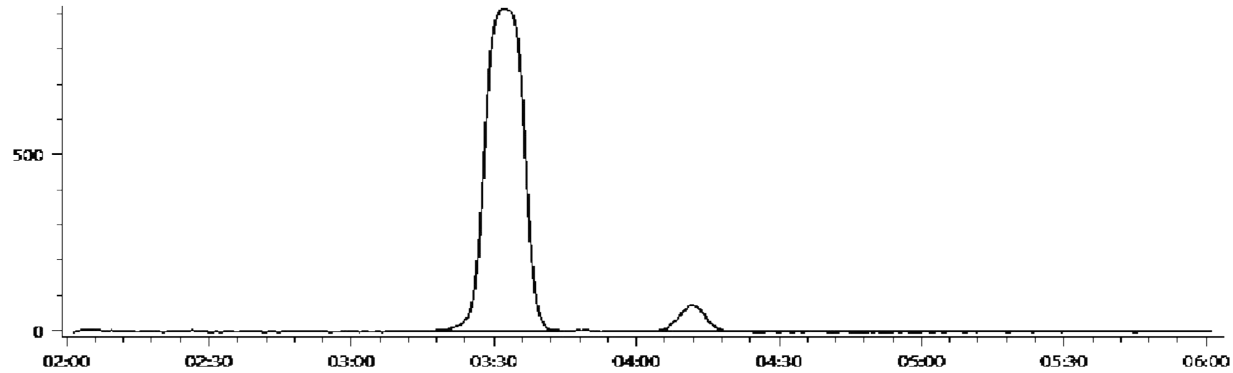

#	Compound	Retention Time	Area	% Area	Height
1		03:32.5	8526.3	94.7	915.23
2		04:12.0	316.3	5.3	73.58

4h

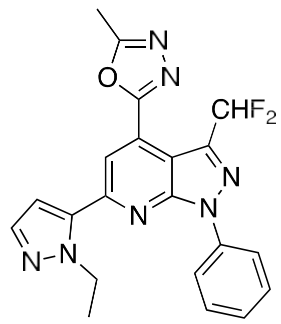

miniLC

Method: 6 MINUTE ISOCRATIC 80% acn
Batch: m
Sample: MSU-SMQ-2-106
User: Administrator
S/N: 0118000000
Date: 02/21/2024 7:31 PM

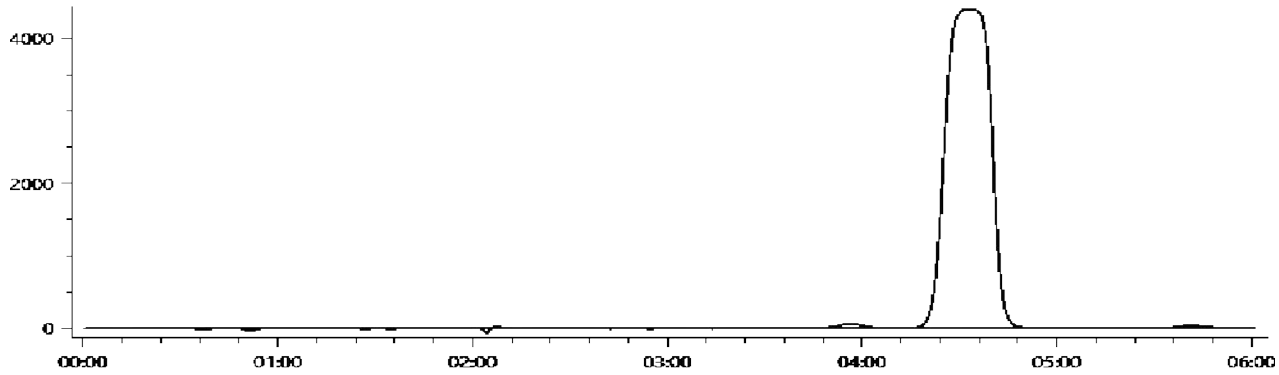

#	Compound	Retention Time	Area	% Area	Height
1		04:33.0	71103.5	100.0	4452.89

5a

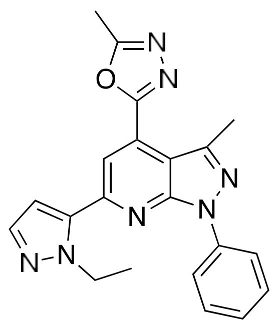

miniLC

Method: 6 MINUTE ISOCRATIC 80% acn
Batch: n
Sample: MSU-SMQ-1-120
User: Administrator
S/N: 0118000000
Date: 02/21/2024 7:08 PM

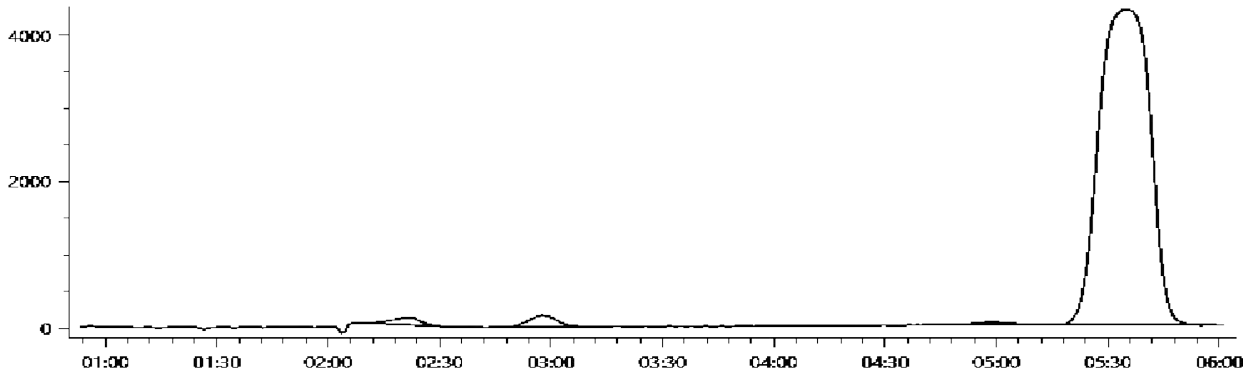

#	Compound	Retention Time	Area	% Area	Height
1		02:21.0	892.3	1.3	225.08
2		02:57.5	1394.5	2.0	236.51
3		04:58.0	397.5	0.6	89.19
4		05:35.0	68466.3	96.2	4327.30

5b

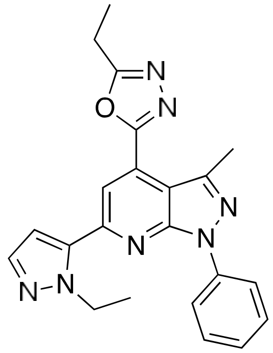

miniLC

Method: 6 MINUTE ISOCRATIC 80% acn
Batch: nb
Sample: MSU-SMQ-1-122
User: Administrator
S/N: 0118000000
Date: 07/09/2024 12:23 PM

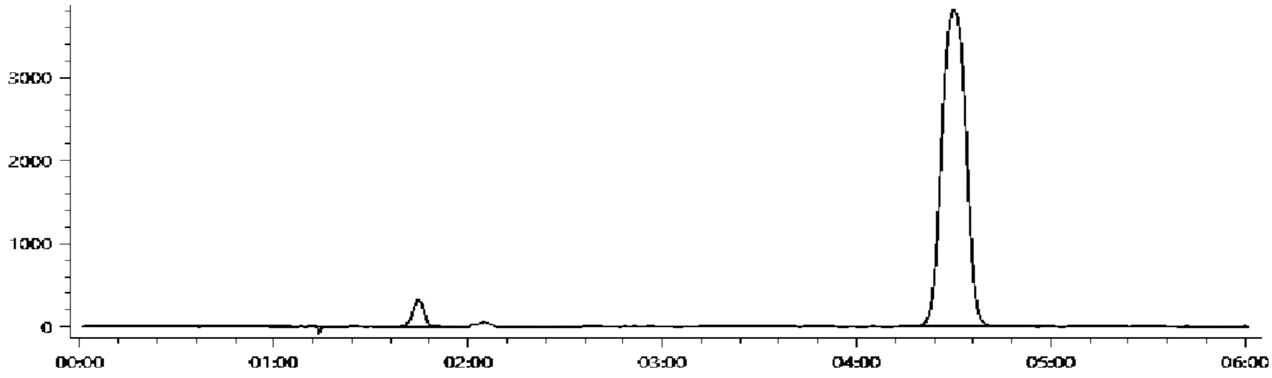

#	Compound	Retention Time	Area	% Area	Height
1		01:45.0	1128.4	3.3	430.79
2		04:30.0	32789.2	96.7	3881.25

5c

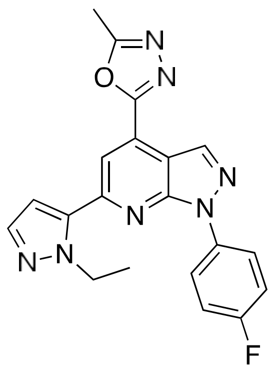

miniLC

Method: 6 MINUTE ISOCRATIC 80% acn
Batch: o
Sample: MSU-SMQ-1-123
User: Administrator
S/N: 0118000000
Date: 02/22/2024 5:05 PM

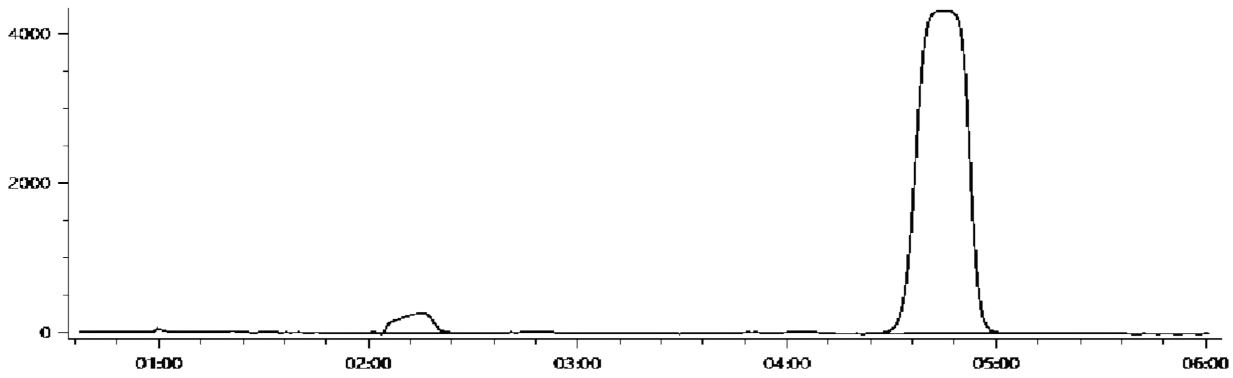

#	Compound	Retention Time	Area	% Area	Height
1		02:13.5	2996.0	4.1	280.92
2		04:44.5	70386.0	95.9	4335.89

5d

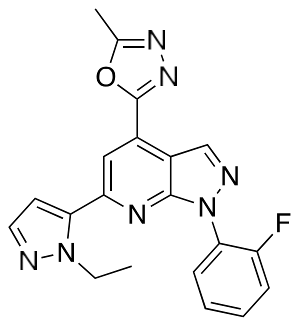

miniLC

Method: 6 MINUTE ISOCRATIC 80% acn
Batch: mm
Sample: MSU-SMQ-1-163
User: Administrator
S/N: 0118000000
Date: 02/22/2024 5:42 PM

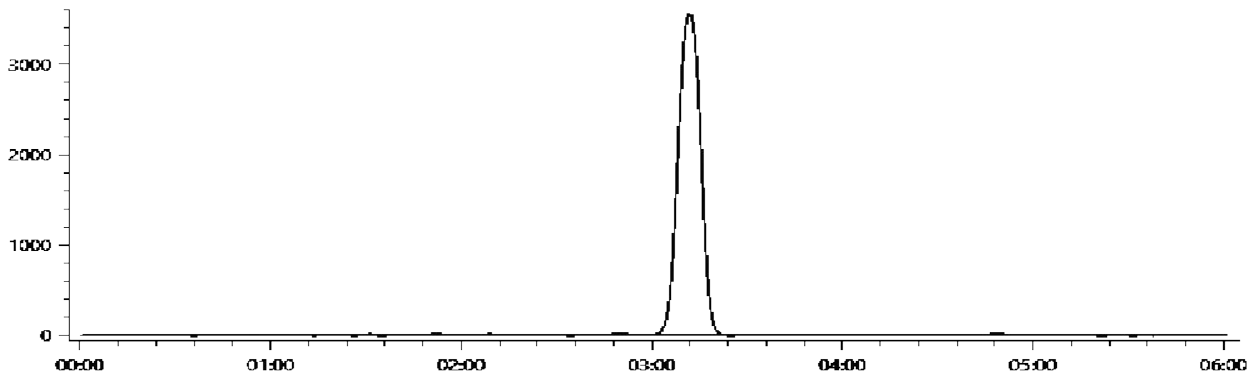

#	Compound	Retention Time	Area	% Area	Height
1		03:12.0	29500.8	100.0	3596.70

5e

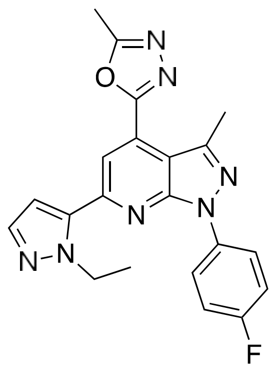

LC-UV Chromatogram

Method: 10 Minute Isocratic
Batch: p
Sample: MSU-SMQ-1-172
User: Administrator
S/N: 0118000000
Date: 02/22/2024 6:51 PM

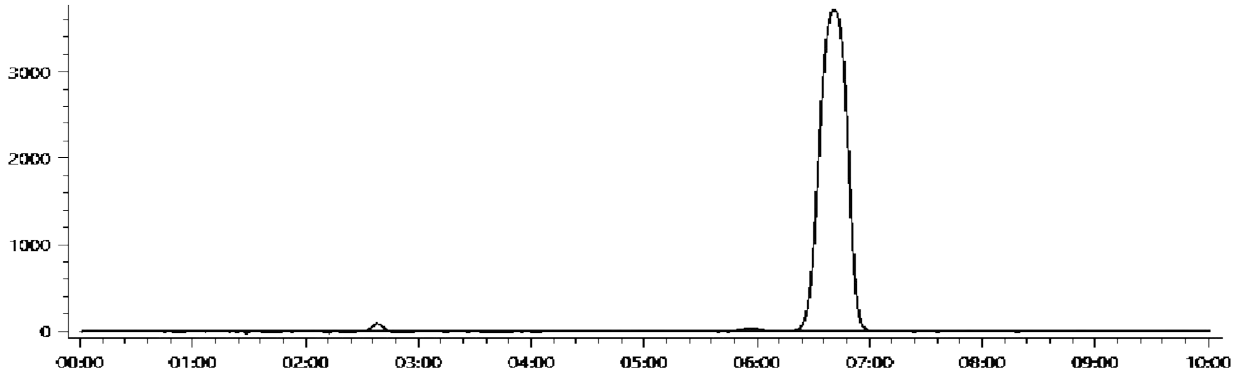

#	Compound	Retention Time	Area	% Area	Height	Conc.
1		06:41.2	64859.7	100.0	3717.51	0.000

5f

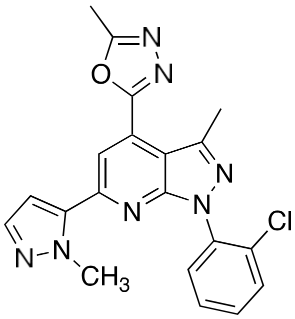

miniLC

Method: 6 MINUTE ISOCRATIC 80% acn
Batch: lo
Sample: MSU-SMQ-1-181
User: Administrator
S/N: 0118000000
Date: 07/13/2024 2:24 PM

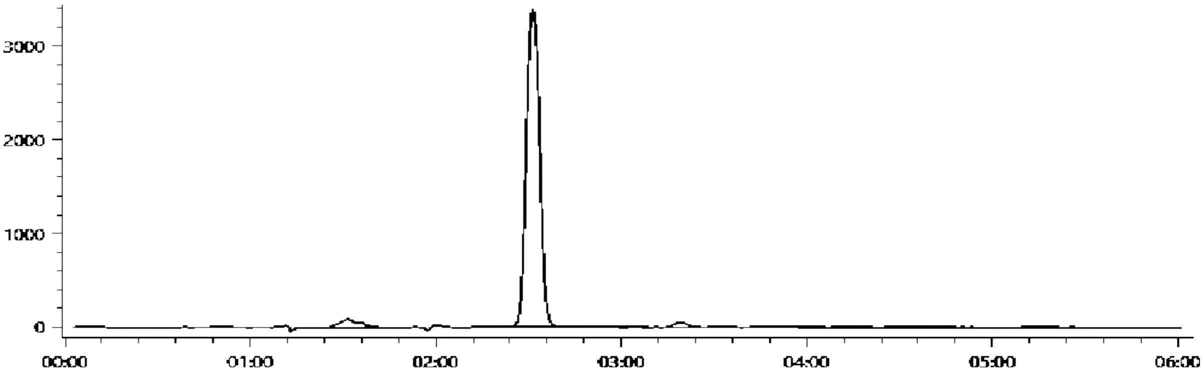

#	Compound	Retention Time	Area	% Area	Height
1		01:32.0	748.7	4.2	148.62
2		02:31.5	17730.9	95.8	3445.52

5g

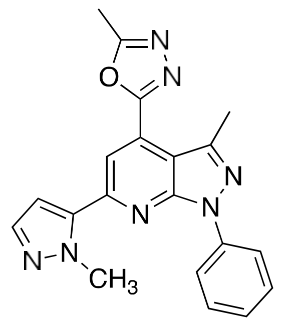

miniLC

Method: 6 MINUTE ISOCRATIC 80% acn
Batch: ui
Sample: MSU-SMQ-2-050
User: Administrator
S/N: 0118000000
Date: 07/09/2024 9:47 AM

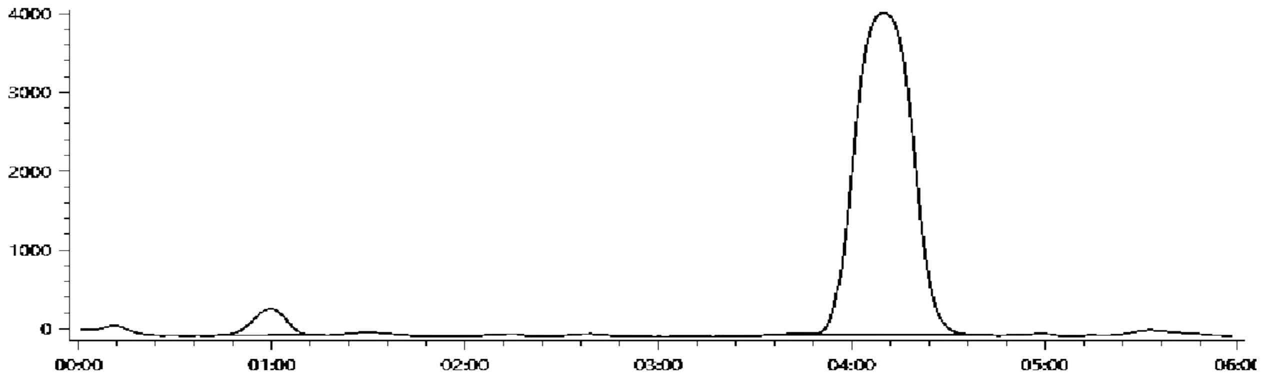

#	Compound	Retention Time	Area	% Area	Height
1		00:59.5	4055.8	4.6	370.19
2		04:10.0	83703.0	95.4	4149.70

5h

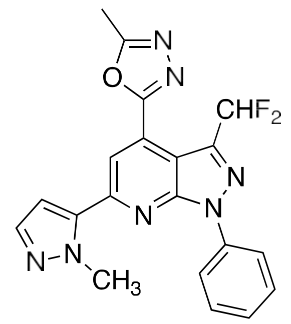

LC-UV Chromatogram

Method: 10 Minute Isocratic
Batch: p
Sample: MSU-SMQ-2-56
User: Administrator
S/N: 0118000000
Date: 02/22/2024 7:01 PM

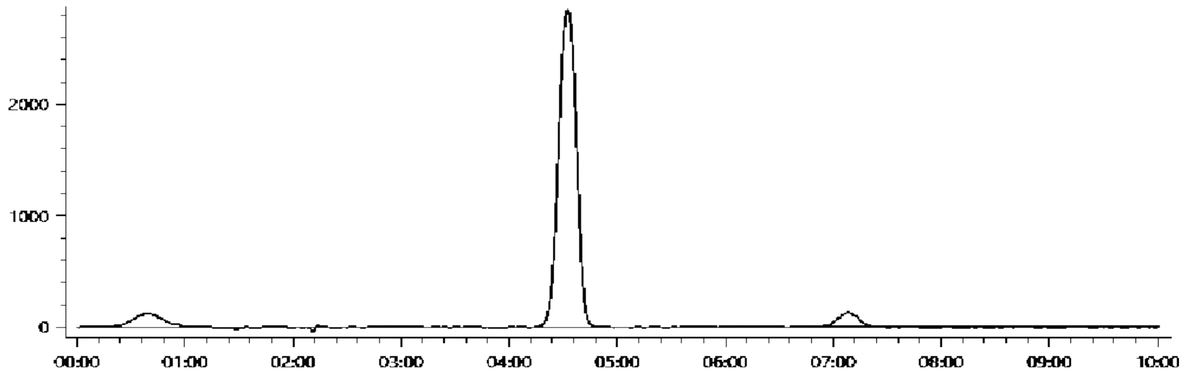

#	Compound	Retention Time	Area	% Area	Height	Conc.
1		04:32.6	37548.8	100.0	2883.81	0.000

5i

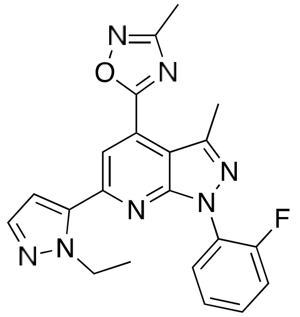

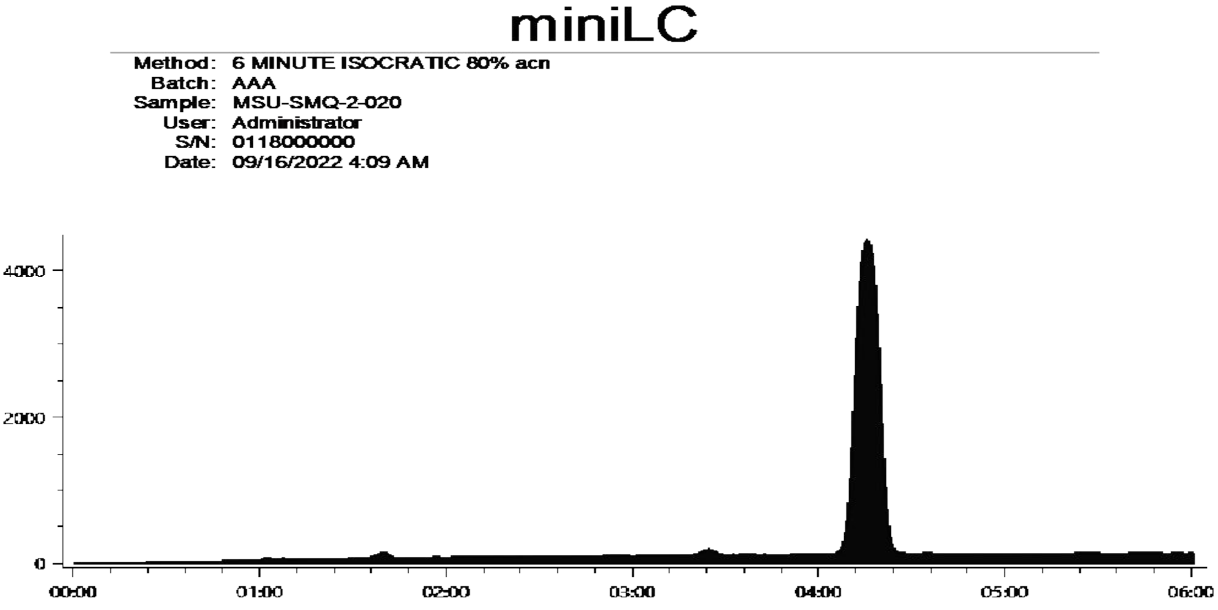

#	Compound	Retention Time	Area	% Area	Height
1		04:16.0	47980.8	100.0	4364.40

6a

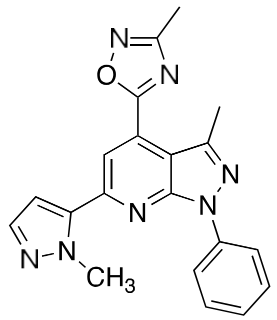

miniLC

Method: 6 MINUTE ISOCRATIC 80% acn
Batch: ui
Sample: MSU-SMQ-2-051
User: Administrator
S/N: 0118000000
Date: 07/09/2024 11:40 AM

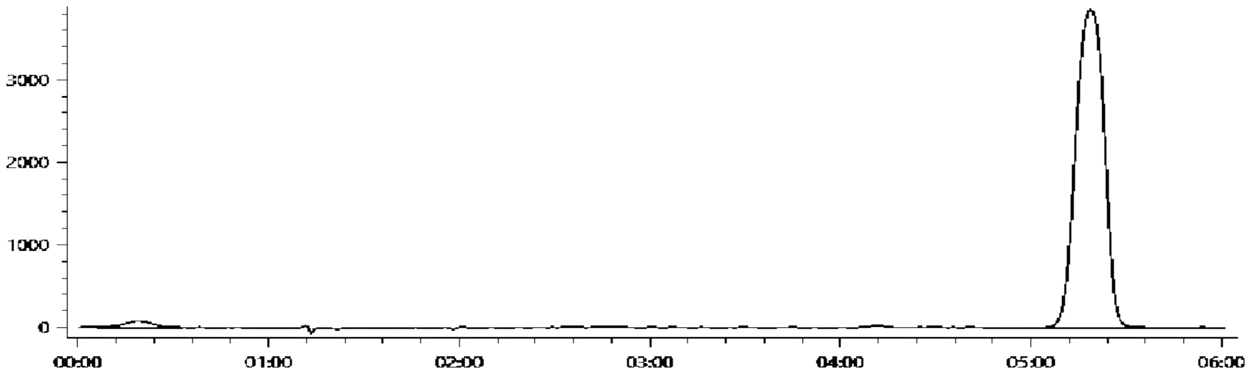

#	Compound	Retention Time	Area	% Area	Height
1		00:19.0	978.3	2.4	141.03
2		05:19.0	39910.9	97.6	3882.57

6b

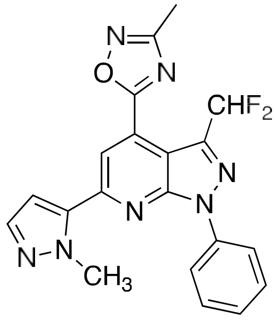

miniLC

Method: 6 MINUTE ISOCRATIC 80% acn
Batch: pp
Sample: MSU-SMQ-2-55
User: Administrator
S/N: 0118000000
Date: 02/23/2024 4:28 PM

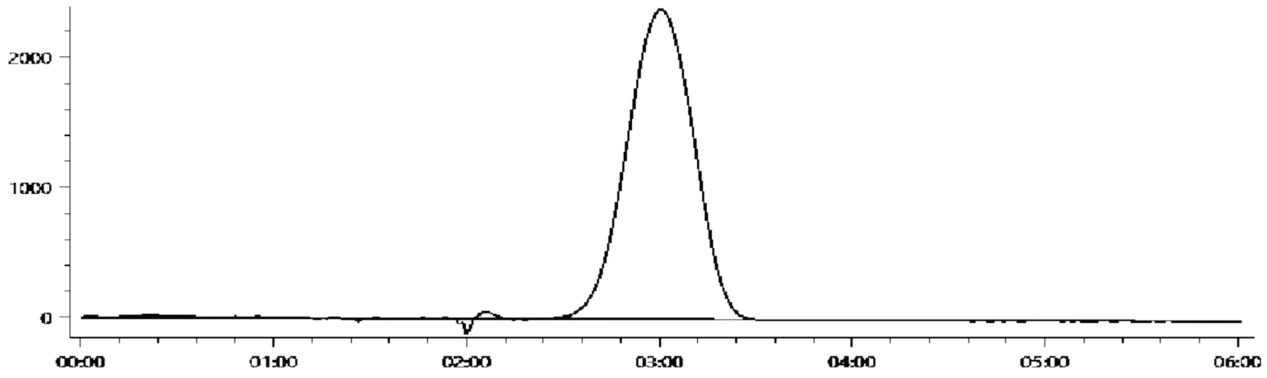

#	Compound	Retention Time	Area	% Area	Height
1		03:00.5	57134.8	100.0	2492.21

6c

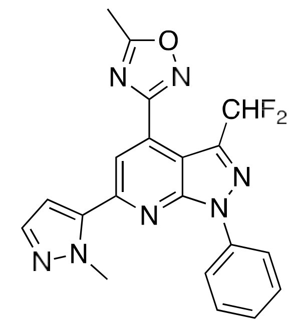

miniLC

Method: 6 MINUTE ISOCRATIC 80% acn
Batch: po
Sample: MSU-SMQ-2-135
User: Administrator
S/N: 0118000000
Date: 07/10/2024 4:12 PM

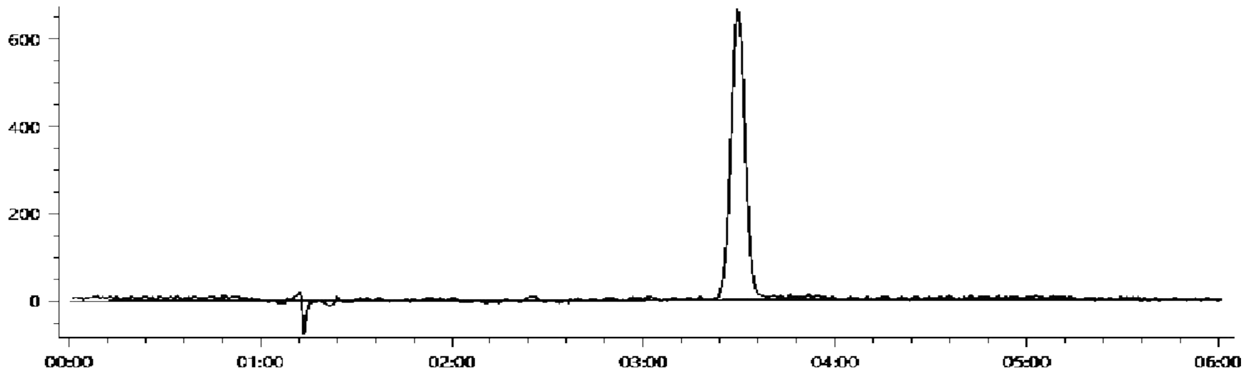

#	Compound	Retention Time	Area	% Area	Height
1		03:29.5	4345.3	100.0	710.93

7

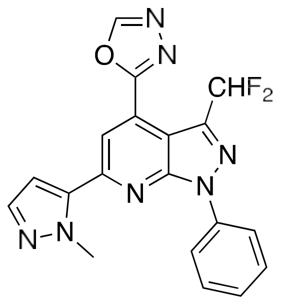

miniLC

Method: 6 MINUTE ISOCRATIC 80% acn
Batch: oii
Sample: MSU-SMQ-2-131
User: Administrator
S/N: 0118000000
Date: 07/10/2024 3:43 PM

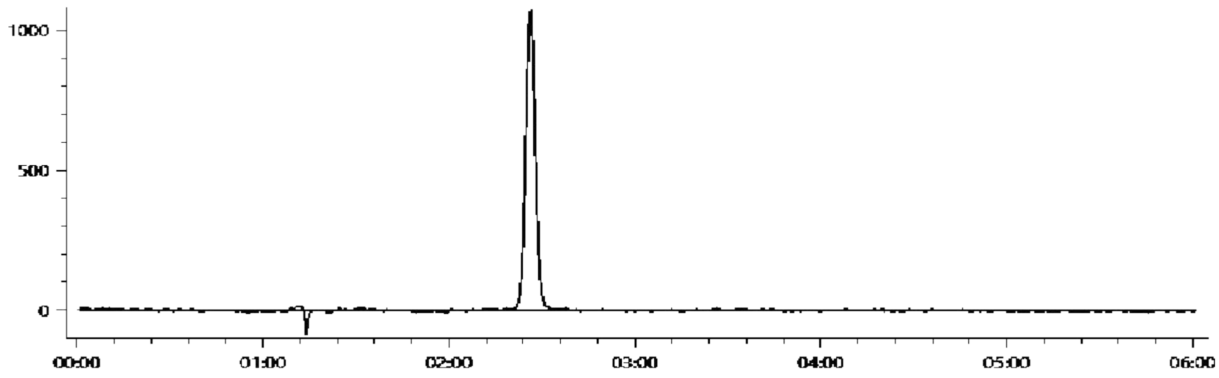

#	Compound	Retention Time	Area	% Area	Height
1		02:26.5	3707.1	100.0	1163.87

8a

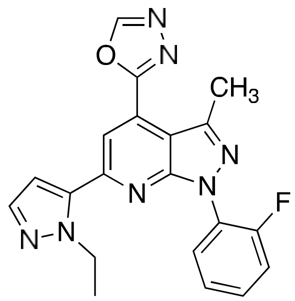

miniLC

Method: 6 MINUTE ISOCRATIC 80% acn
Batch: nbbb
Sample: MSU-SMQ-2-147
User: Administrator
S/N: 0118000000
Date: 07/13/2024 1:40 PM

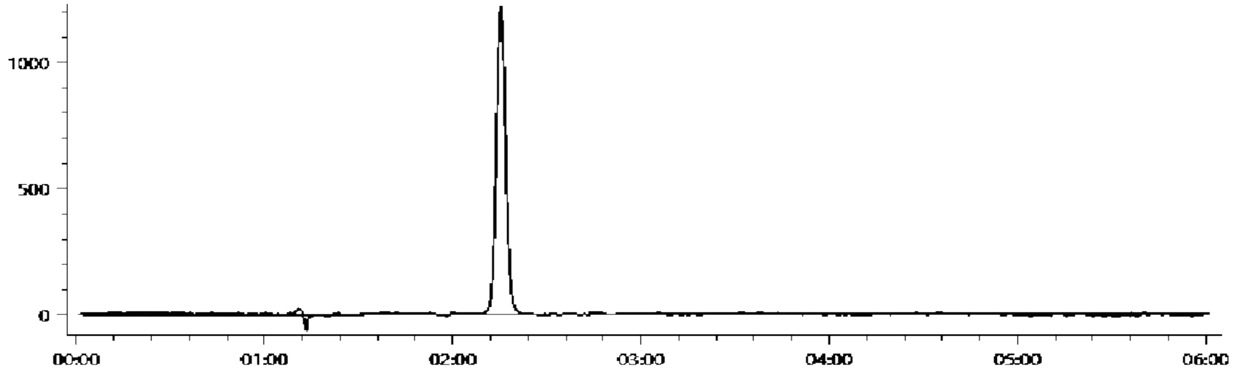

#	Compound	Retention Time	Area	% Area	Height
1		02:15.5	4634.9	100.0	1302.46

8b

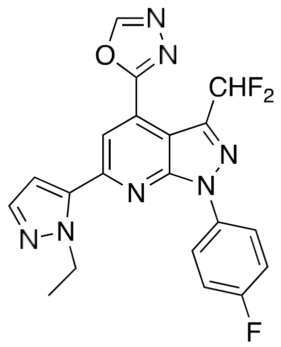

miniLC

Method: 6 MINUTE ISOCRATIC 80% acn
Batch: II
Sample: MSU-SMQ-3-65
User: Guest
S/N: 0118000000
Date: 09/29/2023 12:10 PM

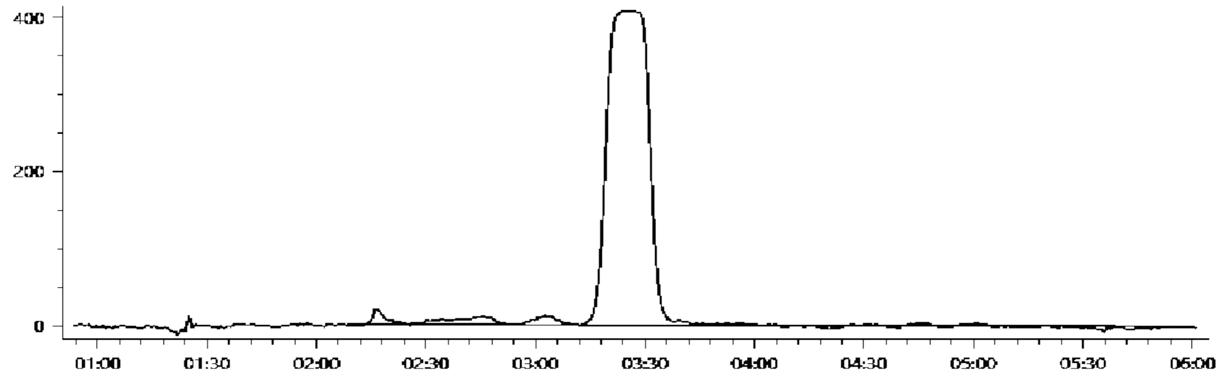

#	Compound	Retention Time	Area	% Area	Height
1		03:25.0	5552.5	100.0	408.32

8c

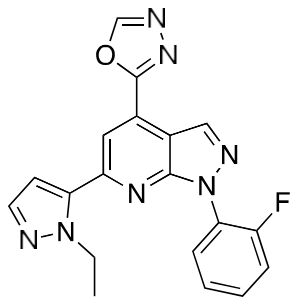

miniLC

Method: 6 MINUTE ISOCRATIC 80% acn
Batch: po
Sample: MSU-SMQ-2-149
User: Administrator
S/N: 0118000000
Date: 07/10/2024 4:06 PM

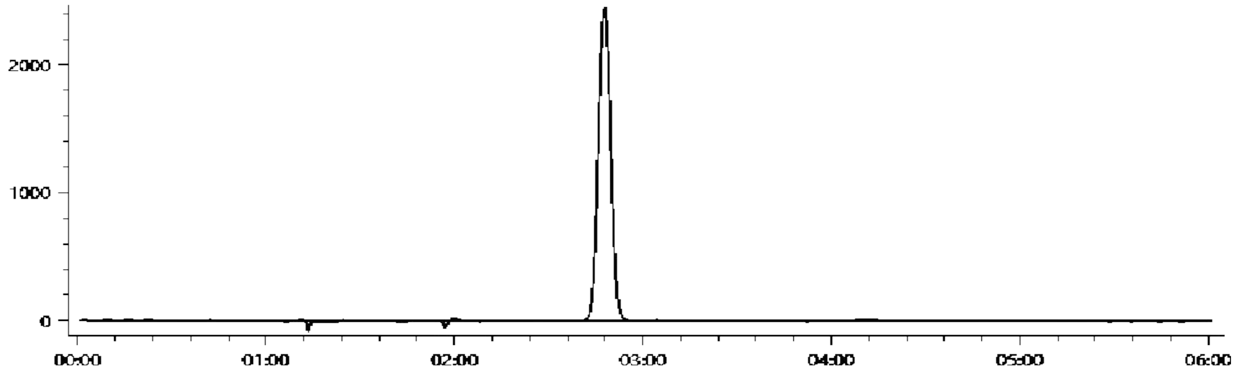

#	Compound	Retention Time	Area	% Area	Height
1		02:48.0	10909.4	100.0	2553.26

8d

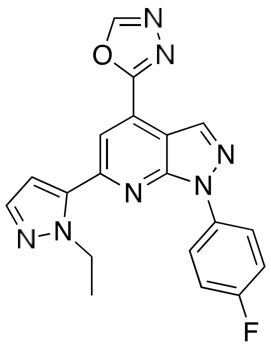

miniLC

Method: 6 MINUTE ISOCRATIC 80% acn
Batch: b
Sample: MSU-SMQ-2-153
User: Admin
S/N: 0118000000
Date: 05/02/2023 12:05 PM

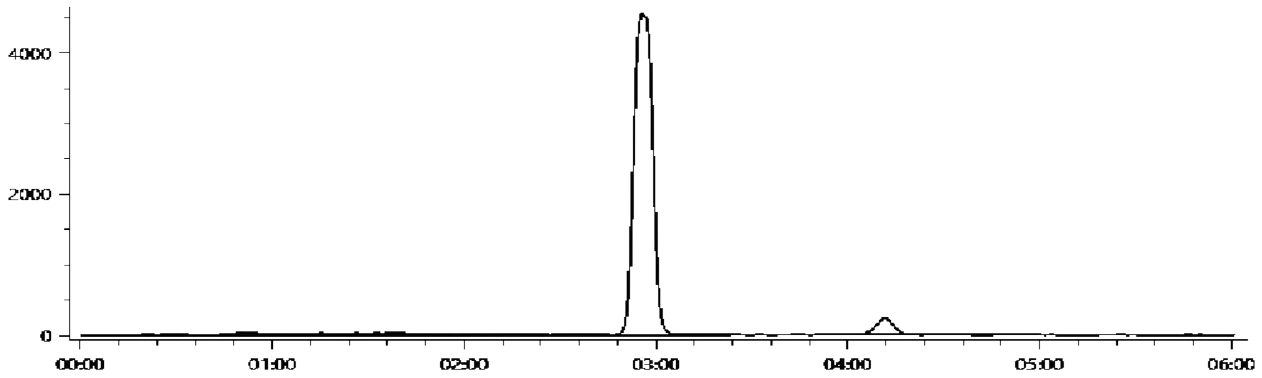

#	Compound	Retention Time	Area	% Area	Height
1		02:56.0	34091.3	95.5	4583.12
2		04:11.5	1379.5	4.5	230.87

8e

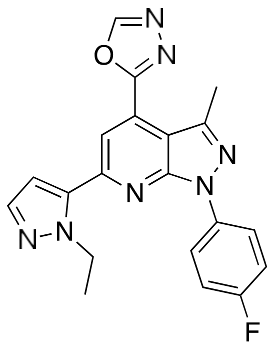

miniLC

Method: 6 MINUTE ISOCRATIC 80% acn
Batch: ppp
Sample: MSU-SMQ-2-157
User: Administrator
S/N: 0118000000
Date: 02/26/2024 6:47 PM

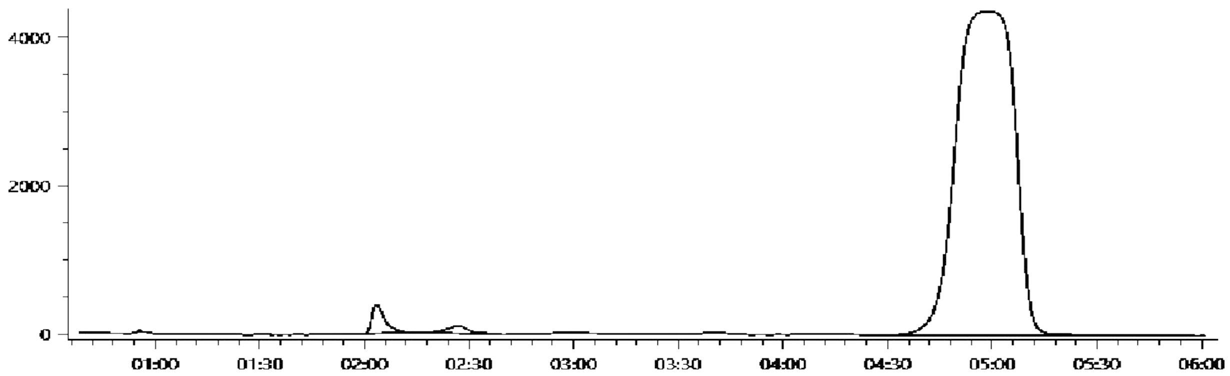

#	Compound	Retention Time	Area	% Area	Height
1		02:04.0	1655.1	1.9	406.46
2		02:26.5	754.0	0.9	141.60
3		04:59.0	84429.5	97.2	4381.30

8f

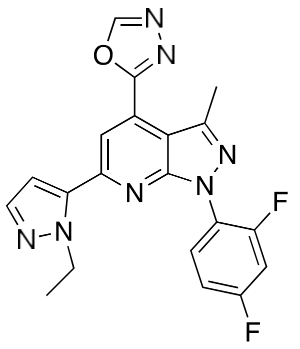

miniLC

Method: 6 MINUTE ISOCRATIC 80% acn
Batch: N
Sample: MSU-SMQ-2-170
User: Admin
S/N: 0118000000
Date: 05/22/2023 4:30 PM

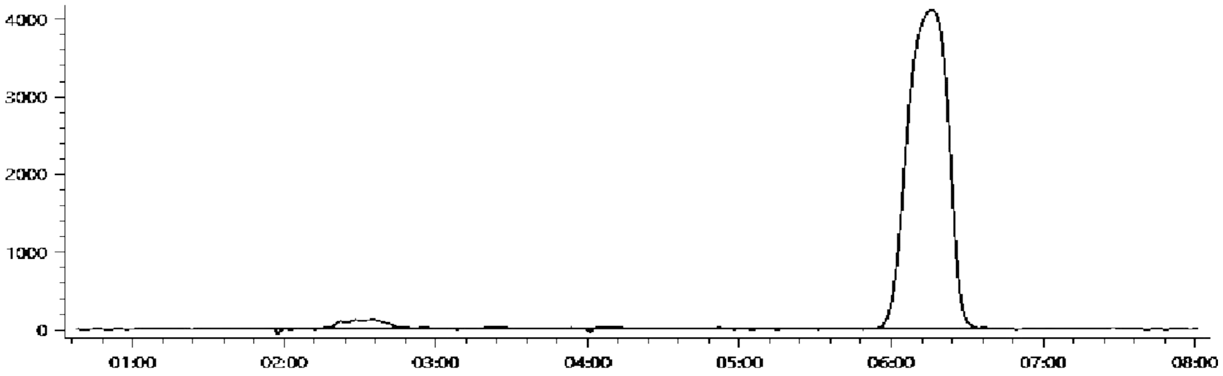

#	Compound	Retention Time	Area	% Area	Height
1		06:15.5	78498.2	100.0	4097.68

8g

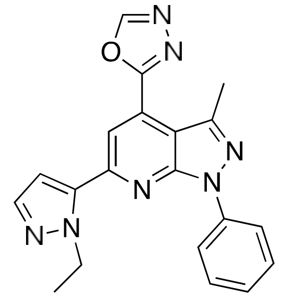

miniLC

Method: 6 MINUTE ISOCRATIC 80% acn
Batch: L
Sample: MSU-SMQ-2-178
User: Admin
S/N: 0118000000
Date: 06/06/2023 4:36 PM

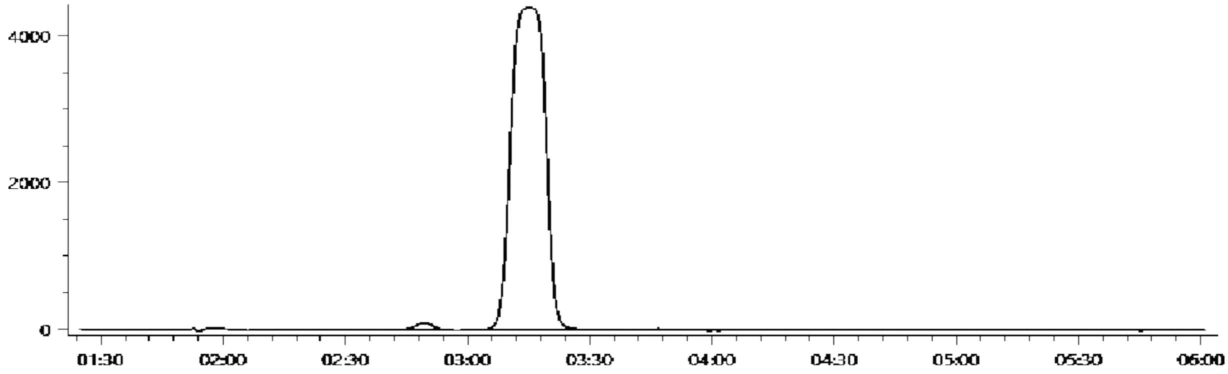

#	Compound	Retention Time	Area	% Area	Height
1		02:49.0	532.0	1.2	97.24
2		03:15.0	42629.4	98.8	4399.61

8h

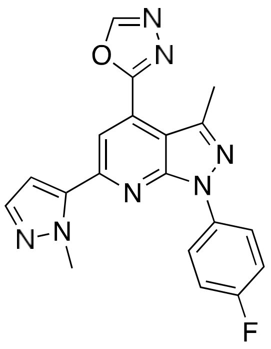

miniLC

Method: 6 MINUTE ISOCRATIC 80% acn
Batch: vb
Sample: MSU-SMQ-2-182
User: Administrator
S/N: 0118000000
Date: 07/10/2024 1:10 PM

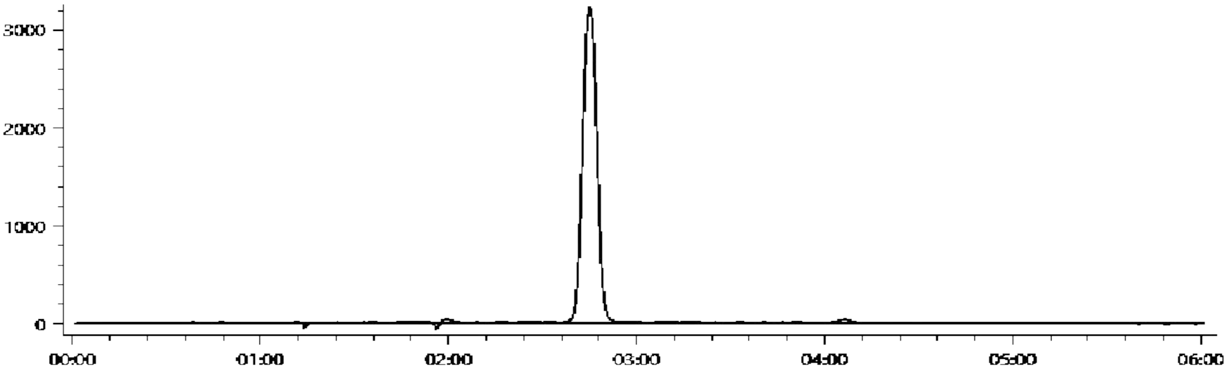

#	Compound	Retention Time	Area	% Area	Height
1		02:45.0	19358.2	100.0	3319.16

8i

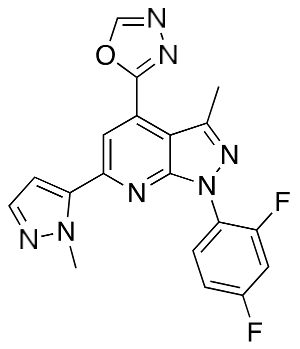

miniLC

Method: 6 MINUTE ISOCRATIC 80% acn
Batch: vb
Sample: MSU-SMQ-2-183
User: Administrator
S/N: 0118000000
Date: 07/10/2024 1:17 PM

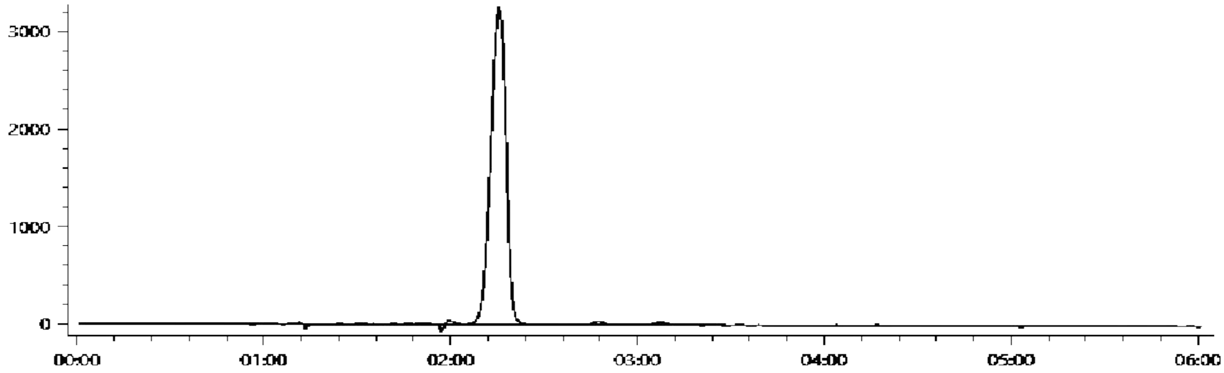

#	Compound	Retention Time	Area	% Area	Height
1		02:15.5	19187.4	100.0	3329.52

8j

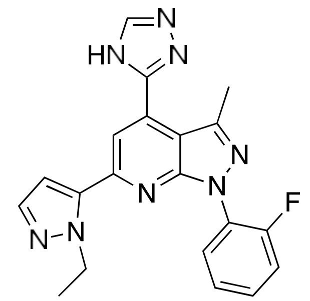

miniLC

Method: 6 MINUTE ISOCRATIC 80% acn
Batch: ui
Sample: MSU-SMQ-3-078
User: Administrator
S/N: 0118000000
Date: 07/09/2024 11:53 AM

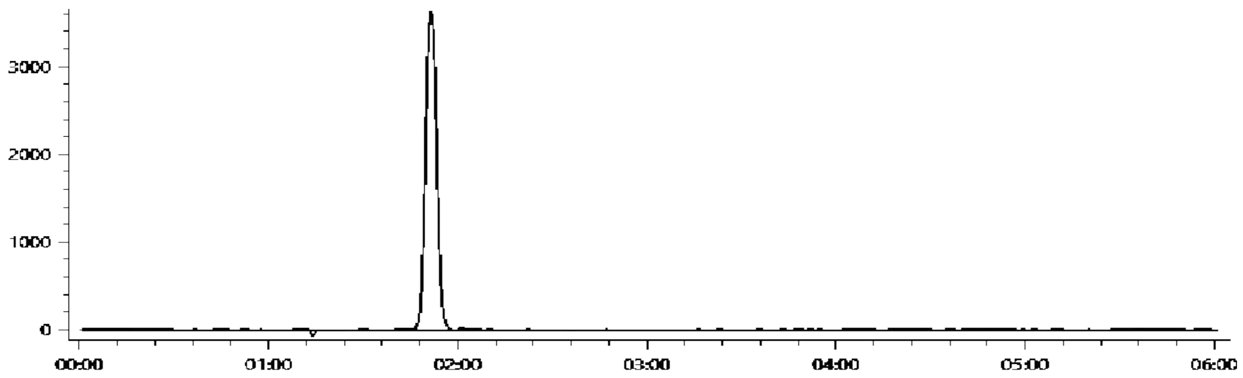

#	Compound	Retention Time	Area	% Area	Height
1		01:51.5	16245.6	100.0	3693.84

10a

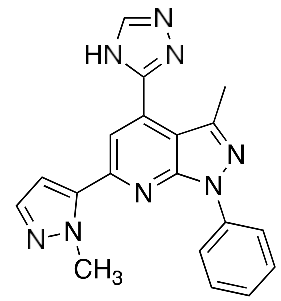

miniLC

Method: 6 MINUTE ISOCRATIC 80% acn
Batch: ■
Sample: MSU-SMQ-3-80
User: Administrator
S/N: 0118000000
Date: 02/26/2024 7:44 PM

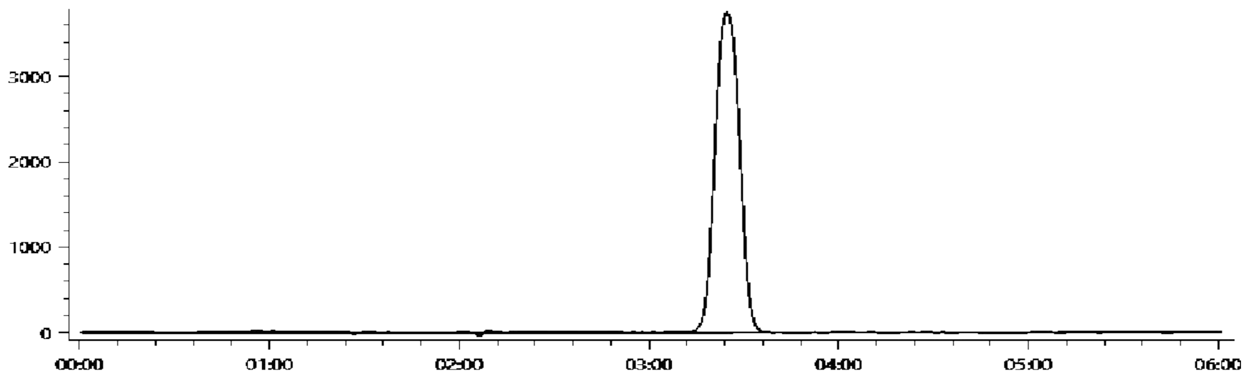

#	Compound	Retention Time	Area	% Area	Height
1		03:25.0	34716.4	100.0	3792.92

10b

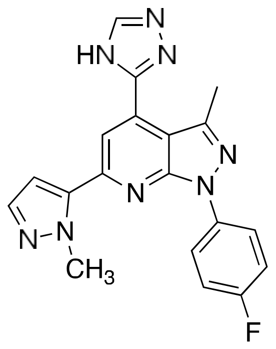

miniLC

Method: 6 MINUTE ISOCRATIC 80% acn
Batch: ppp
Sample: MSU-SMQ-3-81
User: Administrator
S/N: 0118000000
Date: 02/26/2024 7:00 PM

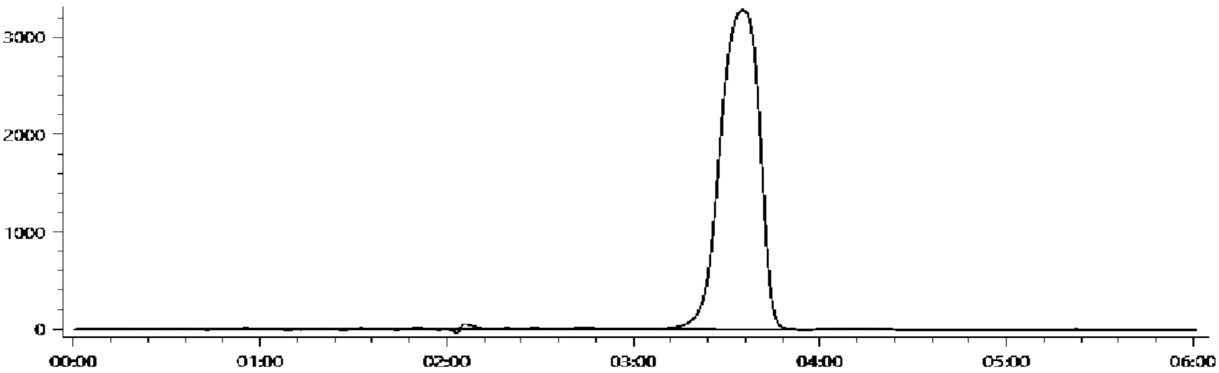

#	Compound	Retention Time	Area	% Area	Height
1		03:35.0	48212.3	100.0	3329.27

10c

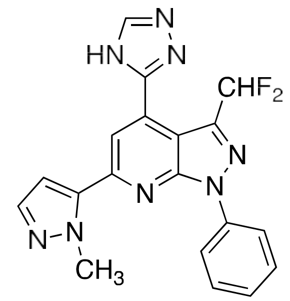

miniLC

Method: 6 MINUTE ISOCRATIC 80% acn
Batch: ui
Sample: MSU-SMQ-3-064
User: Administrator
S/N: 0118000000
Date: 07/09/2024 11:47 AM

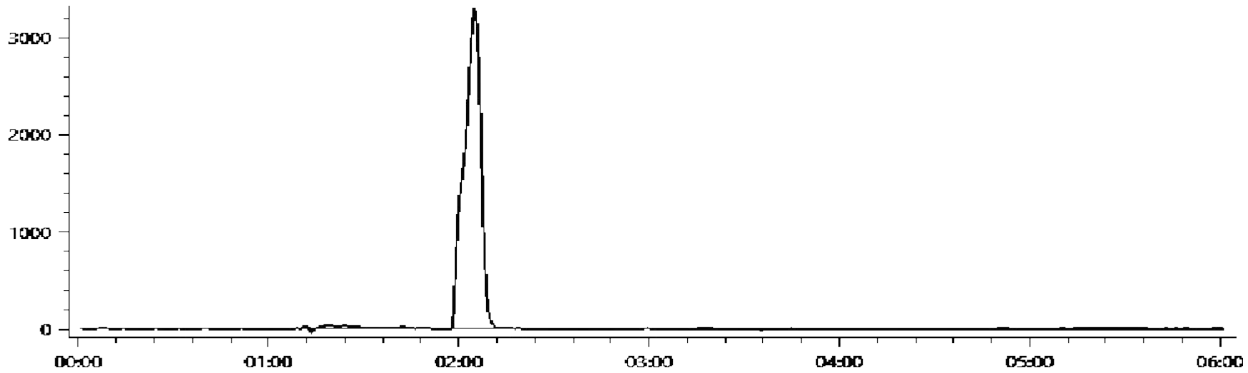

#	Compound	Retention Time	Area	% Area	Height
1		02:04.5	19901.1	100.0	3232.98

10d

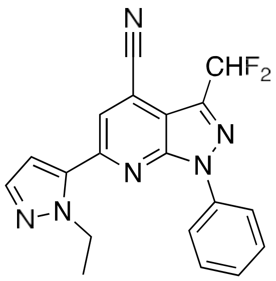

miniLC

Method: 6 MINUTE ISOCRATIC 80% acn
Batch: ppp
Sample: MSU-SMQ-2-122
User: Administrator
S/N: 0118000000
Date: 02/26/2024 7:06 PM

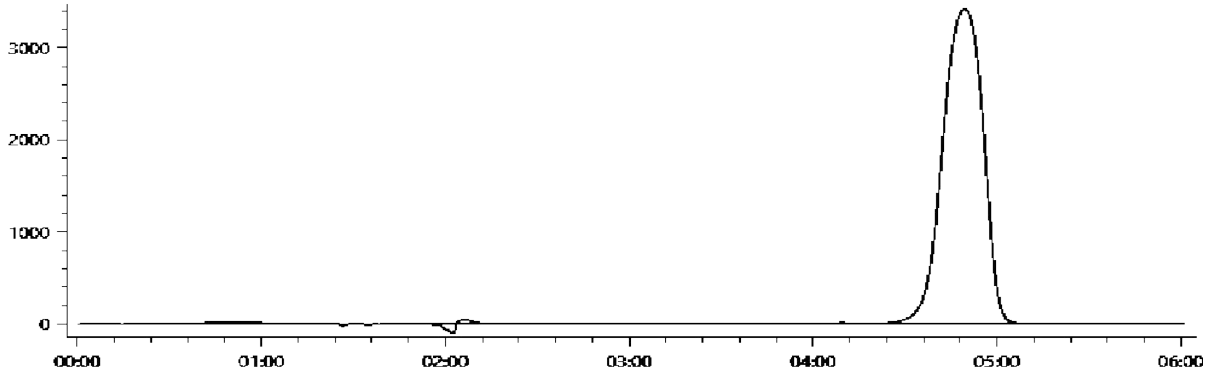

#	Compound	Retention Time	Area	% Area	Height
1		04:49.5	53546.7	100.0	3473.15

11

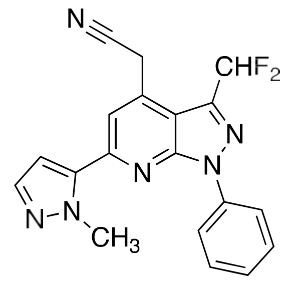

miniLC

Method: 6 MINUTE ISOCRATIC 80% acn
Batch: nbbb
Sample: MSU-SMQ-2-144
User: Administrator
S/N: 0118000000
Date: 07/13/2024 1:53 PM

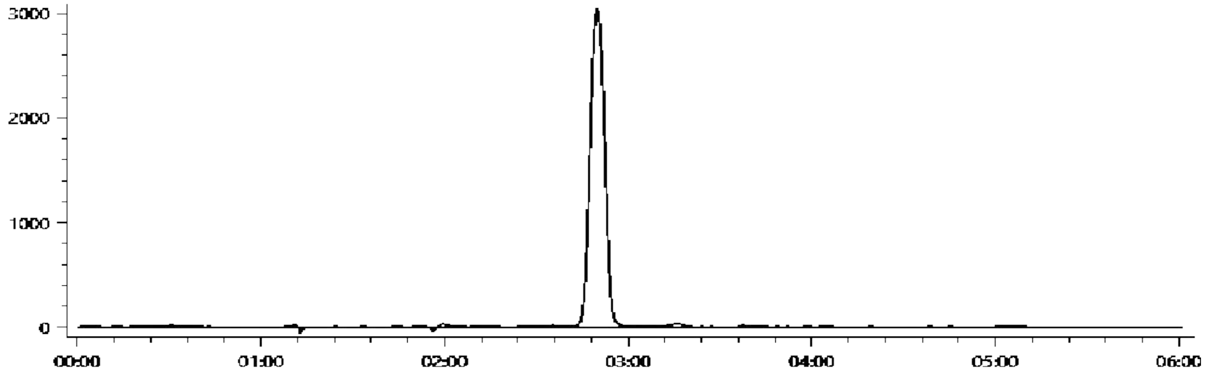

#	Compound	Retention Time	Area	% Area	Height
1		02:50.0	18459.8	100.0	3116.75

12
